# Safety and Immunogenicity of a Recombinant *Plasmodium falciparum* AMA1 Malaria Vaccine Adjuvanted with Alhydrogel™, Montanide ISA 720 or AS02

**DOI:** 10.1371/journal.pone.0003960

**Published:** 2008-12-18

**Authors:** Meta Roestenberg, Ed Remarque, Erik de Jonge, Rob Hermsen, Hildur Blythman, Odile Leroy, Egeruan Imoukhuede, Soren Jepsen, Opokua Ofori-Anyinam, Bart Faber, Clemens H. M. Kocken, Miranda Arnold, Vanessa Walraven, Karina Teelen, Will Roeffen, Quirijn de Mast, W. Ripley Ballou, Joe Cohen, Marie Claude Dubois, Stéphane Ascarateil, Andre van der Ven, Alan Thomas, Robert Sauerwein

**Affiliations:** 1 Radboud University Nijmegen Medical Centre, Nijmegen, The Netherlands; 2 Biomedical Primate Research Centre, Rijswijk, The Netherlands; 3 European Malaria Vaccine Initiative, Copenhagen, Denmark; 4 GlaxoSmithKline Biologicals, Rixensart, Belgium; 5 SEPPIC, Paris, France; Walter and Eliza Hall Institute of Medical Research, Australia

## Abstract

**Background:**

*Plasmodium falciparum* Apical Membrane Antigen 1 (*Pf*AMA1) is a candidate vaccine antigen expressed by merozoites and sporozoites. It plays a key role in red blood cell and hepatocyte invasion that can be blocked by antibodies.

**Methodology/Principal Findings:**

We assessed the safety and immunogenicity of recombinant *Pf*AMA1 in a dose-escalating, phase Ia trial. *Pf*AMA1 FVO strain, produced in *Pichia pastoris*, was reconstituted at 10 µg and 50 µg doses with three different adjuvants, *Alhydrogel™*, Montanide ISA720 and AS02 Adjuvant System. Six randomised groups of healthy male volunteers, 8–10 volunteers each, were scheduled to receive three immunisations at 4-week intervals. Safety and immunogenicity data were collected over one year. Transient pain was the predominant injection site reaction (80–100%). Induration occurred in the Montanide 50 µg group, resulting in a sterile abscess in two volunteers. Systemic adverse events occurred mainly in the AS02 groups lasting for 1–2 days. Erythema was observed in 22% of Montanide and 59% of AS02 group volunteers. After the second dose, six volunteers in the AS02 group and one in the Montanide group who reported grade 3 erythema (>50 mm) were withdrawn as they met the stopping criteria. All adverse events resolved. There were no vaccine-related serious adverse events. Humoral responses were highest in the AS02 groups. Antibodies showed activity in an *in vitro* growth inhibition assay up to 80%. Upon stimulation with the vaccine, peripheral mononuclear cells from all groups proliferated and secreted IFNγ and IL-5 cytokines.

**Conclusions/Significance:**

All formulations showed distinct reactogenicity profiles. All formulations with *Pf*AMA1 were immunogenic and induced functional antibodies.

**Trial Registration:**

Clinicaltrials.gov NCT00730782

## Introduction

In sub-Saharan Africa the burden of death and disease from *Plasmodium falciparum* malaria is particularly severe. To date, there are no approved vaccines to help reduce this burden, although a number of candidate vaccines have been put forward. The majority of the candidates target the pre-erythrocytic circumsporozoite protein (CSP) and the merozoite proteins Merozoite Surface Protein 1 (MSP1) and Apical Membrane Antigen 1 (AMA1)[1]. The RTS,S candidate vaccine has shown efficacy in infants and children [2], [3] and a phase III clinical trial is planned. The MSP1 and AMA1 candidate vaccines are in early stage clinical development and efficacy trials will provide information to determine whether these antigens are suitable targets, and whether they can be deployed singly or as components of a multivalent malaria vaccine.

Following an infected mosquito bite, *P. falciparum* sporozoites migrate to hepatocytes, each developing over a period of a week to release several thousand merozoites. These initiate cyclical asexual blood stage development, producing merozoites that invade erythrocytes. AMA1 is an integral membrane protein of merozoites and sporozoites and has a central role in parasite invasion of erythrocytes and potentially hepatocytes that can be inhibited by anti-AMA1 antibody [4]–[6]. In merozoites, AMA1 is synthesised as an 83 kDa molecule originally localised to the microneme. Around the time of merozoite release and the subsequent rapid erythrocyte invasion, the protein is N-terminally cleaved to a 66 kDa form. This translocates to the merozoite surface and undergoes secondary proteolytic processing, shedding soluble fragments (44 or 48 kDa) [7].

Immunisation with AMA1 can provide protection against infection in experimental animal models, and can induce antibodies that show functionality in *in vitro* growth inhibition assays (GIA). However, AMA1 is polymorphic and immune responses have varying degrees of strain specificity and growth inhibition [8].

Previous Phase I trials have shown that growth inhibitory antibodies can be induced by immunisation with *Pf*AMA1 [9], [10], but immunogenicity varied depending on the vaccine formulation. In particular, the choice of adjuvant has a major effect on the safety, stability, immunogenicity and, presumably, eventual efficacy of a vaccine [11]. Adjuvants can be tools that channel the immune reponse to generate high levels of the desired type of long-lived immunity. *Alhydrogel*™, an aluminum salt, is the most widely used adjuvant in licensed human vaccines and is therefore used as a standard to compare other adjuvants. Unfortunately, in combination with malaria antigens, it has generally induced poor reponses [12]–[15]. Montanide ISA 720, a squalene based water-in-oil adjuvant formulation has shown promising results in previous malaria vaccine trials [16]–[19], possibly due to the slow-release capacity of the inert water-in-oil emulsion and immune stimulating effects of its components [20]. AS02, a proprietary Adjuvant System from GlaxoSmithKline Biologicals based on an oil-in-water formulation, contains 50 µg each of the immunostimulants monophosphoryl lipid A (MPL) and Quillaja saponaria 21 (QS21) [21]. It has been used to adjuvant the RTS,S malaria candidate vaccine that targets CSP. To date, this candidate is the only malaria vaccine that has induced protection in adults, children and infants in natural field trials [22]–[25]. When combined with *Alhydrogel*™, RTS,S did not convey protection in a combined phase I/IIa trial [26]. AS02 is capable of eliciting high antibody titers along with strong cell-mediated immunity [27], both of which are believed to contribute to the efficacy of the RTS,S candidate vaccine [28].

Because of their central role in vaccine formulation, the development of adjuvants and delivery systems have become increasingly important. This study aims at comparing the safety and immunogenicity of *Pf*AMA1 in two dosages formulated with three different adjuvants in a phase Ia trial.

## Materials and Methods

### Vaccine preparation

Clinical grade *Pf*AMA1-FVO [25-545] was developed[29] and produced[30] as previously reported. In brief, FVO strain *Pf*AMA1 was codon adapted to expression in the methylotrophic yeast *Pichia pastoris*. Glycosylation sites were conservatively mutated, and the ectodomain comprising amino acids 25–545 was expressed. *Pf*AMA1-FVO[25-545] preparation was manufactured and lyophilised according to current good manufacturing practice in multidose vials containing either 120 µg (44 µg EDTA, 180 µg sucrose and 120 µg NaHCO_3_, lot B) or 62.5 µg (23 µg EDTA, 25 mg saccharose, 226 µg K_2_HPO_4_ and 187 µg NaH_2_PO_4_, lot C) of AMA1 that were stored between −18°C and −30°C and between +2 and +8°C respectively. Quality control and stability data are described by Faber et al. [31]. Reconstitution and mixing of vaccine with adjuvant was performed under sterile conditions under responsibility of the hospital pharmacist.

*Pf*AMA1 vaccine at 50 µg (high dose) and 10 µg (low dose) *Pf*AMA1 per injection (0.5 ml) was formulated with three different adjuvants and, after preparation, was kept at a constant temperature of +4°C for a maximum of six hours until injection. For the *Alhydrogel*™ formulation, 1.2 ml aluminum hydroxide suspension at 2 mg/ml (Statens Serum Institut (SSI), Copenhagen, Denmark) was added to the 120 µg *Pf*AMA1 vial (lot B) to obtain a high dose (50 µg in 0.5 ml) and 6 ml was added to obtain a low dose (10 µg in 0.5 ml) formulation. The resulting amount of aluminum in each vaccine was 0.5 mg. Stability studies confirmed adsorbtion of 99.9% of the antigen to the aluminum. Montanide formulations were prepared by dissolving the contents of the 120 µg *Pf*AMA1 vial (lot B) in sterile phosphate buffered saline (145 mM NaCl, 5 mM Phosphate, pH 7.4), 0.32 ml for the high dose and 1.6 ml for the low dose formulation. Montanide ISA 720 (SEPPIC, Paris, France) was subsequently added, 0.88 ml for the high dose to obtain 1.2 ml of formulation (50 µg *Pf*AMA1 in 0.5 ml) and 4.4 ml for the low dose to obtain 6 ml of formulation of which five 10 µg *Pf*AMA1 in 0.5 ml doses could be prepared. The suspension was prepared by manually pushing through a 22 gauge syringe coupling piece (3038068 Omnilabo International, Breda, The Netherlands) at +20C° for twenty up and down strokes.

The suspension was confirmed to be homogeneous and reached a median droplet size of approximately 1.5 µm (SD 0.17 µm) by particle size measurements with the Malvern Mastersizer S by SEPPIC.

For the AS02 formulation, the contents of one vial of lyophilized *Pf*AMA1 containing 62.5 µg of antigen (lot C) was mixed by gentle shaking with AS02 (approximately 0.6 ml) [32]. A 0.5 ml dose contained approximately 50 µg AMA-1 in 500 µl AS02 (high dose). For low dose preparations (10 µg) five times more AS02 adjuvant was added to the 62.5 µg vial of AMA1, from which five 0.5 ml low vaccine doses could be obtained.

### Study design

The protocol for this trial and supporting CONSORT checklist are available as supporting information; see Checklist S1 and Protocol S1 with Amendments S2, S3, S4, S5, S6 and S7. The study was designed as a dose-escalating phase Ia trial to assess the safety and immunogenicity of two dosages of *Pf*AMA1 with three different adjuvants. Volunteers were thus randomised into six different groups, each of which was aimed to constitute of a limited number of 10 volunteers for safety reasons. Randomisation was performed by an external statistician in six blocks through a computer program. Block randomization were used to ensure equal distribution of adjuvants among the immunisation groups. There was no stratification for sex and/or age. The randomization list was provided to the pharmacy departments. The clinical investigators allocated the next available number on entry into the trial. The code was revealed to the researchers once recruitment, data collection, and laboratory analyses were complete. The immunisations were thus performed blind, so neither volunteers, nor investigator or laboratory personnel were aware of the adjuvant allocation. Because of the dose-escalating design, the trial could not be blinded for dose.

For logistical reasons, the AS02 adjuvanted groups were immunised nine months after the *Alhydrogel*™ and Montanide groups, breaking the blind for this trial arm. A subsequent bias cannot formally be excluded but seems unlikely, since all trial procedures were identical. All immunisations were performed intramuscularly in the deltoid region of alternate arms at 0, 4 and 8 weeks.

### Participants

We aimed to recruit 60 healthy, malaria naïve male volunteers, aged between 18 to 45 years through advertisements at the Radboud University Nijmegen Medical Centre. Potential volunteers provided a medical history and a physical examination was conducted with routine laboratory tests consisting of full blood count, serum biochemistries and serologic assays for human immunodeficiency virus, hepatitis C and B virus. Volunteers were excluded from participation if they had any symptoms, signs or laboratory values suggestive of systemic illness, including renal, hepatic, cardiovascular, pulmonary, skin, immunodeficiency, psychiatric and other conditions, which could interfere with the interpretation of the study results or compromise the health of the volunteers, or received chronic medication, had a history of drug or alcohol abuse interfering with social function one year prior to enrolment, or a known hypersensitivity to any of the vaccine components. Additional reasons for exclusion were a history of malaria or residence in malaria endemic areas within the past six months, previous participation in a malaria vaccine trial or receiving vaccines other than the study vaccines. Furthermore, volunteers were not enrolled in any other clinical trial, and agreed to remain available to be closely monitored. All volunteers provided written informed consent. The study was approved by the Institutional Review Board (CMO Regio Arnhem-Nijmegen, 2005/015). The study was conducted in accordance with the Declaration of Helsinki principles for the conduct of clinical trials and the International Committee of Harmonization Good Clinical Practice Guidelines [33] and registered at www.clinicaltrials.gov (NCT00730782).

### Assessment of safety

Volunteers were observed for 30 minutes and evaluated on days 1, 3, 7 and 14 after every immunisation. At each visit, local and systemic reactogenicity was assessed by a physician and findings recorded and scored as follows: grade 1, mild reaction (easily tolerated), grade 2, moderate reaction (interferes with normal activity), or grade 3, severe reaction (prevents normal activity). Redness, swelling and induration (according to Brighton collaboration definitions, www.brightoncollaboration.org) were measured with a ruler, and categorised according to the longest diameter as grade 1: ≤20 mm, grade 2: >20 and ≤50 mm, grade 3: >50 mm. Temperature was measured with an oral thermometer; fever intensity was defined as grade 1 (37.5°C to 38°C), grade 2 (>38°C to 39°C) or grade 3 (>39°C). The following adverse events were solicited and recorded routinely during the 14 days after immunisation: injection site pain, redness and swelling, systemic fatigue, fever, headache, malaise, myalgia, joint pain, gastrointestinal symptoms and contralateral local reactions.

### Blood samples

Safety was also determined by serial laboratory evaluations of clinical chemistry and haematology on blood samples collected 7 and 28 days after immunisation. For evaluation of immunogenicity, blood was collected in Vacutainer CPT tubes (Becton and Dickinson) and processed within two hours after collection on immunisation days, one month after each immunisation and on Days 140 and 365. Plasma was collected after centrifugation (2000 g 15′) aliquoted and stored at −20°C for antibody analysis (ELISA, Immuno Fluorescence Assay (IFA) and Growth Inhibition Assay (GIA)). Peripheral blood mononuclear cells (PBMC) were collected, washed in PBS (800 g, 10 min) and immediately used for assays (lymphocyte stimulation assay and ELISPOT).

### Measurement of anti-AMA1 antibodies by ELISA and IFA

Antibody to *Pf*AMA1 was measured using a standardized ELISA protocol. All procedures used Phosphate buffered saline (PBS) and for washing steps 0.05% Tween 20 (Sigma-Aldrich). Briefly, wells in 96-well polystyrene plates (NUNC Maxisorp, Sanbio), were coated overnight (100 µl, 0.5 µg/ml *Pf*AMA1, 4°C), washed (3×), blocked (60 min, 3% BSA (Sigma-Aldrich)) and washed (3×) before addition of 100 µL from duplicate dilution series (diluted in PBS-Tween BSA, one hour, +37°C). After washing (3×) goat anti-human IgG alkaline phosphate (Perbio Science) diluted 1∶1250 in 0.5% BSA, 0.05% Tween was added, (one hour, +37°C). Plates were washed and 100 µl of 1 mg/ml para-nitro-phenyl-phosphatase (Fluka, Sigma-Aldrich) substrate was added (30 minutes, room temperature). A human plasma pool from a malaria endemic area was used as reference positive control, whereas a plasma pool from eight healthy malaria-naive Dutch volunteers was used as a negative control. Optical density was measured at 405 nm. Variation between duplicates was set to a maximum of 15%. Measurements with a greater variation were repeated. The standard curve of human plasma pool from a malaria endemic area, defined to contain 400 Arbitrary Units, was fitted to a four-parameter hyperbolic function, using the ADAMSEL program (E. Remarque, unpublished work). Using this standard curve, optical density from samples were converted to Arbitrary Units (AU). Test samples that did not fall within the linear part of the optical density range of the standard were tested at alternate dilutions.

IFA was performed on cultured *P. falciparum* parasitized red blood cells. Ten well black slides (30-966-A black, Nutacon, The Netherlands) were coated with a washed parasite suspension of 3×10^6^ parasites/ml, air dried and kept at −80°C until used. FCR3 parasites, expressing an AMA1 protein with one amino-acid difference from the FVO parasites, and NF54 strain parasites, with 26 amino-acid difference in AMA1 protein were used to prepare slides.

Based on antibody titers by ELISA on day 84, a representative sample of fifteen sera was selected for IFA, containing at least two samples from each adjuvant group and at least three samples with low, intermediate or high ELISA titers. Before use slides were brought to room temperature in an evacuated exicator. Plasma was diluted in PBS (1∶40, 1∶80, 1∶160, 1∶320, 1∶640) and a final volume of 20 µL was added to the wells and incubated for 0.5 hour, at room temperature. As for the ELISA protocol, the malaria-naïve blood bank donor plasma pool was used as a negative control and human malaria endemic plasma was used as a positive control. After washing (2× in PBS) and air drying, slide samples were incubated with rabbit anti-human Immunoglobin FITC (F0200, DAKO, Denmark) in 0.05% w.v Evans Blue (3169, Merck), PBS for 30 minutes at room temperature. Slides were washed twice and incubated for 15 minutes with DAPI (4′-6-Diamindino-2-phenylindole, 24653, Merck, Darmstadt, Germany), 5 µg/ml in PBS. After washing (2× in PBS) slides were mounted with Vectashield Mounting Medium (H-1000, Brunschwig, Amsterdam), covered with a deck-slide and read immediately by two independent blinded examiners. Examiners identified the highest dilution still showing a staining pattern above the background of pre-immunisation samples. Differences between examiners were never greater than one dilution and the mean of both dilutions was taken.

### ELISPOT for IFNy and IL-5

ELISPOT was performed according to manufacturer's instructions (Becton and Dickinson Elispot Set Human IFNγ or IL-5). In summary, plates provided in the set were coated with either IFNγ or IL-5 capture antibody (5 µg/ml, overnight, 4°C). After blocking with complete medium solution (RPMI 1640 (Invitrogen) containing 10% Fetal Bovine Serum (FBS Invitrogen, Breda, The Netherlands), 1% Glutamax (Invitrogen), 1% Penicillin-Streptomycin (GIBCO-BRL, Invitrogen), 1% MEM) 100 µl of 10^5^ PBMC suspension and 100 µl of *Pf*AMA1 containing either 60 µg, 12 µg or 2.4 µg was added per well. Positive controls were stimulated with Tetanus Toxoid 10 µg/ml (RIVM, Bilthoven, The Netherlands) and phytohaemagglutinin 5 µg/ml (PHA-L Sigma-Aldrich) end concentration. Negative controls were incubated with complete medium solution (mean SFC/10^5^ cells 31±17 for IFNγ and 9±7 for IL-5). After incubation (40 hours, 37°C in humidified 5% CO_2_), biotinylated anti human IFNγ and IL-5 (0.25 µg/ml and 2 µg/ml, respectively), containing 10% FBS was added (two hours at room temperature). Streptavidin-HRP was used as an enzyme conjugate. Detection was performed with the Becton and Dickinson AEC Substrate Reagent Set, according to manufacturer's instruction. Spot-forming cell numbers were counted by ELISPOT reader (4 Microtiter Plate Reader, AELVIS, Sanquin, Amsterdam) and analysed by the ELISPOT Analysis Software Version 4.0 (Sanquin, Amsterdam). All measurements were performed in triplo. Variation between triplicates was set to a maximum of 20%.

### Lymphocyte Stimulation Assay

Lymphocyte stimulation assays were performed as described previously [34]. Peripheral blood mononuclear cell suspension (PBMC) was diluted to 1×10^6^ PBMC per ml in Dulbecco's MEM (DMEM) with Glutamax-I, 2 mM pyruvate and high Glucose (GIBCO BRL, Invitrogen) supplemented with 10 mM HEPES buffer (GIBCO BRL, Invitrogen), 100 IU/mL Penicillin-Streptomycin (GIBCO BRL, Invitrogen), 100 µM non-essential aminoacids (GIBCO BRL, Invitrogen) and 2.5% human AB serum (AB) (Bodinco BV, Alkmaar, The Netherlands). 100 µl of PBMC was added to 100 µl *Pf*AMA1 (30, 6 or 1.2 µg/ml in PBS) in 96 well Nunclon surface flat plates (Life Technology). Plates were incubated (six days, +37°C, humidified 0.5%CO2) before labelling (10 µl ^3^H thymidine, 0.25 uCi per well, 24 hours) and harvested onto Wallac filter mats using the Wallac Beta plate harvester. Incorporated ^3^H-thimidine was determined using a Wallac Beta Plate counter. Stimulation indices (SI) were calculated relative to control wells to which no *Pf*AMA1 had been added. PBMC were tested in parallel for their ability to be stimulated with Tetanus Toxoid (Purified Tetanus Toxoid 150 Lf/ml, RIVM, Bilthoven, The Netherlands) and phytohaemagglutinin 5 µg/ml (PHA-L, Sigma-Aldrich).

### In vitro parasite growth inhibition

Antibodies to be used for parasite inhibition assays were purified on protein A columns (Immunopure Plus Pierce, St Louis, MO, USA) using standard protocols, exchanged into RPMI 1640 using Amicon Ultra-15 concentrators (30 kDa cutoff, Millipore, Ireland), filter-sterilised and stored at −20°C until use. IgG concentrations were determined using a Nanodrop ND-1000 spectrophotometer (Nanodrop Technologies, Wilmington, DE, USA).

*P. falciparum* strain FCR3 was cultured *in vitro* using standard *P.falciparum* culture techniques in an atmosphere of 5% CO_2_, 5% O_2_ and 90% N_2_. FCR3 AMA1 (accession no. M34553) differs by one amino acid in the pro-sequence from FVO AMA1 (accession no. AJ277646).

The effect of purified IgG antibodies on parasite invasion was evaluated with two IgG concentrations (5 and 10 mg/mL, respectively) in triplicate using 96 well flat-bottomed plates (Greiner) with synchronized cultures of *P. falciparum* schizonts at a starting parasitemia of 0.2–0.4%, a haematocrit of 2.0% and a final volume of 100 µL containing 10% control non-immune human serum, 20 µg mL^−1^ gentamicin in RPMI 1640. After 40 to 42 hours, cultures were resuspended, and 50 µL was transferred into 200 µL ice-cold PBS. The cultures were then centrifuged, the supernatant removed and the plates were frozen. Inhibition of parasite growth was estimated using the pLDH assay as previously described (14). Parasite growth inhibition, reported as a percentage, was calculated as follows: 100−((Od_experimental_−Od_background_)/(Od_control_−Od_background_)×100). IgG purified from plasma before immunisation was used as a control, and culture medium was used to measure the background Od.

### Statistical methods

Safety analyses were based on intention to treat data selection (n = 56). For immunology assays, per protocol analyses were used (n = 47). Between group differences were calculated by one-way ANOVA, using post-hoc Bonferroni when p<0.05. Differences between high and low dose groups were compared with Mann-Whitney U test.

## Results

### Study population

**Figure 1:**
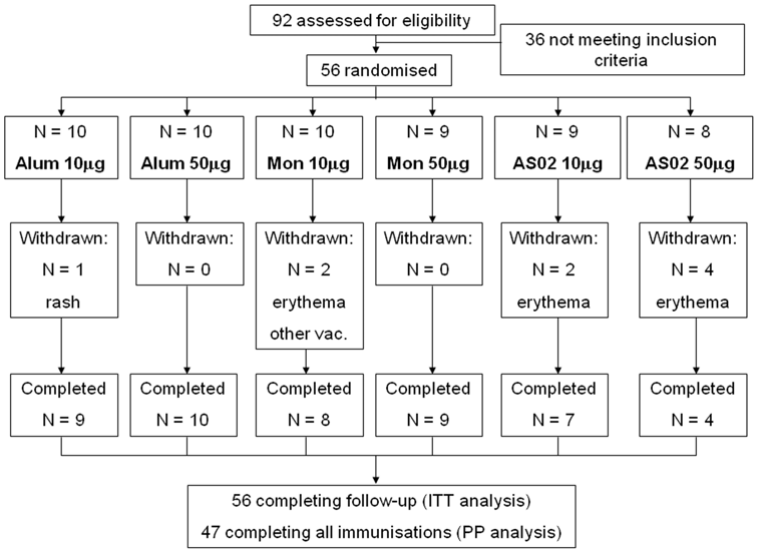
Study flow chart showing number of volunteers randomised, withdrawn and completing follow-up. Coding for adjuvant as follows: Alum = *Alhydrogel*™, Mon = Montanide. Reasons for withdrawal are given: “rash” = allergic rash unrelated to study procedure, “erythema” = grade 3 injection site erythema leading to withdrawal, “other vac.” = concomitant Hepatitis B vaccination leading to exclusion.

Participants were recruited at the Radboud University Nijmegen Medical Centre from September to October 2005. Of 92 adult males screened in and having provided informed consent, 56 were eligible and enrolled (fig. 1). Main reasons for exclusion were abnormal laboratory parameters or unable to be closely monitored for social, geographic or psychological reasons. Table 1 shows the demographics of volunteers per randomised group. The mean age was 23 years old (range 18–42 years) and all but one were Caucasian.

**Table 1:** Demographic data, race and age of volunteers per dose and adjuvant group.

Adjuvant		Aluminum		Montanide		AS02		All
*Pf*AMA1 dose		10 µg	50 µg	10 µg	50 µg	10 µg	50 µg	
N		10	10	10	9	9	8	56
**Race**	Caucasian	10	10	10	9	8	8	55
	Oriental	-	-	-	-	1	-	
**Age (years)**	Mean	22.4	22.6	22.5	22.6	24.1	24.4	23.0
	STD	3.1	2.0	4.5	3.8	7.3	6.1	4.6
	Minimum	18	19	18	19	19	18	18
	Maximum	29	26	33	31	42	36	42

### Safety and reactogenicity

No serious adverse events occurred that were definitely, probably, or possibly related to immunisation. No clinically relevant changes in vital signs or laboratory values were reported throughout the study. Forty-seven volunteers (84%) received all three immunisations; nine were excluded for one or more immunisations (fig. 1). Two of these were excluded for reasons unrelated to the trial procedures. One (Alhydrogel™ 10 µg group) developed a generalised rash assessed as unrelated to the vaccine between the first and second immunisations and one (Montanide 10 µg group) received a concomitant hepatitis B immunisation. Seven volunteers were excluded because they developed grade 3 erythema (diameter >50 mm) after the second immunisation; one in the Montanide 10 µg group, the other six in the AS02 groups (two in the 10 µg group, four in the 50 µg group).

Volunteers in all groups presented with local injection site reactions, the most predominant being transient mild to moderate pain (80–100%, table 2). Erythema was commonly observed (10 of 17 volunteers) in the AS02 adjuvanted groups, occurring after the second and third immunisation. In the Montanide group 4 of 18 volunteers developed erythema. Seven volunteers reported grade 3 erythema and were withdrawn from further immunisation after dose 2. The skin in grade 3 (diameter >50 mm) erythema was not painful and did not limit daily activities. Episodes of erythema generally lasted 2–3 days.

**Table 2:** Number of volunteers reporting vaccine related adverse events per dose and adjuvant group.

Adjuvant	Alum		Montanide		AS02		Total
*Pf*AMA1 dose	10 µg	50 µg	10 µg	50 µg	10 µg	50 µg	
N	10	10	10	9	9	8	56
Total	8 (80.0%)	10 (100%)	9 (90.0%)	9 (100%)	9 (100%)	8 (100%)	53 (94.6%)
**LOCAL**							
Pain	8 (80.0%)	10 (100%)	8 (80.0%)	9 (100%)	9 (100%)	8 (100%)	52 (92.9%)
Erythema	-	-	2 (20.0%)	2 (22.2%)	4 (44.4%)	6 (75.0%)	14 (25%)
Swelling	-	-	1 (10.0%)	-	3 (33.3%)	1 (12.5%)	5 (8.9%)
Induration	-	-	1 (10.0%)	2 (22.2%)	-	-	3 (5.4%)
Sterile abscess	-	-	-	2 (22.2%)	-	-	2 (3.6%)
**SYSTEMIC**							
Headache	1 (10.0%)	-	2 (20.0%)	-	6 (66.7%)	7 (87.5%)	16 (28.6%)
Malaise	-	-	-	1 (11.1%)	6 (66.7%)	7 (87.5%)	14 (25.0%)
Fever	-	-	-	-	5 (55.6%)	5 (62.5%)	10 (17.9%)
Myalgia	-	-	-	-	4 (44.4%)	2 (25.0%)	6 (10.7%)
Nausea	1 (10.0%)	-	-	-	1 (11.1%)	2 (25.0%)	4 (7.1%)
Fatigue	-	-	-	-	-	2 (25.0%)	2 (3.6%)
Arthralgia	-	-	-	-	1 (11.1%)	-	1 (1.8%)
Abdominal pain	-	-	-	-	1 (11.1%)	-	1 (1.8%)

Induration at the site of injection occurred in three volunteers in the course of the study (table 2). One volunteer, in the Montanide 10 µg group, developed moderate induration 15 days after the first immunisation, lasting for five days. The second and third immunisations in this volunteer were well tolerated; induration did not re-appear. Another volunteer developed induration starting nine days after the first immunisation in the left arm with 50 µg *Pf*AMA1 in Montanide, lasting 25 days. The second immunisation was well tolerated, but the left arm induration re-appeared one day after the third immunisation, accompanied by pain and induration at the previous immunisation site in the contralateral (right) arm. Four weeks later, the induration became soft and fluctuated, indicating abcess formation. A total of 63 ml of opaque, brown fluid was aspirated by two subsequent punctures, after which the abscess and induration resolved spontaneously and disappeared completely at 81 days post third immunisation. The third volunteer, also in the 50 µg *Pf*AMA1 adjuvanted with Montanide group, developed moderate induration nine days after the second immunisation which lasted approximately one week. Six days after the third immunisation he developed induration at his left arm (the site of the first and last immunisation) which eventually started fluctuating. A total of 130 ml brown, opaque fluid was collected by means of two punctures. Thereafter, spontaneous percutaneous drainage occurred and the lesion resolved 57 days after the third immunisation. Both volunteers did not have any systemic symptoms such as fever during this time period. Abcesses were only mildly painful, but limited volunteers daily activities because of their size.

The aspirated fluids from both volunteers were abundant in red blood cells and lymphocytes with low Creatinine Kinase (CK) levels. Repeated cultures did not reveal any bacterial contamination. Serum CK levels were normal. Circulating levels of C-reactive protein remained below detection levels (indicating that the reaction was a local response). Ultrasound examination suggested an intramuscular and subcutaneous localisation of the fluid-filled cavity.

Systemic reactions were infrequent in the Alhydrogel™ and Montanide groups and occurred mainly in the AS02 groups. The systemic adverse events occurred within 24 hours of immunisation and usually resolved within two days. The most prevalent systemic adverse events were headache (77.8–87.5%) and malaise (66.7–87.5%) in the AS02 groups. Four of those volunteers reported grade 3 headache or malaise. Most of the systemic adverse events occurred after dose 2. There was no effect of antigen dose on reactogenicity. No changes in blood pressure were noted in any of these volunteers.

### Humoral immune response

**Figure 2:**
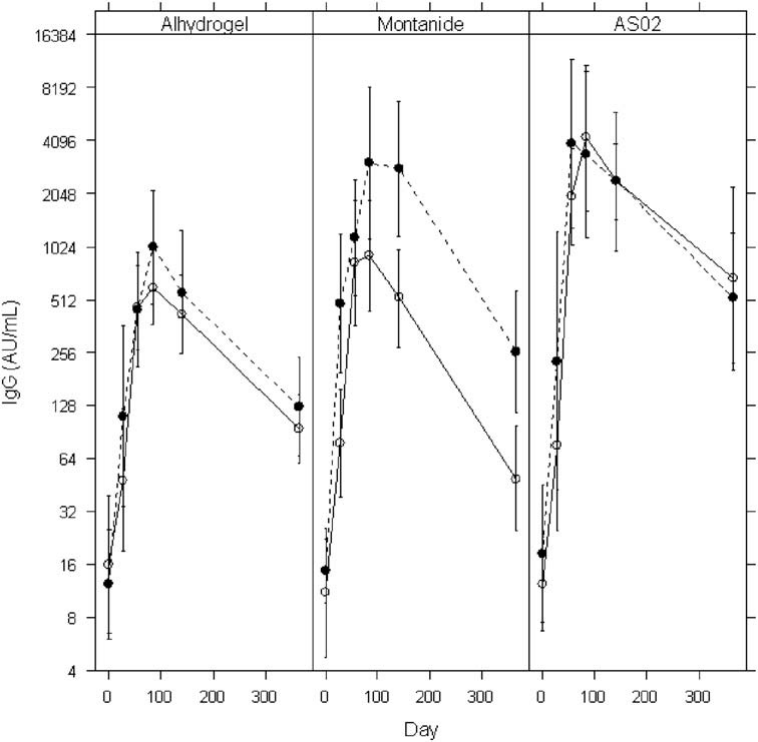
Mean log anti-AMA-1 titers with standard error of the mean for low and high dose per adjuvant group. Anti-AMA-1 titers were determined by ELISA for the six different groups, immunized with *Alhydrogel*™, Montanide and AS02 adjuvanted *Pf*AMA1 vaccine. Dashed lines represent high dose of *Pf*AMA1 (50 µg), continuous lines represent low dose groups (10 µg). Measurements were performed at baseline, 28 days after the first, second and third immunisation (day 28, 56 and 84 respectively) and day 140 and 365.

Peak antibody titers were observed one month after the final immunisation. 100% of volunteers in the 10 and 50 µg AS02 and 50 µg Montanide groups showed a greater than four-fold increase in antibody titer over pre-immunisation compared to 60% in the 10 µg Alhydrogel™, 80% in the 50 µg Alhydrogel™ and 90% in the 10 µg Montanide groups (fig. 2). All vaccinees had reached IgG titers comparable to or higher than semi immune sera. Two and four months post final immunisation both AS02 groups and the Montanide 50 µg group showed the highest IgG titers but given the small sample sizes there was no power to detect statistical differences between groups. Antibody titers decreased further one year post immunisation, with the steepest decline being in the Montanide groups, to a level comparable with the reference Alhydrogel™ groups. One year post vaccination, titers in the 10 µg AS02 group were significantly higher as compared to the reference group (post hoc Bonferroni when compared with low dose Alhydrogel™ reference group p<0.01, 95% CI 0.25 to1.5). Vaccinees receiving 50 µg PfAMA1 generally showed a trend towards higher antibody titers than the corresponding 10 µg group, except for the AS02 groups where antibody titers were not antigen dose-dependent.

**Figure 3:**
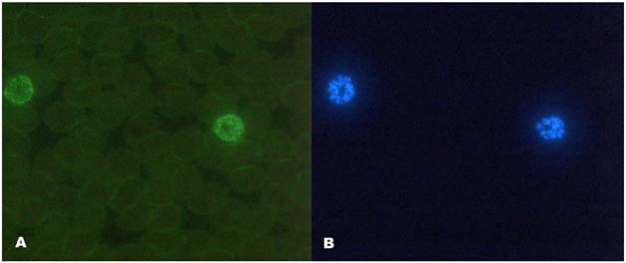
Representative immunofluorescent microscopy picture, showing recognition of native antigen on merozoites by induced anti-AMA1 antibodies. Immunofluorescence picture of merozoites incubated with 40× diluted anti-AMA1 plasma from an immunized volunteer one month after final immunisation stained with Rabbit anti-human immunoglobulin FITC (A) and DAPI (B). Photo was taken at magnification 400×. Incubation with monoclonal antibody 4G2, a pan-specific anti-AMA1 antibody, confirmed the surface staining pattern (not shown).

Sera from vaccinees could be shown to recognise the native *Pf*AMA1 by immunofluorescence in a dose dependent manner. Eight of fifteen samples were positive in IFA, amongst which were four samples with the highest antibody titers. The staining pattern found in positive samples localised to the same structures as 4G2 rat monoclonal antibody (fig. 3).

### Cellular immune response

**Figure 4:**
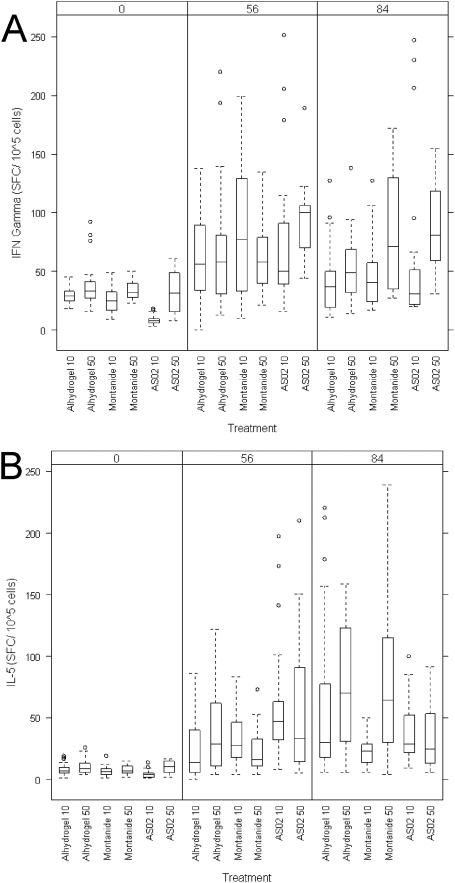
ELISPOT assay for IFNγ (A) and IL-5 (B) after stimulation with 6 µg *Pf*AMA1. Peripheral Blood Mononuclear Cells from immunised volunteers 28 days after the second immunisation and 28 days after the third immunisation (day 56 and 84 respectively) were stimulated with 6 µg of *Pf*AMA1 vaccine. Production of IFNγ and IL-5 was measured by counting spots in ELISPOT plates. Box plots and whiskers show the range and the 25^th^, 50^th^ and 75^th^ percentile of spots per 2×10^5^ cells. Circles represent outliers.

In all groups, induction of IFNγ and IL-5 cytokines could be demonstrated (fig. 4). The magnitude of cytokine production was not dose dependent or dependent on the number of immunisations. Rather, IFNγ production in many samples decreased after the third immunisation. For both cytokines, *Pf*AMA1 induction was comparable or higher than that following stimulation with 5 µg Tetanus Toxoid (data not shown). Cytokine production in the different groups did not differ significantly from each other (for INFγ p = 0.18, for IL-5 p = 0.14). Ratio's of IFNγ / IL-5 production were also not significantly different between adjuvant groups (data not shown), but showed a trend towards higher ratio in the Montanide and AS02 groups (Day 84 mean ratio Alhydrogel: 1.16 (95% CI: 0.08 to 2.23), Montanide: 2.82 (95% CI: 1.44 to 4.21), AS02: 2.66 (95% CI: 0.57 to 4.76)).

**Figure 5:**
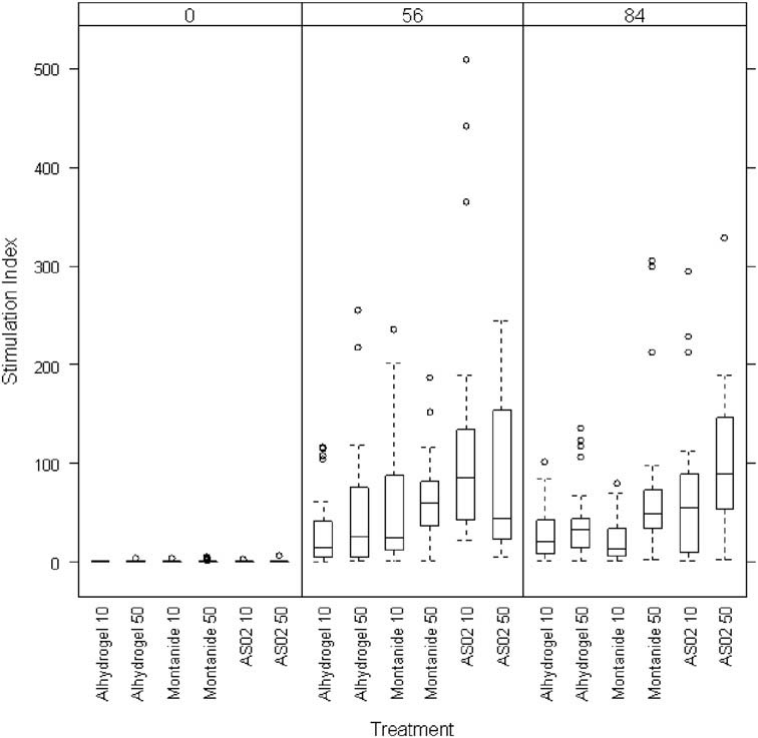
Stimulation indices in response to 6 µg/ml *Pf*AMA1 presented as box plots and whiskers. Peripheral Blood Mononuclear Cells from immunised volunteers 28 days after the second immunisation and 28 days after the third immunisation (Day 56 and 84 respectively) were stimulated with 6 µg of *Pf*AMA1 vaccine. Cell proliferation was measured by adding ^3^H thymidine and calculated relative to control wells. Box plots and whiskers show the range and the 25^th^, 50^th^ and 75^th^ percentile. Circles represent outliers. Measurements were performed at baseline, 28 days after the second immunisation and 28 days after the third immunisation (day 56 and 84 respectively).

All groups showed Peripheral Blood Mononuclear Cell proliferation upon stimulation with *Pf*AMA1 (fig. 5). Stimulation indices between PBMC's stimulated with 30, 6 or 1.2 µg/ml *Pf*AMA1 were similar. All groups of volunteers showed significant increase in proliferation upon stimulation with *Pf*AMA1 after the second immunisation. After the third immunisation none of the groups showed a further increase in stimulation index, rather the 10 µg Montanide group showed a significant decrease after the third immunisation (p = 0.03). There were no significant differences in stimulation index between the different adjuvant or dose groups.

### *In vitro* parasite growth inhibition

**Figure 6:**
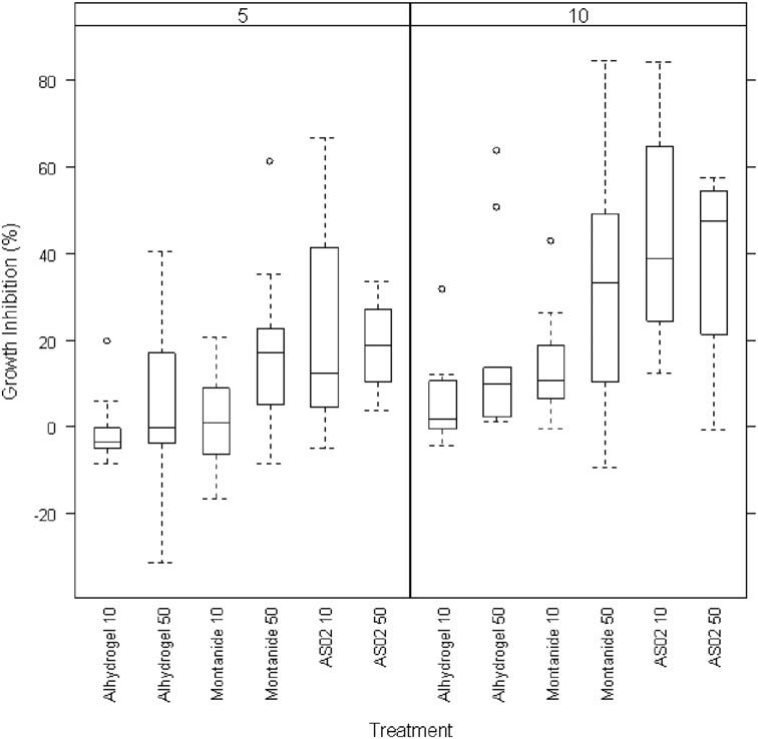
Percentage of growth inhibition of FVO-strain *P. falciparum* parasites after addition of 5 or 10 mg/ml IgG. Serum samples from volunteers immunised with *Pf*AMA1 were obtained four weeks after the final immunisation by per protocol analysis and included in a merozoite growth inhibition assay. Growth inhibition is expressed as a percentage to control.Boxes show 25^th^, 50^th^ and 75^th^ percentile growth inhibition, whiskers show the range, circles are outliers.

To estimate functionality of the induced antibodies, an *in vitro* GIA was performed. Results are shown as percentage inhibition compared to pre-vaccination sera from the same individual (fig. 6). At a concentration of 10 mg/ml, the median growth inhibition in the Montanide 50 µg and AS02 groups was about 30% and 50% respectively. In the Alhydrogel™ and Montanide 10 µg groups median inhibition was lower, ranging from 4 to 17%. Only differences between Alhydrogel™ and AS02 groups were significant (p = 0.002).

## Discussion

This trial demonstrates that reactogenicity of *Pf*AMA1-FVO[25-545] varies, depending on the adjuvant. Immunogenicity at both high and low doses and in all adjuvant formulations is good, although the type and magnitude of immune response varied among different adjuvant groups.

*Pf*AMA1-FVO[25-545] mixed with the adjuvants *Alhydrogel*™, Montanide and AS02 tended to be locally reactogenic, mainly causing short lasting injection site pain when administered to healthy adult volunteers. Most post immunisations adverse events were mild-to-moderate in intensity and have been seen previously with other vaccines[35]–[40]. Because this was the first time *Pichia Pastoris* produced FVO *Pf*AMA1 antigen was being given to humans, the occurrence of a grade 3 adverse event was a stopping criterium, which led to withdrawal of seven subjects post dose 2 for grade 3 (>50 mm) erythema. However, the erythema observed resolved spontaneously within three days of onset without any sequelae. The erythema is not considered a hindrance for further vaccination with the AS02 adjuvant. In terms of systemic adverse events, most were related to the AS02 adjuvant and transient resolving within two days with no sequelae. The pattern of transient, primarily mild to moderate systemic adverse events has been reported with another AMA-1 antigen adjuvanted with AS02 [41].

Three immunisations with 50 µg *Pf*AMA1 adjuvanted by Montanide induced a sterile abscess in two of ten volunteers. Progression of induration to a sterile abscess has been previously reported before after immunisation with Montanide [42]–[44]. In all reports the development of an abscess followed intramuscular immunisation and was accompanied by enhanced immunogenicity. The increased reactogenicity of Montanide-adjuvanted vaccines has been attributed to a combination of antigen dose and the formation of a vaccine depot that may persist locally and that is inherent to water-in-oil emulsions [45]. Similarly, induration at the previous immunisation site has been attributed to persistent antigen in previous trials [46]. A less condensed vaccination regimen and avoidance of the same injection sites may be measures to avoid induration.

To date, there are four other reports on clinical phase Ia trials of a *Pf*AMA1 vaccine. These trials employed different *Pf*AMA1 constructs and utilized different adjuvants. The constructs were of *P. pastoris* or *E. coli* origin or used a virally vectored delivery system [47]–[50].

The *P. pastoris*-produced *Pf*AMA1 comprised recombinant proteins based on sequences from the ectodomains of FVO and 3D7 strain AMA1 adjuvanted with *Alhydrogel*™ have been tested both in a phase Ia and Ib trial. As with previous studies in which malarial antigens have been adjuvanted with *Alhydrogel*™, this candidate vaccine showed an acceptable reactogenicity profile but a limited immune response. The Malkin et al. phase Ia trial shows a GIA response in only 4 of 22 subjects despite high seroconversion rates [51], similar to the data obtained here with *Alhydrogel*™. Interestingly, in our study, the *P. pastoris Pf*AMA1 combined with *Alhydrogel*™ was much less reactogenic and did not produce any erythema or induration, even though the doses were comparable. The lower Alhydrogel dose (500 µg per immunisation) used in this trial, as compared to Malkin et al. (800 µg) may also play a role in its decreased reactogenicity.

There are two trials utilizing the *E. coli*-produced 3D7 strain AMA1, one reconstituted in Montanide and a second formulated in AS02. The AMA1-Montanide combination was considered safe but the trial was compromised by apparent loss of potency [52]. The AMA1-AS02 combination [53] showed comparable local and systemic reactogenicity. Although Polhemus et al. were able to show recognition of the native antigen by IFA, growth inhibition results were approximately two fold lower than those found in this study.

Lastly, *Pf*AMA1 has also been evaluated in a multi-antigen malaria vaccine delivered in an attenuated vaccinia virus. Although weak protective effects were found, immunogenicity in that trial was poor [54].

After one year follow-up, we found antibody levels still to be significantly higher than baseline for all groups. This is in sharp contrast to the results of Malkin et al. [55] who reported detectable antibodies in only 50–90% of volunteers by day 364, even though they had been boosted much later (on Day 180). This suggests that a more condensed immunisation regime may affect the persistence of antibodies.

In this trial we have shown that the combination of clinical grade *Pf*AMA1 FVO [25-545] *P. pastoris* expressed material with either Montanide or AS02 is significantly more immunogenic than previous *Pf*AMA1 formulations, being capable of inducing high levels of antibodies for both dosages in both adjuvant groups. A positive trend between antigen dose, antibody response and *in vitro* parasite growth inhibition could be detected, although the effect of antigen dose on immunogenicity was negligible compared to the effect of varying the adjuvant. The wide variety of immune responses found in different adjuvant formulations stresses the importance of adjuvants as a critical component in malaria vaccine development.

The functionality of vaccine induced antibodies was assessed by growth inhibition assay. Although this assay has not been validated as a correlate of protection, this trial demonstrates that the standardised assay is able to demonstrate recognition of the native protein and thus functionality in vitro.

Different adjuvants are known to prompt immune responses towards Th1 or Th2. It has been previously reported that AS02 induces an immune response skewed towards Th1 [56], with production of primarily IFNγ. In contrast *Alhydrogel*™ is known to be Th2 inducer [57]. In this study, ratio's of cytokine production at day 84 showed relatively more IFNγ over IL-5 production in the Montanide and AS02 groups suggesting a pro-Th1 response, although statistically non-significant. Interestingly, the additional third immunisation generally did not lead to a further increase in IFNγ or IL-5 production or in lymphocyte proliferation. Rather, many volunteers showed a reduction in the response after the third immunisation. This difference could not be explained by inter-test variability. It remains to be investigated if it indicates a shift in the relative balance between immediate effector cells and long-lived memory cells.

Although this phase I trial is limited with respect to the size and generalizibility to the target population, it met its objectives to outline a generalizable safety profile. Specifically the direct comparison of the safety profile of different adjuvants is valuable for future development of AMA1 and other malaria vaccines. Furthermore, the malaria vaccine candidate AMA1 provides the possibility of assessing functionality of the immune response by a parasite growth inhibition assay. However, it must be noted that the growth inhibition assay is not validated as a correlate of protection, and is as such a limited predictor for efficacy.

With this study we have shown that the *Pf*AMA1 vaccine combined with different adjuvants, *Alhydrogel*™, Montanide and AS02 provided distinct reactogenicity profiles. All vaccine formulation were immunogenic at both dosages. Growth inhibition results indicate that induction of functional immune responses is probably dependent on adjuvant, underscoring the need for strong immunopotentiators for malaria vaccines. Altogether, these results are promising for a future development of a *Pf*AMA1 malaria vaccine.

## Supporting Information

Checklist S1 CONSORT Checklist
(0.06 MB DOC)

Protocol S1 Trial Protocol
(0.70 MB PDF)

Amendment S1 (0.26 MB PDF)

Amendment S2 (0.42 MB PDF)

Amendment S3 (0.30 MB PDF)

Amendment S4 (0.25 MB PDF)

Amendment S5 (0.52 MB PDF)

Amendment S6 (1.06 MB PDF)
