# Supplementary material for: Safety and Immunogenicity of a Recombinant Plasmodium falciparum AMA1 Malaria Vaccine Adjuvanted with Alhydrogel™, Montanide ISA 720 or AS02
Source: PLoS One. 2008 Dec 18;3(12):e3960. doi: 10.1371/journal.pone.0003960 (PMC2602972; doi:10.1371/journal.pone.0003960)
Supplement: Protocol S1 — Trial Protocol (0.70 MB PDF) [file pone.0003960.s002.pdf]

CLINICAL TRIAL PROTOCOL

Assessment of the Safety and Immunogenicity of three Formulations of the Recombinant *Pichia pastoris* Apical Membrane Antigen 1 (PfAMA-1-FVO[25-45]), Blood-stage Malaria Vaccine in Healthy Dutch Adult Volunteers : a Phase 1, Single-Blind, Randomised, Dose-escalating, Unicentre trial.

Trial identifier : **AMA-1_1_03**
EUDRACT Number: 2005-000232-24

Product: **PfAMA-1-FVO[25-545]**
Form: Lyophilised adjuvanted with Alhydrogel or Montanide ISA 720 or AS02A
Route: intra-muscular
Dosage: 10 and 50 µg

Trial Center:
UMC St Radboud
Geert Grooteplein 18
6500 HB Nijmegen
The Netherlands

Principal Investigator :
Prof. Robert Sauerwein, MD
Tel : +31243610577 Fax :+31243614666
e-mail: R.Sauerwein@mmb.umcn.nl

Investigators :
André van der Ven, MD
Tel : +31243614664 Fax: +31243614666
e-mail: A.vanderVen@AIG.umcn.nl

Co-investigator:
Meta Roestenberg, MD
Tel : +31243619515 Fax :+31243614666
e-mail: M.Roestenberg@ncmls.ru.nl

Biological Evaluators :
Rob Hermsen, PhD (UMC St Radboud)
Tel : +31243613663 Fax :+31243614666
Email : R.Hermsen@ncmls.kun.nl

Ed Remarque, PhD (BPRC)
Tel: 31152842798 Fax: +31152843986
Email: remarque@bprc.nl

Inventors:
Alan Thomas, PhD (BPRC)
Tel: +31152842538 Fax: +31152843986
Email: thomas@bprc.nl
Clemens Kocken, PhD (BPRC)
Tel: + 311528428 48 Fax: +31152843986
Email: kocken@bprc.nl

Information contained in this publication is the property of EURPEAN MALARIA VACCINE INITIATIVE (EMVI) and is confidential and will not be disclosed to third parties without written authorization from EMVI.No part of this document may be reproduced, stored in a retrieval system or transmitted in any form or by any means -electronic, mechanical, recording or otherwise - without the prior authorization of EMVI.

Sponsor:
European Malaria Vaccine Initiative
CIH, Bergen University, Norway
Director of Clinical and Regulatory Affairs :
Odile Leroy ,MD
13 rue des 4 Vents
92380 Garches, France
Tel: +33 1 47951781
Mob: +33 6 86783149
e-mail: odile.leroy@wanadoo.fr

Monitor:
Hildur Ella Blythman, MD
EMVI French Office
2 allée Alfred Sisley
78160 Marly le Roi
France
Tel: +33 1 39 58 43 65
Mobile: +33 6 08 12 52 68
hildur.blythman@wanadoo.fr
www.emvi.org

PfAMA-1-FVO[25-545]
Manufacturer:
Eurogentec SA
Parc Scientifique du Sart Tilman,
B-4102 Seraing, Belgium
Biologics Business Unit Manager
Michel Thiry, PhD
Tel: + 32 4 366 61 16 Fax: + 32 4 365 16 04
e-mail: m.thiry@eurogentec.com
Project Manager
Florence Xhonneux
Email: fl.xhonneux@eurogentec.com

AS02 adjuvant
Manufacturer:
GSK Biologicals
AS02A is the proprietary adjuvant system of GSK Biologicals SA

Version : Final_2
Date : 16/05/2005

Information contained in this publication is the property of EURPEAN MALARIA VACCINE INITIATIVE (EMVI) and is confidential and will not be disclosed to third parties without written authorization from EMVI.No part of this document may be reproduced, stored in a retrieval system or transmitted in any form or by any means -electronic, mechanical, recording or otherwise - without the prior authorization of EMVI.

Table of Contents

1 Background Information 2

1.1 Introduction 2
1.2 Epidemiology 2
1.3 Prevention and Control of Infection among Humans 3
1.4 Name and description of the investigational product 3
1.5 Summary of findings from non-clinical studies 5
1.5.1 Antigenicity: 5
1.5.2 Immunogenicity: 5
1.5.3 Safety and immunogenicity of Pf11-0 in rhesus macaques. 6
1.6 Summary of previous clinical trials 8
1.7 Rationale 9
1.7.1 Risks and benefit to human subjects 9
1.7.2 Rationale for adjuvant selection 9
1.7.3 Rationale for dosage selection: 9

2 Trial Objectives 10

2.1 Primary Objective 10
2.2 Secondary Objectives 10
2.2.1 Humoral Immune Response 10
2.2.2 Cellular immune response 10
2.3 Exploratory Objective(s) 10

3 Trial design and methodology 10

3.1 Trial design 11
3.1.1 Description and Justification of the Trial Design 11
3.1.2 Randomisation and Vaccine Allocation Procedure 11
3.1.3 Blinding and Code Breaking Procedure 11
3.2 Trial Plan 12
3.2.1 Trial Calendar/Timelines 12
3.2.2 Vaccination, Clinical Follow-up and Serology schedule 13
3.2.3 Trial Centres 13
3.2.4 Conduct of the Trial 14
3.3 Case Report Forms and Data Collection 14
3.3.1 Source Data 14
3.3.2 Diary 14
3.3.3 Case Report Forms 14
3.4 Procedures for Obtaining, Handling and Shipment of Biological Samples 15
3.4.1 Trial Laboratories 15
3.4.2 Obtaining Serum Samples and blood cells 15
3.4.3 Handling Serum Samples 15

4 Selection and Withdrawal of Subjects 16

4.1 Inclusion Criteria 16
4.2 Non-Inclusion Criteria 16
4.3 Conditions for Withdrawal from further Vaccination 16
4.4 Conditions for Withdrawal from the Trial 17

4.5 Termination Classification ... 17

5 Vaccines ... 18

5.1 Investigational Vaccine Characteristics ... 18
5.1.1 Description ... 18
5.1.2 Formulation ... 18
5.1.3 Precaution for use ... 19
5.2 Vaccination ... 19
5.3 Prior and Concomitant Therapy ... 20
5.4 Management of Vaccines ... 20
5.4.1 Labelling and Packaging ... 20
5.4.2 Storage and Shipment Conditions ... 20
5.4.3 Accountability ... 21
5.4.4 Return of Unused Products ... 21

6 Adverse event Management and reporting ... 21

6.1 Definitions ... 21
6.1.1 Adverse Event (or Adverse Experience) ... 22
6.1.2 Serious Adverse Event ... 22
6.1.3 Clinical laboratory parameters and other abnormal assessments qualifying as adverse events and serious adverse events ... 23
6.2 Safety Monitoring Plan ... 23
6.2.1 Role of Local Safety Monitor ... 23
6.2.2 Holding rules ... 24
6.2.3 Review of Safety Data by the Safety Monitor ... 24
6.2.4 Process for restarting vaccination ... 24
6.2.5 Process for stopping vaccination of a group or of the trial ... 25
6.3 Safety Data Collection and Management Procedures ... 25
6.3.1 Expected Adverse Vaccine Reactions: ... 25
6.3.2 Safety Data Collection ... 25
6.3.3 Time period, frequency, and method of detecting adverse events and serious adverse events ... 26
6.3.4 Assessment of causality ... 27
6.3.5 Medically attended visits ... 27
6.4 Follow-up of adverse events and serious adverse events and assessment of outcome ... 28
6.5 Reporting of Serious Adverse Events ... 28
6.5.1 Time frames for reporting of SAEs ... 28
6.5.2 Completion and transmission of serious adverse event reports ... 29
6.5.3 Regulatory reporting requirements for serious adverse events ... 30
6.5.4 Post study adverse events and serious adverse events ... 31
6.6 Pregnancy ... 31
6.7 Treatment of adverse events ... 32
6.8 Loss to follow-up Procedures ... 32

7 Evaluation Criteria ... 32

7.1 Primary Evaluation Criterion : Safety ... 32
7.1.1 Definition of the Criterion ... 32
7.1.2 Parameters to be measured ... 32
7.1.3 Method and Timing of Measurement ... 33
7.2 Secondary Evaluation Criteria : Immunogenicity ... 34
7.2.1 Humoral Immune response ... 34
7.2.2 Cellular Immune response: ... 34
7.3 Exploratory Evaluation Criteria: ... 35
7.4 Statistical Methods and Data Analysis ... 36
7.4.1 Principal Objective ... 36

7.4.2 Determination of the Sample Size 36
7.4.3 Data Set to be analysed 36
7.4.4 Statistical Methods 37
7.4.5 Data Management 37

8 Ethical Consideration 37

8.1 Informed Consent 37
8.2 Subject Benefit and Risks 37
8.3 Ethical Review 38

9 Quality Control and Quality Assurance 38

9.1 Direct Access to Source Data/Documents 38
9.2 Personnel Involved in the Trial 38
9.3 Modification of the Protocol 38
9.4 Investigator Procedures 39
9.5 Monitoring 39
9.5.1 Set-up Visit 39
9.5.2 Follow-up Visits 39
9.5.3 Close-out Visit 39
9.6 Audits and Inspections 40
9.7 Archiving 40

10 Confidentiality and Publication Policy 40

10.1 Confidentiality 40
10.1.1 Confidentiality of Data 40
10.1.2 Confidentiality of Patients Records 40
10.2 Publications 40
10.2.1 Publication Policy 40
10.2.2 List of Publication and Authorship 41

11 Financing and Insurance 42

11.1 Financial contract 42
11.2 Adverse Events Compensation, Stipends for participation 42
11.3 Insurance 42

12 Bibliographical References 42

13 Signatures/Investigator's Agreement to the Protocol 44

14 Appendixes 45

Appendix 1 : Information Sheet and Informed Consent Form 45

Appendix 2 : Declaration of Helsinki 51

Appendix 3 : Severity Scales 55

Appendix 4 : Investigator's Responsibilities 57

Appendix 5 : Text of Regulatory approval 64

Appendix 6 : SAE Report Form 65

Appendix 7 : Case Report Form 66

Appendix 8 : Investigator SOPs 67

SYNOPSIS

**Sponsor legal name:**	European Malaria Vaccine Initiative
**Finished product**	AMA-1 malaria vaccine
**Active ingredient**	**PfAMA-1-FVO[25-545]**
**Trial Title:**	Recombinant *Pichia pastoris* Apical Membrane Antigen 1 (PfAMA-1-FVO[25-545]), Blood-stage Malaria Vaccine in Healthy Dutch Adult Volunteers : a Phase 1, Single-Blind, Randomised, Dose-escalating, Unicentre trial.
**Trial Identifier:**	**AMA-1_1_03**
**Clinical phase**	Phase 1
**Principal Investigator**	Prof. Dr. Robert Sauerwein (UMC St Radboud)<br>UMC St Radboud
**Investigators**	Dr André van der Ven (UMC St Radboud)<br>Dr Meta Roestenberg (UMC St Radboud)
**Biological Evaluator**	Dr Rob Hermsen (UMC St Radboud)<br>Dr Ed Remarque (BPRC)
**Trial Centre(s)**	UMC St Radboud<br>Geert Grooteplein 18<br>6500 HB Nijmegen
**Planned Trial Period**	Approximately 01 jun 2005 to 01 sept 2006
**Primary Objective**	**To evaluate the safety** of 3 doses given at D0, D28 and D112 of 2 different dosages of AMA-1 (10 µg or 50 µg) adjuvanted either with alum hydroxide or AS02A or Montanide ISA 720 in healthy adults not previously exposed to the parasite *Plasmodium falciparum*
**Secondary Objective(s)**	**To assess the humoral response** to the vaccine antigen by measuring the level of IgG and its ability to recognise the native protein on merozoites.<br><br>**To assess the cellular immune response** by measuring the T cell proliferation and cytokine production following in vitro stimulation with the vaccine antigen.
**Exploratory Objectives**	**To assess the quality of the humoral immune response** by measuring<br><br>- IgG1, IgG2, IgG3, IgG4 subclasses<br>- The domain specificity of the response<br>- The broadness (allele specificity) of the response<br>- The ability to block parasite growth in vitro<br>- The ability to recognise the moab 4G2 binding site and recognition of 4G2 mimotopes<br>- IgG avidity
**Trial Design**	Phase 1, Single-Blind, Randomised, Dose-escalating, Unicentre trial.
**Planned Sample Size**	The total number of subjects will be 60.<br>6 groups of 10 subjects per group will be recruited
**Inclusion Criteria**	1. Age > 18 and < 45 years healthy volunteers (males or females).<br>2. General good health based on history and clinical examination.<br>3. All volunteers have to sign the informed consent form.

**Sponsor legal name:**	European Malaria Vaccine Initiative
**Finished product**	AMA-1 malaria vaccine
**Active ingredient**	**PfAMA-1-FVO[25-545]**
**Trial Title:**	Recombinant *Pichia pastoris* Apical Membrane Antigen 1 (PfAMA-1-FVO[25-545]), Blood-stage Malaria Vaccine in Healthy Dutch Adult Volunteers : a Phase 1, Single-Blind, Randomised, Dose-escalating, Unicentre trial.
	4. Negative pregnancy test.<br>5. Use of adequate contraception for females up to three months after the third injection (D140).<br>6. Reachable by phone during the whole study period (18 months).
**Non-Inclusion Criteria**	1. History of malaria or residence in malaria endemic areas within the past six months.<br>2. Positive serology for malaria antigen PfAMA-1<br>3. Previously participated in any malaria vaccine study<br>4. Symptoms, physical signs and laboratory values suggestive of systemic disorders, including renal, hepatic, cardiovascular, pulmonary, skin, immunodeficiency, psychiatric and other conditions, which could interfere with the interpretation of the study results or compromise the health of the volunteers.<br>5. Any laboratory abnormalities on screened blood samples beyond the normal range, as defined at UMC St Radboud. Positive HIV, HBV or HCV tests.<br>6. Volunteers should not be enrolled in any other clinical trial during the whole trial period.<br>7. Volunteers should not receive chronic medication, especially immunosuppressive agents (steroids, immunomodulating or immunosuppressive drugs) during the three months preceding the screening visit or during the study period.<br>8. Pregnant or lactating women.<br>9. Volunteers unable to give written informed consent.<br>10. Volunteers unable to be closely followed for social, geographic or psychological reasons.<br>11. Previous history of drug or alcohol abuse interfering with normal social function during a period of one year prior to enrolment in the study.<br>12. Volunteers should not perform exercise four hours before blood draw and should not donate blood for non study-related purposes during the entire duration of the study.<br>13. Known hypersensitivity to any of the vaccine components (adjuvant or peptide).<br>14. Volunteers are not allowed to receive any vaccination or gammaglobuline during a period three months prior to the first immunisation and up to six months after the 3rd immunisation. If a vaccination is necessary during this period, the volunteer will be withdrawn from the study.<br>15. Volunteers are not allowed to travel to malaria endemic countries during the study period.
**Investigational Product**	PfAMA-1-FVO[25-545]
Form	Lyophilised<br>Extemporaneously adjuvanted with either Alhydrogel, Montanide ISA 720 or AS02A, prior to vaccination
Dose	10µg and 50µg
Route	Intramuscular
Lot Number	To be added

**Sponsor legal name:**	European Malaria Vaccine Initiative
**Finished product**	AMA-1 malaria vaccine
**Active ingredient**	**PfAMA-1-FVO[25-545]**
**Trial Title:**	Recombinant *Pichia pastoris* Apical Membrane Antigen 1 (PfAMA-1-FVO[25-545]), Blood-stage Malaria Vaccine in Healthy Dutch Adult Volunteers : a Phase 1, Single-Blind, Randomised, Dose-escalating, Unicentre trial.
**Control Product**	None
**Vaccination Schedule**	Primary immunisation : day 0, 28<br>Boost : on day 112
**Follow-up duration**	One year after the first immunisation
**Serology Schedule**	D-28, D0, D28, D56, D112, D140 and D365
**Primary Evaluation Criteria**	The safety profile will be assessed on the following criteria<br>• Immediate reactogenicity; defined as any systemic adverse reactions occurring within 30 minutes after each injection,<br>• Local and systemic reactogenicity measured from day 0 to day 14 after each dose<br>• Any unsolicited adverse event resulting in a visit to a physician between each injection and one month after the third dose.<br>• Any Serious Adverse Event (SAE) occurring from the inclusion through out the study. The relationship of the adverse event to the study vaccine will be established by the investigator, using the following definitions : related or not related.<br>• Biological safety, one month after each vaccination, in reference with the baseline before the first dose, by measuring the following :<br>– RBC, hemoglobin, hematocrit, MCV, MCH, MCHC, platelets, WBC with differential counts<br>– Potassium, sodium, ASAT, ALAT, total bilirubin, alkaline phosphatase, γGT, creatinin
**Secondary Evaluation Criteria**	**The humoral response** to the vaccine antigen will be assessed by measuring the level of IgG by ELISA.<br>An IFA for at least two parasite strains will be employed to verify that the antibodies elicited by the vaccine recognise the native protein on merozoites.<br>•<br>**The cellular immune response** will be assessed by measuring the T cell proliferation and cytokine production following in vitro stimulation with the vaccine antigen.<br>T-cell proliferation will be performed with a standard proliferation assay employing tritium thymidin.<br>Cytokine production will be assayed by ELISPOT for the cytokines IL-4 and IFNγ to the vaccine antigens.<br>These measurements will be performed on samples obtained one month before and one month after the second and the third vaccinations (at D0, D56, and D140).
**Exploratory Evaluation Criteria**	**The quality of the humoral immune response** will be assessed by measuring<br>• IgG1, IgG2, IgG3, IgG4 subclasses by ELISA on samples obtained at days 0, 56 and 140<br>• IgG responses to FVO AMA-1 domains by ELISA on samples obtained at days 0, 56, 140 and 365<br>• IgG responses to allelic variants by ELISA on samples obtained at days 0, 28, 56, 112, 140 and 365<br>• The ability to block parasite growth in vitro by a a Plasmodium inhibition assay (PIA) against 3 parasite strains on samples obtained at days 0, 56, 140 and 365

**Sponsor legal name:**	European Malaria Vaccine Initiative
**Finished product**	AMA-1 malaria vaccine
**Active ingredient**	**PfAMA-1-FVO[25-545]**
**Trial Title:**	Recombinant *Pichia pastoris* Apical Membrane Antigen 1 (PfAMA-1-FVO[25-545]), Blood-stage Malaria Vaccine in Healthy Dutch Adult Volunteers : a Phase 1, Single-Blind, Randomised, Dose-escalating, Unicentre trial.
	• The ability to recognise the moab 4G2 binding site by a competition ELISA on samples obtained at days 0, 56, 140 and 365<br>• The ability to recognise 4G2 mimotope peptides by ELISA on samples obtained at days 0, 56, 140 and 365<br>IgG avidity by Biacore assessment on samples obtained at days 0, 56 and 140
**Statistical Methods**	The analysis shall be descriptive; the sample size does not allow any comparison between groups.

Flow Chart

Prime & Boost phase, 19 Visits, 3 Vaccinations, Randomised, Dose Escalating, 365 Days duration/subject

**Visit Number**	**V1**	**V2**	**V3**	**V4**	**V5**	**V6**	**V7**	**V8**	**V9**	**V10**	**V11**	**V12**	**V13**	**V14**	**V15**	**V16**	**V17**	**V18**	**V19**
**Trial Timelines (Days,)**	**D-28**	**D0**	**D1**	**D3**	**D7**	**D14**	**D28**	**D29**	**D31**	**D35**	**D42**	**D56**	**D112**	**D113**	**D115**	**D119**	**D126**	**D140**	**D365**
**Time Windows (Days or Hours)**		± 7 D	± 6 hrs	± 24 hrs	± 24 hrs	± 2 D	± 2 D	± 6 hrs	± 24hrs	± 24 hrs	± 2 D	± 2 D	±7 D	± 6 hrs	± 24hrs	± 24 hrs	± 2 D	± 2D	± 14 D
**Visit Intervals**		V1 +4 wks	V2 +24 hrs	V2 + 3D	V2 + 7D	V2 +2 wks	V4 + 2 wks	V7 + 24hrs	V7 + 3D	V7 + 7D	V7 +2 wks	V11 + 2 wks	V7 +12wks	V13 + 24hrs	V13 + 3D	V13 + 7D	V9 +2 wks	V11 +2 wks	V1 +1 year
**Vaccination Doses**		**Vac1**					**Vac2**						**Vac3**						
**Eligibility Criteria**	X																		
**HIV, HCV, HBV tests**	X																		
**In- & Non-Inclusion Criteria**		X																	
**Informed Consent**	X	X																	
**Medical History**	X																		
**Physical Examination**	X	X	X	X	X	X	X	X	X	X	X	X	X	X	X	X	X	X	X
**Contra- Indications Review**		X					X						X					X	
**Pregnancy Test**	X	X					X						X						
**Immediate surveillance (30 min)**		X					X						X						
**Solicited Events**			X	X	X	X		X	X	X	X	X		X	X	X	X		
**Unsolicited Events**		X	X	X	X	X		X	X	X	X	X		X	X	X	X		
**Serious adverse events**	To be reported at any time during the trial																		
**Prior and concomitant therapy**		X					X						X						
**Blood Sampling**	BS1	BS2			BS3		BS4			BS5		BS6	BS7			BS8		BS9	BS10
**AMA-1 IgG, IgG isotypes, IgM**		X					X					X	X					X	X
**GIA; t-cell proliferation**		X										X						X	
**IFNγ, ELISPOT**		X											X					X	X
**Trial Phase**	Primary Immunisation												Booster Immunisation						
**Termination record / interim**												X						X	
**Termination record / Final**																			X
**Diary card provided**		DC1					DC2						DC3						
**Diary card collected**						DC1					DC2					DC3			
**Case Report Form Sections**	CRFA						CRFB						CRFC						CRFD

ABBREVIATIONS AND SYMBOLS USED

95% CI	95% Confidence Interval
ALAT	Alanine-Amino-Transferase
ASAT	Asparagine-Amino-Transferase
BPRC	The Biomedical Primate Research Center
CRF	Case Report Form
Dn	Day n
EC	Ethics Committee
ELISA	Enzyme-linked Immunoassay
ELISPOT	Enzyme Linked ImmunoSpot
EMVI	European Malaria Vaccine Initiative
FVO	Vietnam Oak Knoll
GCP	Good Clinical Practice
PIA	Plasmodium Inhibition Assay
γGT	γ-Glutamyl-Transpeptidase
GMT	Geometric Mean Titer
HBV	Hepatitis B Virus
HCV	Hepatitis C Virus
HIV	Human Immunodeficiency Virus
IFA	Immuno-Fluorescence Assay
IFNγ	Interferon γ
IgG	Immunoglobin G
IL-4	Interleukine-4
INN	International Non-proprietary Name
IRB	Institutional Review Board
IRAEF	Initial Report for Adverse Event Form
MCH	Mean Corpucular Haemoglobin
MCHC	Mean Corpuscular Haemoglobin Concentration
MCV	Mean Corpuscular Volume
MW	Molecular Weight
NIMR	National Institute of Medical Research - London
Pf	Plasmodium falciparum
RBC	Red Blood Cell Count
SAE	Serious Adverse Event
UMCN	University Medical Centre Nijmegen
WBC	White Blood Cells
WHO	World Health Organization

1 Background Information

1.1 Introduction

Malaria is a common and serious tropical disease. It is a protozoal infection transmitted to human beings by mosquitoes biting mainly between sunset and sunrise. Human malaria is caused by four species of Plasmodium protozoa: *Plasmodium falciparum, P. vivax, P. ovale* and *P. malariae.* Malaria is a public health problem in over 90 countries worldwide, inhabited by some 40% of the world population, i.e. over 2 billion people. It has been estimated that the incidence of malaria in the world may be in the order of 300 million clinical cases each year. Countries in tropical Africa account for more than 90% of these cases. Malaria mortality is estimated at more than 1 million deaths worldwide per year. Most malaria deaths occur among young children in Africa, especially in remote rural areas with poor access to health services. Other high-risk groups include women during pregnancy, and non- immune travellers, refugees, displaced persons, or labour forces entering into endemic areas. Malaria infection has been increasing over recent years due to a combination of factors including increasing resistance of malarial parasites to chemotherapy and increasing insecticide resistance of the Anopheles mosquito vector, ecological and climate changes, increased international travel to malaria-endemic areas.

Parasites (sporozoite stage) are injected into the skin capillaries by a female Anopheles sp. mosquito. From there they travel via the bloodstream to the liver, where they develop and multiply in liver cells before entering the blood stream again (merozoite stage) and invading for further reproduction. From there other internal organs, e.g. the brain, can be affected as clumps of infected erythrocytes start blocking capillary blood flow.

1.2 Epidemiology

Malaria affects 40% of the world's population with more than 1 million deaths annually. This represents a tremendous human suffering and a burden that prevents the development of the affected endemic communities. Malaria is one of the world's greatest public health problems particularly affecting young children and pregnant women in the poorest areas of sub-Saharan Africa.

The increasing emergence of insecticide resistant vectors and drug resistant parasites calls for investment in new and better control tools. Malaria vaccines hold the potential to dramatically alleviate the burden of malaria. However, our understanding of the mechanisms underlying protective immunity is incomplete and specific markers of protection still needs to be defined.

Clinical malaria is caused by the cyclical proliferation of the asexual stages of Plasmodium falciparum in red blood cells. Malaria mortality is primarily due to organ dysfunction in particular of the brain, following sequestration in the micro-vasculature by infected red blood cells.

An effective malaria vaccine will require the induction of appropriate humoral and cellular immune responses, against several key parasite antigens expressed during the various stages of the parasite life cycle. Each stage in the life cycle provides an opportunity for a vaccine.

It is a well-established observation that repeated exposure to malaria parasites can lead to the development of solid clinical immunity. Clinically immune individuals generally have a lower parasite density and the immunity is effective at reducing mortality. An a-sexual stage blood stage vaccine will induce immunity that controls parasitemia and therefore prevent clinical complications increasing the risk of death.

1.3 Prevention and Control of Infection among Humans

There are several reasons why malaria remains to be one of greatest health problems. One of the main reasons is failure to achieve adequate coverage with, and reduced pricing of, existing tools such as drugs and insecticide-treated bednets. There is user ignorance of how to use these tools. The decreasing effectiveness of existing tools (e.g. emergence of drug resistance, particularly to chloroquine and sulphadoxine-pyrimethamine, and, in south-east Asia, resistance to third-and fourth-line antimalarials; resistance to insecticides, including pyrethroids) is a major challenge. The availability of an efficacious malaria vaccine would certainly be a major achievement to overcome the failures of the current control strategy.

1.4 Name and description of the investigational product

Apical membrane antigen 1 (AMA-1) is considered one of the leading candidates for inclusion in a vaccine against blood stages of Plasmodium falciparum. AMA-1 is a Type I integral membrane merozoite protein that is implicated in the process of invasion of host erythrocytes.

AMA-1 of *P. falciparum* (*Pf*AMA-1) the product of a single, essential gene, is initially localised on the apical invasion organelles of the merozoite. It subsequently undergoes proteolytic processing and relocalisation to the merozoite external membrane. AMA-1 has structural homologues in all species of malaria studied to date as well as in *Toxoplasma gondii.* Important determinants of protein structure, such as the position of cysteine residues, are well-conserved between species.

*Pf*AMA-1 has an apparent molecular weight on SDS-PAGE of 83kDa, and comprises 622 amino acids containing 16 cysteine residues. Based on disulphide bonding a domain structure has been assigned to the molecule (see Figure 1).

Figure 1 : Disulphide bonding in *P. falciparum* AMA-1 defines the pro-sequence and domains I, II and III. ***SP (signal peptide), TM (transmebrane region)***

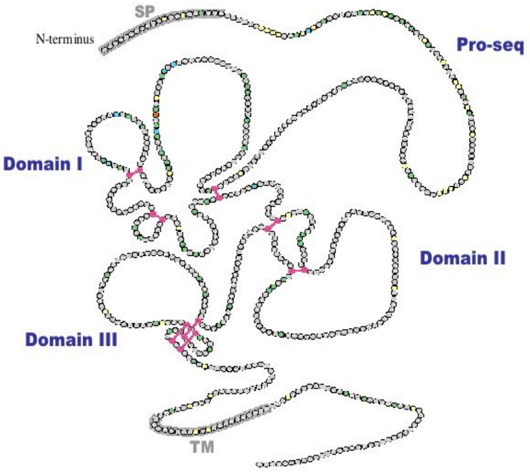

AMA-1 is a malaria vaccine candidate for the following reasons:

AMA-1 appears to be immunogenic in populations naturally exposed to malaria infection

Antibodies against AMA-1 (induced by immunisation with AMA-1 or purified from the serum of naturally exposed subjects) block merozoite invasion of red blood cells *in vitro*

Vaccination with either AMA-1 purified from merozoites, or with AMA-1 produced by recombinant expression, has protected mice and primates from malaria

Passive transfer of antibodies against AMA-1 has protected mice against malaria

When AMA-1 undergoes chemical reduction, to destroy disulphide bonds, it loses the ability to induce functional antibodies. Eukaryotic secretion expression systems can provide recombinant proteins with the correct disulphide bonding without the need for protein refolding. Dr Thomas and colleagues therefore selected the yeast, *Pichia pastoris*, for AMA-1 production. *P. pastoris* has already been used by Dr Thomas to produce AMA-1 of *P. vivax*. This material induced antibodies that inhibited parasite invasion and was immunogenic in rhesus monkeys.

P. falciparum has a codon usage highly biased to A and T and, as a result, *P. pastoris* was not able to utilise the native *P. falciparum* AMA1 gene. In collaboration with colleagues at NIMR in London, UK, an alternative AMA-1 gene was designed and synthesised. This artificial gene is based on the codon usage of *P. pastoris*. The native AMA-1 protein, as found in merozoites, does not appear to be N-glycosylated. However, *P. vivax* AMA-1 expression using an unmodified gene resulted in heterologous product formation due to glycosylation. To obtain a homogenous product, the 6 potential N-glycosylation sites occurring in *P. falciparum* AMA-1 were substituted with amino acids naturally occurring in AMA-1's from other strains of *P. falciparum* or other *Plasmodium* species.

Using this artificial gene, large amounts of AMA-1 have been produced at BPRC from series of recombinant yeast. Because it is not known which domains of AMA-1 mediate the induction of parasite inhibitory Ab, efforts have focused on expression of the AMA-1 ectodomain comprising pro-sequence (without the signal peptide) and domains I, II and III, with a stop just N-terminal of the transmembrane region. A construct, *Pf*4mH (comprising *Pf*AMA-1 amino acids 25-544, a myc epitope and a Hexa-His epitope) was prepared to provide a readily purified protein suitable for initial analyses.

Subsequently the construct *Pf*11.0 (comprising only *Pf*AMA1 amino acids 25-545) was prepared to provide material for clinical evaluation. Fermentation and purification of *Pf*11.0 have been developed to provide a process delivering a product with a highly purified active ingredient under GMP.

A large number of AMA-1 haplotypes are known to exist. The effects of this diversity on vaccine performance are unknown. The FVO strain was selected for expression based on its sequence diversity from the 3D7 strain that was being used by other researchers.

1.5 Summary of findings from non-clinical studies

1.5.1 Antigenicity:

Antigenicity of *Pf*4mH was evaluated by ELISA (Figure 2) using *Pf*AMA-1-specific mAbs 4G2 (recognising a conformational epitope in the ectodomain and capable of blocking parasite multiplication *in vitro*), 58F8 (recognising a linear epitope in the N-terminal region of *Pf*AMA-1), and 28G2 (recognising a linear epitope within the cytoplasmic C-terminal region of *Pf*AMA-1), and endemic human serum from Guinea-Bisau (antibodies in naturally exposed human subjects are predominantly directed towards conformational determinants of *Pf*AMA-1 that are dependant on di-sulphide bonding). As expected, mAb 28G2 and human serum from uninfected European donors are non-reactive with Pf4mH. mAb 58F8 displays strong reactivity. *Pf*4mH is well recognised by the conformational, parasite-inhibitory mAb 4G2 and by antibodies in human serum from a malaria endemic region, demonstrating an authentic antigenic profile.

Figure 2 : Antigenicity of Pichia-expressed *Pf*AMA-1

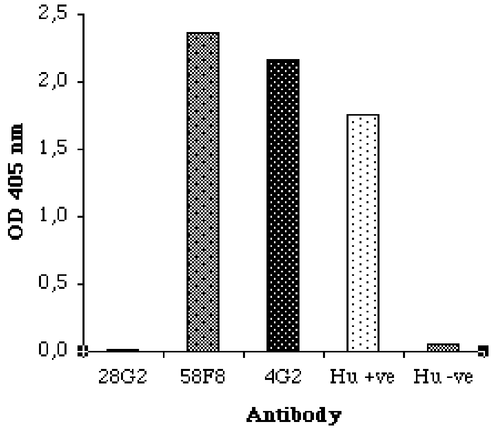

1.5.2 Immunogenicity:

Rabbit polyclonal antisera were prepared by injection of 100 µg purified *Pf*4mH (Rabbits 678, 679 and 680) using Freund's complete adjuvant, followed by 3 further injections of 100 µg at days 14, 28 and 56

using Freund's incomplete adjuvant. Antisera obtained 4 weeks after the last injection were tested for reactivity by ELISA and immunofluorescence (IFA).

Rabbit	ELISA titre[1]	IFA titre[2]	
	*Pf*4mH	FCR3	NF54
678	55765	2.5×10^5	1.3×10^5
679	27753	2.5×10^5	0.6×10^5
680	12663	1.3×10^5	0.3×10^5

1. Titres are represented as the reciprocal dilution at which an OD of 1.0 is obtained. For an approximate conversion this to end point titres, multiply by 40
2. End point titres

Antibodies from sera obtained 4 weeks after the final immunisation were purified on protein A columns using standard protocols, extensively dialysed against RPMI 1640, filter-sterilised and used in the *Plasmodium* inhibition assay (PIA) (Figure 3).

Figure 3: *Plasmodium* inhibition assay (PIA) :

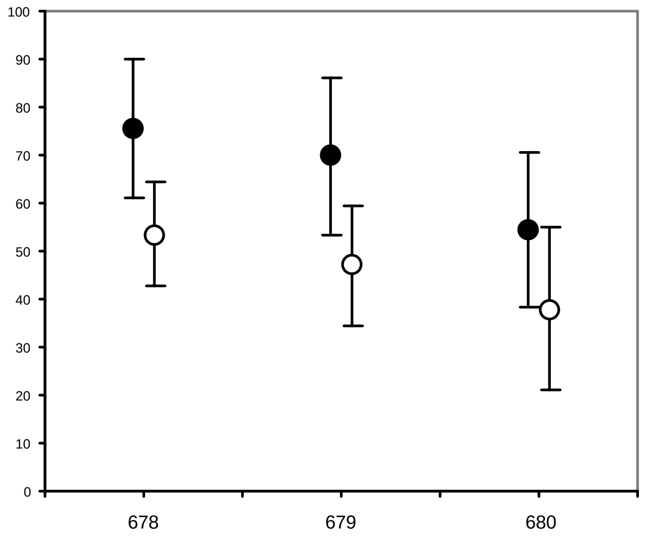

The effect of purified IgG on parasite invasion was evaluated using *in vitro* matured *P. falciparum* schizonts at a starting parasitemia of 0.5-1%, a haematocrit of 3.0% and a final total volume of 150 µl in standard culture medium. Radiolabel (hypoxanthine) was added after 17 hours of incubation. Parasites were harvested 10 hours later and incorporation of label was determined. Parasite growth inhibition, reported as a percentage, was determined as follows:

$100—((\text{average } CPM_{experimental}/\text{average } CPM_{control}) \times 100)$.

The incorporation for erythrocytes alone was subtracted from all averages prior to determining the percentage inhibition. Control IgG was isolated from rabbits that had been immunised according to the same scheme with adjuvant only. The average of five PIA's for Rb 678, 679 and 680 IgG at 1.5 (●) and 0.75 mg/mL (○) is shown.

1.5.3 Safety and immunogenicity of Pf11-0 in rhesus macaques.

1.5.3.1 Safety in rhesus macaques

The safety and tolerability of two dosages (10 and 50 µg) *Pf*11.0 produced under GLP conditions and formulated in three adjuvants (Alhydrogel, AS02A and Montanide ISA 720) was tested in 24 rhesus macaques divided over 6 groups. The monkeys were vaccinated on days 0, 28 and 84. Blood samples were taken on days 0, 1, 2, 3 and 14 following each vaccination.

No vaccine-related severe adverse events were observed. One monkey in the Montanide ISA 720 10 µg group had a large haematoma in the thigh following the second vaccination resulting in a hypovolemic shock. The monkey was subsequently treated with intravenous fluid (this also explains the low erythrocyte and haemoglobin values in one of the animals in this group) and recovery was uneventful. One monkey in the Alhydrogel 10 µg group developed a lump (enlarged lymph node) in the left thigh two weeks following the last vaccination. No significant changes in body weight were observed during the 98-day observation period. As is usual in vaccine studies, following vaccination transient elevations were observed in the white blood cell count, neutrophils, eosinophils, basophils, ALAT and ASAT. No persistent abnormalities in laboratory values were observed. As is usual for experiments in which blood is repeatedly sampled transient decreases were observed for haemoglobin and red blood cells. All changes in parameters returned to baseline within two weeks.

No long-term changes were observed for: basophils, erythrocytes, haemoglobin, creatinin, urea and creatinin kinase. Linear regression analysis revealed elevations over the 98-day course of the in: white blood cell count, lymphocytes, monocytes, eosinophils and ASAT. Decreases were observed for mean corpuscular volume and. At the end of the study (day 98), all animals had values within the reference range for the following parameters: neutrophils, lymphocytes, monocytes, basophils, haemoglobin, urea, creatinin and ALAT. For the following parameters some animals did not have values within the reference range at day 98: white blood cell count (1/24), eosinophils (5/24), erythrocytes (1/24), mean corpuscular volume (4/24) and asat (2/24).

1.5.3.2 Immunogenicity in rhesus macaques

Serum was obtained from the 24 macaques during the study and antibody responses were assessed. Figure 4 and 5 show that AMA-1 induces antibodies in rhesus macaques. Before vaccination all animals had titres close to the lower detection limit of the assay (0.03, 0.02 to 0.05 µg/mL mean 95% CI). Following vaccination titres increased significantly in all groups ($P < 0.001$). There were no differences in the amount of antibody elicited by the two dosages (Figure 4). The kinetics of antibody response was fastest in the Alhydrogel group where all monkeys responded well after the first vaccination. In contrast, the ISA and AS02A groups required two immunisations in order to observe responses > 10 µg/mL IgG in all monkeys. At day 14 and day 28, IgG titres were significantly lower in the AS02A group as compared to the Alhydrogel group (0.04 fold, 95% CI 0.01 - 0.13 and 0.02 fold, 95% CI 0.00 - 0.17, respectively). This observation was, although not statistically significant, also made at day 42, when titres in the AS02A group were about 0.52 fold lower 95% CI (0.25 - 1.06). At day 84 titres in the Alhydrogel and AS02A groups were similar, whereas titres in the Montanide ISA 720 group were 1.83 fold higher 95% CI (0.63 - 5.36). At day 98, the lowest titres were measured in the Alhydrogel group. Titres were higher in the AS02A and Montanide ISA 720 groups 2.04 fold (0.50 - 8.41) and 3.86 fold (0.94 - 15.88), respectively.

Before vaccination all animals were IFA-antibody negative (data not shown). Two weeks following the third vaccination all animals had developed IFA-antibodies (data not shown). As was observed for IgG levels, dose had no apparent effect on IFA titres. Adjuvant did, although not statistically significant, appear to influence IFA titres. The lowest titres were observed in the Alhydrogel group, followed by

the AS02A group. The highest IFA titres were observed in the Montanide ISA 720 group (data not shown).

Figure 4: The effect of antigen dose (10 or 50 μg) on IgG levels

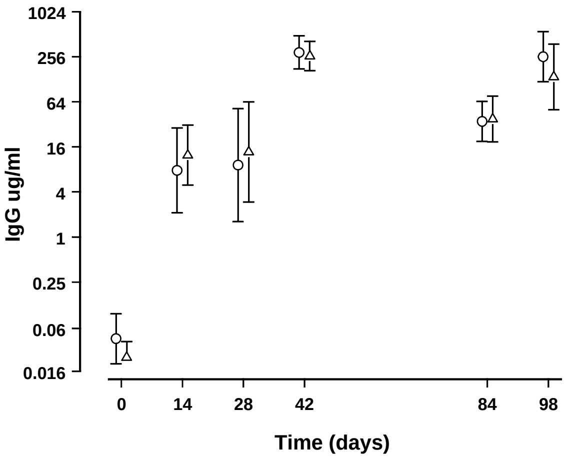

Legend:
○ (Circles) = 10 μg,
Δ (Triangles) = 50 μg,
I (Error bars) represent 95% CI

Figure 5: The effect of adjuvant on IgG Levels

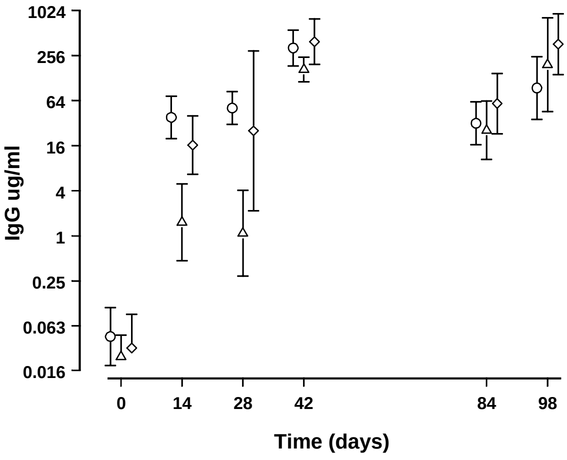

Legend:
○ (Circles) = Alhydrogel
Δ (Triangles) = AS02A
◊ (Diamonds) = Montanide ISA 720

I (Error bars) represent 95% CI

1.6 Summary of previous clinical trials

Three phase 1 clinical trials are ongoing, with different formulation of AMA-1 antigen. One trial is assessing *Escherichia coli* recombinant AMA-1 vaccine from the Walter Reed Army Institute of Research, United States. Two trials are evaluating a *P. pastoris*-expressed AMA-1. One of these comprises a chimera of MSP1-AMA-1. No published results of those trials are available.

1.7 Rationale

1.7.1 Risks and benefit to human subjects

The sterility of the product and the absence of toxicological effects are guaranteed by the results of the quality controls performed by Eurogentec and the pharmaco-toxicological study performed by NOTOX.

Safety of the vaccine in rhesus macaques has been tested and no vaccine-related severe adverse events were observed.

1.7.2 Rationale for adjuvant selection

i. Alum, is most widely used and generally accepted for use in human vaccines. Results in other malaria vaccine trials show that good immune responses can be obtained

ii. Montanide ISA 720 is approved for use in humans. Results in previous malaria vaccine trials have shown excellent immune responses with acceptable safety profiles;

iii. AS02A, which has been developed by GSK, is an oil/ water emulsion containing MPL® and QS21. Most of the data on AS02A adjuvant has been from clinical trial using the RTS,S antigen . The RTS,S antigen is a fusion protein composed of a circumsporozoite based antigen and hepatitis b surface antigen. The candidate RTS,S/AS02A malaria vaccine has been used in phase I/II clinical trials in malaria-naïve adults in Belgium and the USA and semi-immune (malaria-experienced) adults in The Gambia and Western Kenya. The vaccine has also been used in paediatric clinical trials in children aged 1-11 years in The Gambia and Mozambique.

   To date, at least 481 adult subjects have received over 1250 doses of the RTS,S/AS02A malaria vaccine. 60 children aged 6-11 years have received 179 doses of RTS,S/AS02A and 1825 children aged 1-5 years have received 5284 dose of RTS,S/AS02A .

   The reported adverse events following vaccination in children have been pain and swelling at the injection site, loss of appetite, upset stomach, fever, fussiness and tiredness. In generally these adverse events have been transient lasting generally for two days and mainly mild to moderate in intensity. In adults, the most frequently reported adverse events have been pain, myalgia and fatigue and transient resolving usually within 4 days. Severe adverse events were infrequent and resolved or decreased in intensity within 2 days.

   To date AS02A-containg vaccines did not elicit any serious adverse events that necessitated the withdrawal of the vaccine from clinical use...

1.7.3 Rationale for dosage selection:

Previous studies have shown that a dosage of 300 µg of malaria antigens (MSP-1, MSP-2 and RESA) in Montanide ISA 720 gave rise to local discomfort leading to transient limitations in activities of daily living (0). Studies with GLURP indicate that a 100 µg dosage either in Alum or Montanide ISA 720 frequently gave rise to local reactions (Sauerwein pers comm.). Given these considerations, it was decided to use a maximum dose of 50 µg. A dosage of about 50 µg of malaria antigens in Montanide ISA 720 appears to be immunogenic, whilst giving only mild to moderate (local) reactogenicity (0, 0).

2 TRIAL OBJECTIVES

2.1 Primary Objective

To evaluate the safety of 3 doses given at D0, D28 and D112 of 2 different dosages of AMA-1 (10 µg or 50 µg) adjuvanted either with Alhydrogel or AS02A or Montanide ISA 720 in healthy adults not previously exposed to the parasite *Plasmodium falciparum*. The safety and the tolerability of the vaccine will be assessed on the rate of solicited and unsolicited events/reactions The safety profile will include local and systemic reactions/events as well as the biological safety, based on a clinically significant change of the baseline value of the main biological criteria.

2.2 Secondary Objectives

2.2.1 Humoral Immune Response

To assess the humoral response to the vaccine antigens (PfAMA-1_{25-545}). The antibody response will be measured by ELISA and IFA.

-

2.2.2 Cellular immune response

To assess the cellular immune response T cell proliferation will be measured following in vitro stimulation with the vaccine antigen and parts thereof. Cytokine production will be assayed by ELISPOT for the cytokines IL-4 and IFNγ following in vitro stimulation with the vaccine antigen.

2.3 Exploratory Objective(s)

To assess the quality of the humoral immune response by measuring

- IgG1, IgG2, IgG3, IgG4 subclasses
- The domain specificity of the response
- The broadness (allele specificity of the response)
- The ability to block parasite growth in vitro
- The ability to recognise the moab 4G2 binding site and recognition of 4G2 mimotopes
- IgG avidity
-

3 TRIAL DESIGN AND METHODOLOGY

3.1 Trial design

3.1.1 Description and Justification of the Trial Design

The trial is a single-blind, randomised, dose escalating, unicentre trial. Three different adjuvants will be assessed, Alum, Montanide and AS02A. Two different dosages – 10µg and 50µg – of AMA-1 will be evaluated for each adjuvant. Six groups of 10 volunteers will be included. The groups are the following:

Group	Adjuvant	Dosage	Number of subjects
Al10	Alum	10µg	10
Al50	Alum	50µg	10
M10	Montanide ISA 720	10µg	10
M50	Montanide ISA 720	50µg	10
Ao10	AS02A	10µg	10
Ao50	AS02A	50µg	10

The clinical, biological and immunological data, when collected after immunization, will be mainly compared to the baseline of each subject. The trial is not designed to allow a comparison between groups. Thus, a formulation will be considered for future clinical trial only if the rate of adverse events/reactions deemed to be vaccine related, is found to be at the same range as routine vaccine in adults, i.e. events/reactions < 10% (The sample size used does not allow for 10%, but rather 25%). Furthermore, the dosage choice will be based on the level and the functionality of the immune response as well as the persistence of the immune response.

3.1.2 Randomisation and Vaccine Allocation Procedure

A total of 60 volunteers will be recruited for the study and allocated to six vaccination groups at random. The aim will be to recruit all 60 volunteers before the intended start of the study. If this is not achieved we will randomise in two blocks of 30 volunteers. The randomisation procedure will be performed in the following way :

When participants are found to be eligible and have signed their informed consent they will be numbered 1 to 60 (in order of enrolment) the treatments (vaccine formulations) will be randomly assigned to 6 groups of 10 numbers through a specific randomisation computer program. These groups will be the 6 treatment groups. We will not randomise for sex and/or age.

3.1.3 Blinding and Code Breaking Procedure

Vaccines will be administered in a single blind manner, i.e., only the vaccinator will know the vaccine adjuvant given to the subject. A research nurse will perform the vaccinations. Neither physicians, nor technicians will know the adjuvant.

Randomisation will be performed at EMVI. Randomisation list will be provided as scratchable lists (similar to lottery scratchboards). The randomisation lists will be made available to the Pharmacist in charge of the preparation of the vaccine and to the safety monitor in Nijmegen and one copy will be kept in the EMVI Executive Director office in Copenhagen. The original randomisation list will be kept by the statistician of the trial.

Code-breaking :

The code can be broken by one of the randomisation list holders or by the statistician only in case of serious adverse event needing urgent treatment depending on the vaccine and/or adjuvant the subject has received

The Safety Monitor, however, may break the code if it is absolutely necessary for the monitoring of the safety of the volunteers to unblind. This certainly applies if holding rules or stopping rules are being discussed. Please refer to section 6.2.2.

In case of pregnancy or SAE's, the safety monitor will break the code in order to inform GSK and EMVI about the adjuvant used. In this case, the investigator will remain blinded, unless unblinding is absolutely necessary.

The requirement should come from the investigator and /or the EMVI Clinical and regulatory Affairs Director after discussion with the Principal investigator, if possible.

If the code is broken it must be documented and reported. A code breaking form will allow the above users to report the code breaking request and code breaking result.

In case of significant difference in rate of severe reactions as defined in section 7.1.2, within the 3 different adjuvant groups, codes will be broken for the statistical analysis after the primary immunisation series of the 10µg dosage step, in order to decide or not the continuation of the vaccination with the higher dose.

The different vaccine formulations will be prepared in the NCMLS pharmacist under sterile conditions. Each syringe will be labelled/coded according to a randomisation document (key document). Vaccine labels will contain the code number (see section 5).

The vaccination will be carried out by a research nurse. This nurse will be the only one who knows which vaccination formulation is given to a certain volunteer. Neither volunteer nor the physicians who evaluate the reactions to the vaccinations will know the adjuvant. The physician will know the dose of AMA-1 in the vaccine formulation since this protocol dictates a step-up procedure starting with the 10 µg AMA-1 dose. The technicians who will perform the immunological assays will also be blinded to vaccine group.

3.2 Trial Plan

3.2.1 Trial Calendar/Timelines

Study protocol will be submitted to the Institutional Review Board of UMCN, and has to be approved before volunteers will be recruited. Written informed consent will be obtained from volunteers after free discussion with the Principal Investigator or Co-Investigator, following the procedure of Informed Consent (see Appendix 8). At least two weeks will be allowed before consent signature. Volunteers will be informed that they will not gain health benefits from this study and of the potential risks they may be exposed to.

In January 2005 we will submit the study protocol to the IRBN. We plan to start approximately June 2005 with the trial. For safety reasons we will start with the 10µgr AMA-1 formulations. Every week a group of 15 volunteers will start (see flow chart). Volunteers will be followed for one year. September 2006 the study will be finished. (see GANTT chart below)

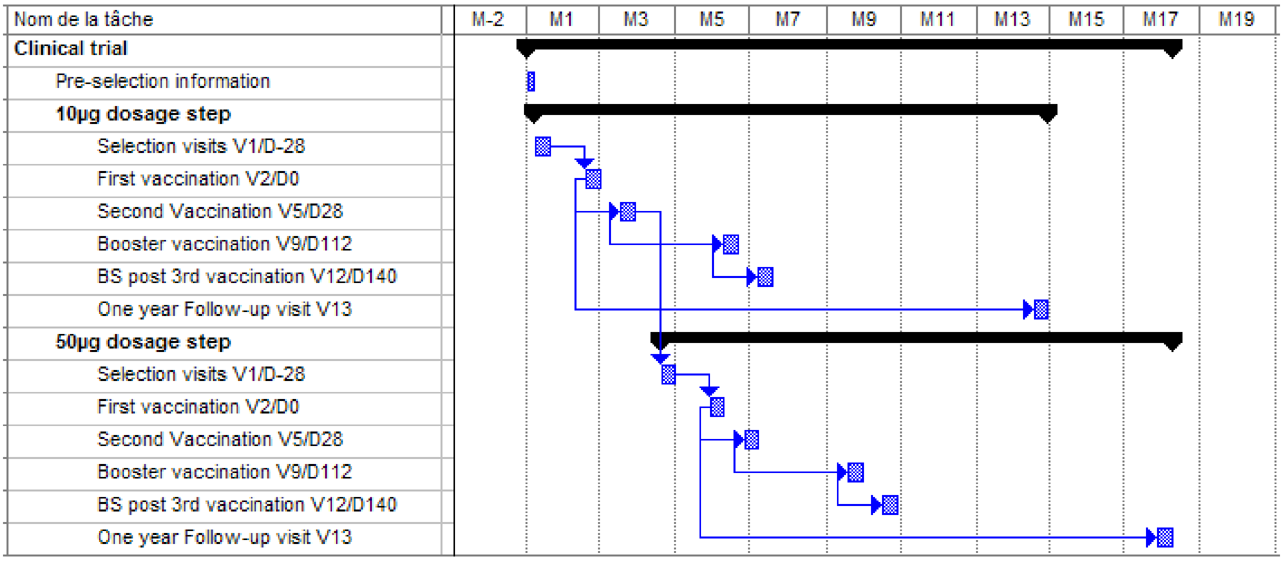

3.2.2 Vaccination, Clinical Follow-up and Serology schedule

The vaccination and serology schedule is summarized in the flow chart (see page 9). Each subject will attend at 13 standardised visits at the trial centre (Inclusion visit D-28; D0; D1; D14; D28; D29; D42; D56; D112; D113; D126; D140; D365). Two doses of vaccine at 4 week-interval will be given by intra-muscular route at D0 and D28. A booster dose will be given 12 weeks after the primary series at D112.

Blood samples will be taken at ten time points at D-28; D0; D7, D28; D35, D56; D112; D119, D140, and D365. Before each vaccination and 7 and 28 days after each vaccination a venapuncture will be performed. Biological parameters are assessed at inclusion, 7 and 28 days after each vaccination and after a year. Blood for immunologic assays will be collected at inclusion, before each vaccination, 28 days after each vaccination and a year after the first immunisation. A venapuncture will be performed ten times during the course of the study. A maximum total of 40 mL blood will be taken at each blood sampling. For each subject a maximum of 400 mL blood will be taken during the whole study.

Twenty four hours, 3 days, 7 days and 14 days after each vaccination a visit is planned to monitor adverse events at D1, D3, D7, ,D14, D29, D31, D35, D42, D113, D114, D119 and D126.

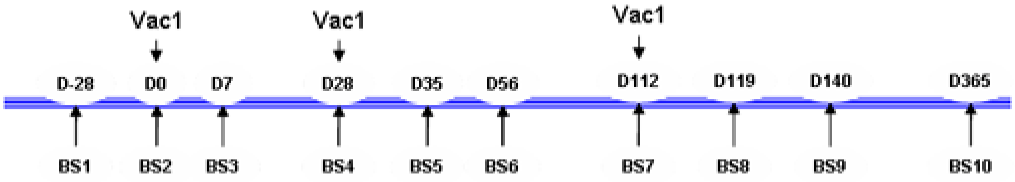

3.2.3 Trial Centres

The study will be performed at a single centre at the University Medical Center St. Radboud, Nijmegen

3.2.4 Conduct of the Trial

Interruption of the Trial

The trial may be discontinued for the following reasons:

1. administrative reasons if new data about the investigational product resulting from this or any other trials become available,
2. and/or on advice of the sponsor,
3. and/or on advice of the investigators, and/or the IRBs/Ethics Committee(s).

If a trial is prematurely terminated or suspended, the sponsor shall promptly inform the investigators, the Regulatory Authorities and the IRB of the reason for termination or suspension (as specified by the applicable regulatory requirements).

Safety Monitor

A safety monitor from UMC-St Radboud will be involved in the review of severe and serious adverse events and volunteer safety. He will be independent from both the investigator team and the sponsor team. His main responsibility will be the assessment of the events and recommendation on withdrawal of the subjects or a group of subjects from further vaccination. Please refer to the Safety Monitoring Plan 6.2 for more information.

3.3 Case Report Forms and Data Collection

3.3.1 Source Data

All data collected by the investigator is reported in the CRF's. Therefore, the CRF is also considered source data.

Since all subjects will be healthy, there is no medical file for the trial subjects, with exception of the medical file in case of adverse events/reactions resulting in a medical consultation or an hospitalisation. In this case the medical file will be considered as the source data.

For the clinical follow-up of the vaccine injection, the diaries are considered as source data. Thus they should be kept as source document in the investigator clinical file.

3.3.2 Diary

All subjects will receive diary cards which are the source document for the follow-up surveillance during the 14 days after each injection. (see section 7.1*)*

Diaries will be given to each subject at time of the vaccination visit, i.e. V2, V7 and V13. The diary will contain all solicited local and systemic reactions, as standardised data, as well as open fields for unsolicited reactions/events.

Subjects will be provided with a ruler for daily measurement of the size of local reactions at vaccination sites, and with a thermometer for daily measurement of temperature. The procedures for measurement will be explained each time a diary is provided. At day 3 and 7 after each vaccination, the investigator will check that the data collection is properly done.

The diaries will be collected on the 14-day following each vaccination, i.e. V4, V7, and V11. The data collected in the diary will be reported in the case report form.

3.3.3 Case Report Forms

All information will be recorded by the investigator or a designated person in the Case Report Forms. Case Report Forms will be completed with a black ball-point pen and signed by the investigator. For every vaccination a new section of CRF will be used. They will be labelled CRFA, CRFB, CRFC and CRFD.

Where applicable, intensity/severity data in the CRF will be collected according to a continuous scale. Only upon evaluation of the data will the intensity be categorised into mild/moderate/severe, according to the severity scales (appendix 3).

Explanations must be given for all missing information. All incorrect data must be crossed out with a single line, then signed and dated by the investigator (except date corrections). "White-out" correction fluid must not be used.

3.4 Procedures for Obtaining, Handling and Shipment of Biological Samples

3.4.1 Trial Laboratories

The biological safety parameters will be measured on serum samples at the UMCN of Nijmegen

The immunological assessment will be performed at the UMCN in Nijmegen under Dr Hermsen supervision and at BPRC under Dr Remarque's supervision

3.4.2 Obtaining Serum Samples and blood cells

Per visit, a maximum of 40 mL of blood will be collected in vacutainer tubes at visits V1, V2, V5, V7, V10, V11, V13, V13, V18and V19 by the nurse in charge of vaccination. Immediately prior to blood sampling, the person in charge of the procedure should verify subject's identity and should check that the initials on the laboratory request form are those of the subject. Then, the subject's initials must be recorded on all labels of the corresponding band. One label will be affixed onto the vacutainer tube immediately prior to blood sample drawing.

3.4.3 Handling Serum Samples

Aliquoting for Immunological tests

Aliquoting must be performed subject by subject to avoid mixing blood tubes. The following procedure must be followed:

1. After centrifugation, the person responsible for aliquoting should carry out the operation by taking the tubes one by one from the centrifuge.
2. The operator will only place in a rack five Sarstedt tubes necessary for aliquoting and will affix the completed labels onto the tubes.
3. Aliquoting will be performed according to the required volume of 1.8 mL , the number of tubes, and the priority of titrations.
4. Pre-labelled Sarstedt tubes that would not have been filled for lack of serum will be removed.
5. The subject's identification number and initials, the date of sampling, the number of aliquots obtained, and the date and time of aliquoting will be specified on the sample identification list. A label will be affixed onto the three sheets of the list. If the date is included on the vial, the technician will not be blinded for vaccine dose!
6. Comments may be made on the quality of samples (haemolysed, contaminated, etc.).
7. The next sample will be taken out of the centrifuge only when the steps 1 through 6 are completed.

Storage Conditions

Each aliquot must be frozen at -80°C. The temperature must be monitored and documented on the appropriate form (see operating guidelines) during the entire trial.

4 Selection and Withdrawal of Subjects

4.1 Inclusion Criteria

- Age > 18 and < 45 years healthy volunteers (males or females).
- General good health based on history and clinical examination.
- All volunteers have to sign the informed consent form.
- Negative pregnancy test.
- Use of adequate contraception for females up to three months after the third injection (D140).
- Reachable by phone during the whole study period (18 months).

4.2 Non-Inclusion Criteria

- History of malaria or residence in malaria endemic areas within the past six months.
- Positive serology for malaria antigen PfAMA-1
- Previously participated in any malaria vaccine study
- Symptoms, physical signs and laboratory values suggestive of systemic disorders, including renal, hepatic, cardiovascular, pulmonary, skin, immunodeficiency, psychiatric and other conditions, which could interfere with the interpretation of the study results or compromise the health of the volunteers.
- Any laboratory abnormalities on screened blood samples beyond the normal range, as defined at UMC St Radboud. Positive HIV, HBV or HCV tests.
- Volunteers should not be enrolled in any other clinical trial during the whole trial period.
- Volunteers should not receive chronic medication, especially immunosuppressive agents (steroids, immunomodulating or immunosuppressive drugs) during the three months preceding the screening visit or during the study period.
- Pregnant or lactating women.
- Volunteers unable to give written informed consent.
- Volunteers unable to be closely followed for social, geographic or psychological reasons.
- Previous history of drug or alcohol abuse interfering with normal social function during a period of one year prior to enrolment in the study.
- Volunteers should not perform exercise four hours before blood draw and should not donate blood for non study-related purposes during the entire duration of the study.
- Known hypersensitivity to any of the vaccine components (adjuvant or peptide).
- Volunteers are not allowed to receive any vaccination or gammaglobuline during a period three months prior to the first immunisation and up to six months after the 3rd immunisation. If a vaccination is necessary during this period, the volunteer will be withdrawn from the study.
- Volunteers are not allowed to travel to malaria endemic countries during the study period.

4.3 Conditions for Withdrawal from further Vaccination

1. Any serious adverse reaction as defined in section 7.1

2. Any adverse event that, according to clinical judgement of the investigator, is considered as a definite contra-indication to proceeding with the vaccinations. The holding procedure will then apply, please refer to section 6.2.2

3. The use of concomitant, chronic medication active on the immune system (steroids, immunosuppressive agents)

4. Other vaccinations received during the first 140 days of the study

5. Pregnancy

In case of intercurrent disease such as hay fever episode, flu-like syndrome, upper respiratory disease, considered as mild and expected to be promptly resolved, the vaccination could be postponed for no more than 7 days.

4.4 Conditions for Withdrawal from the Trial

Volunteers will be completely withdrawn from the trial under the following conditions:

1. Free decision by the volunteer
2. Death
3. Completely lost to follow-up

Volunteers wishing to interrupt their participation in the study before the second vaccination will be replaced. There is a premature withdrawal from the study when a volunteer stops participation before the second immunisation whatever the circumstances. If a volunteer withdraws it has to be checked that this is due to circumstances not related to the trial. If a volunteer withdraws due to adverse events, the volunteer should not be replaced.

If a subject fails to appear for a follow-up examination, extensive effort (i.e., documented phone calls and certified mail, *to be adapted to trial setting*) should be undertaken to locate or recall him/her or at least to determine his/her health status. These efforts should be documented in the subject's CRF and source documents.

4.5 Termination Classification

Any subject who does not finish the trial must be classified and the classification noted on the CRF. List termination definition, e.g., discontinuation, voluntary withdrawal /drop-out, loss to follow-up. Indicate the conditions for replacement of subjects, if any.

Definitions:

Discontinuation by the investigator: the decision to terminate the subject's participation was taken by the investigator e.g. for medical reasons (for subject's safety), for practical reasons (non respect of study schedule), serious adverse event, etc.

Voluntary withdrawal / Drop-out: a subject included in the trial is said to have dropped out after deciding, on his own volition, to terminate his participation in the trial.

A subject may decide to withdraw from the trial at any time. The investigator should make sure, however, that withdrawal was not due to an adverse event. The reason for withdrawal should be noted in the space provided for this purpose in the Case Report Form (CRF).

Failure to appear for follow-up: the subject could not be found in spite of the investigator's efforts to locate him.

5 VACCINES

5.1 Investigational Vaccine Characteristics

5.1.1 Description

Malaria vaccine. The *Pf*AMA-1-FVO[25-545] is a recombinant vaccine. The protein of vaccine interest - *Pf*AMA-1-FVO[25-545] – has a molecular weight of ~70kDa. After expression of the protein in Pichia pastoris system, *Pf*AMA-1-FVO[25-545] is purified and lyophilised. The vaccine is presented in multidose vials, and has an aspect of white, amorphous powder.

5.1.2 Formulation

Composition

- Active ingredient: (*Pf*AMA-1-FVO[25-545]): 120µg
- Other ingredients: NaHCO3; sucrose ; EDTA (trace)
- Manufacturer: Eurogentec S.A.

The vaccine will be reconstituted in saline for injection and then mixed with adjuvant so that 10µg or 50µg dosage vials in 0.5 mL solution will be prepared for injection. Three adjuvanted formulations will be prepared with either alum or Montanide or AS02A. The detailed procedure for reconstitution and mixing of vaccine is provided below.

Preparation

The reconstitution and the mixing with adjuvant will be performed under sterile conditions, in a laminar flow cabinet under the responsibility of the chief pharmacist. The adjuvanted solutions will be aliquoted in disposable vanish point syringe for a single use with a 25 mm, 23 gauge single-use needle.

Aluminum Hydroxide formulation:
Alhydrogel® is a crystalline aluminum oxyhydroxide AlOOH, also known as boehmite. The structure consists of corrugated sheets of aluminum octahedra.

Two dosages will be prepared as follows:

- 50 µg Dose :

Dissolve 120 µg AMA-1 in a volume of 600 µl saline. This gives a concentration of 200 µg/mL, add 600 µl 0.64 % Alhydrogel. Final volume will be 1.2 mL, which is sufficient for one 0.5 mL dose.

- 10 µg dose :

Dissolve 120 µg AMA-1 in a volume of 3 mL saline. This gives a concentration of 40 µg/mL, add 3 mL 0.64% Alhydrogel. Final volume will be 6 mL, which is sufficient for one 0.5 mL dose.

To facilitate adsorption of the antigen to the alum, incubate 60 minutes at room temperature. The concentration of Alhydrogel needs to be adjusted per batch, such that the final amount in one (0.5 mL) dose does not exceed 0.85 mg aluminium.

Montanide ISA 720 formulation

Montanide ISA 720 manufactured by SEPPIC, is a highly refined emulsifier from the mannide monooleate family in a natural metabolizable oil solution, yielding a water in oil emulsion when mixed with an aqueous antigen solution.

Two dosages will be prepared as follows:

- 50 µg Dose

Dissolve 120 µg AMA-1 in a volume of 320 µl buffered saline (5 mM Phosphate buffer pH 7.2, adjusted to physiologic osmotic value with NaCl). This gives a concentration of 375 µg/mL, add 880 µl ISA 720 and mix 20 times through a 21 couple piece between two (two-piece) syringes. Final volume will be 1.2 mL, which is sufficient for one 0.5 mL dose.

- 10 µg Dose

Dissolve 120 µg AMA-1 in a volume of 1.6 mL buffered saline (5 mM Phosphate buffer pH 7.2, adjusted to physiologic osmotic value with NaCl). This gives a concentration of 75 µg/mL, add 4.4 mL ISA 720 and mix 20 times through a 21 gauge couple piece between two (two-piece) syringes. Final volume will be 6 mL, which is sufficient for one 0.5 mL dose.

The syringes used for formulating Montanide ISA 720 formulations should have plungers without rubber pistons (two-piece syringe) to avoid contamination.

AS02A formulation

AS02A is an adjuvant system containing 3-deacylated monophosphoryl lipid A (MPL®) and a purified saponin in an oil-water emulsion (QS21).

Two dosages will be prepared as follows:

- 50 µg Dose

Dissolve 120 µg AMA-1 in a volume of 1.2 mL AS02A. This gives a concentration of 100 µg/mL. Final volume will be 1.2 mL, which is sufficient for one 0.5 mL dose.

- 10 µg dose

Dissolve 120 µg AMA-1 in a volume of 6 mL AS02A. This gives a concentration of 20 µg/mL. Final volume will be 6 mL, which is sufficient for one 0.5 mL dose.

5.1.3 Precaution for use

A separate sterile vanish point syringe will be used for each individual trial participant to prevent transmission of infectious agents. Before injection, the site of injection will be disinfected with a suitable antiseptic and the entire content of each syringe should be used. Do not inject in a blood vessel. As for any injectable vaccine, it is recommended to have appropriate equipment available in case of immediate allergic reactions.

5.2 Vaccination

All the enrolled subject will received three injections of a vaccine by intra-muscular route, into the deltoid muscle region, left arm at the first and third injection, and right arm at the second injection after

checking that the injection will not be done in a blood vessel. The site of injection will be recorded on the case report form.

5.3 Prior and Concomitant Therapy

Immunosuppressive or immunomodulator treatments are an exclusion criteria. If any volunteer will need chronic use of one of these medicine, the subject will be excluded from further vaccination. Furthermore, any data obtained after initiating this medication will be considered deviating.

Antipyretic or painkiller will not be allowed as preventive treatment of pain or fever, before the immunisation.

Other vaccinations will not be allowed during a three-month period before the first trial vaccine injection and within the six-month period after the third trial vaccine injection. If a vaccination is necessary during the banning time window, data collected from these subjects will be considered as deviation to the protocol.

For all other treatment, the following data will be specified and recorded on case report forms:

- Trade name and generic name (as many prescription drugs in the Netherlands are generic)
- Total daily dose
- Start and stop dates (and, if appropriate, timing requirements within the day: AM, PM, post-shot)
- Indication

5.4 Management of Vaccines

5.4.1 Labelling and Packaging

The multidose vaccine vials will be labelled with a standard label, mentioning that the vaccine use is restricted to clinical trial (see label model below).

Study code AMA-1_1_03
PfAMA-1 - 120µg
3 doses intra-muscular
Store between 2° and 8°C . **Do not freeze.**
Discard if the vaccine has been frozen.
Contact person: Prof Sauerwein, Tel : +31243610577
For clinical trial use only

Vial n°:………..

European Malaria Vaccine Initiative

5.4.2 Storage and Shipment Conditions

A staff member will be personally responsible for product management, under the supervision of the chief pharmacist.

The Monitor will determine with the investigator or the person in charge the date(s) and time(s) of delivery of products and forward the information to the Manufacturer. The vaccines will then be shipped to the centre according to the pre-determined schedule.

The person in charge of the Packaging at the Manufacturer Site will issue a dispatch note with acknowledgement of receipt attached to the package.

The person in charge of product receipt will check that the cold chain was maintained during shipment [verification of continuous temperature graph (trial conducted outside of France) / freeze watch / cold chain monitoring card]. In case of a problem, he/she should alert the Monitor immediately.

The acknowledgement of receipt will be dated and signed by the person in charge of product management. One copy will be kept archived, the other copy will be returned to:

Dr Odile Leroy
Clinical and Regulatory Affairs Director
European Malaria Vaccine Initiative
13 rue des 4 Vents
92380 Garches
France

Formulated vaccines shall be stored at a temperature ranging from +2°C to +8°C (in a refrigerator). Temperature should be monitored and documented on an appropriate form during the entire trial.

In case of deep freezing or accidental disruption of the cold chain, vaccines should never be administered and the investigator or the responsible person should contact the Monitor to receive further instructions.

5.4.3 Accountability

Products must be kept in a secure place. The investigator or the person in charge of product management, will maintain records of the product's delivery to the trial site, the inventory at the site, the dose(s) given to each subject, and the return of unused doses to the sponsor.

Should the investigator run out of product doses during the trial, he/she should alert the Monitor who will undertake the necessary steps to provide extra doses.

5.4.4 Return of Unused Products

Unused and/or open products will be returned to EMVI at the end of the vaccination period together with the form "Return of unused and/or open products" in accordance with the Monitor's instructions. The AS02A will be returned to GSK Biologicals.

6 Adverse event Management and reporting

6.1 Definitions

Definitions for the terms adverse event (or experience), adverse reaction, and unexpected adverse reaction have previously been agreed to by consensus of the more than 30 Collaborating Centres of the WHO International Drug Monitoring Centre (Uppsala, Sweden) (0). Although those definitions can pertain to situations involving clinical investigations, some minor modifications are necessary, especially to accommodate the pre-approval, development environment.

The following definitions have been agreed:

6.1.1 Adverse Event (or Adverse Experience)

An AE is any untoward medical occurrence in a clinical investigation subject, temporally associated with the use of a medicinal product, whether or not considered related to the medicinal product.

An AE can therefore be any unfavourable and unintended sign (including an abnormal laboratory finding), symptom, or disease (new or exacerbated) temporally associated with the use of a medicinal product. For marketed medicinal products, this also includes failure to produce expected benefits (i.e. lack of efficacy), abuse or misuse.

Examples of an AE include:

- Exacerbation of a chronic or intermittent pre-existing condition including either an increase in frequency and/or intensity of the condition.
- New conditions detected or diagnosed after investigational product administration even though it may have been present prior to the start of the study.
- Signs, symptoms, or the clinical sequelae of a suspected interaction.
- Signs, symptoms, or the clinical sequelae of a suspected overdose of either investigational product or a concurrent medication (overdose per se should not be reported as an AE/SAE).
- Signs, symptoms temporally associated with vaccine administration.

Examples of an AE do not include:

- Medical or surgical procedure (e.g., endoscopy, appendectomy); the condition that leads to the procedure is an AE.
- Situations where an untoward medical occurrence did not occur (social and/or convenience admission to a hospital).
- Anticipated day-to-day fluctuations of pre-existing disease(s) or condition(s) present or detected at the start of the study that do not worsen.
- The disease/disorder being studied, or expected progression, signs, or symptoms of the disease/disorder being studied, unless more severe than expected for the subject's condition.

AEs may include pre- or post-treatment events that occur as a result of protocol-mandated procedures (i.e. invasive procedures, modification of subject's previous therapeutic regimen).

N.B. AEs to be recorded as endpoints (solicited events) are described in Section 7.1. All other AEs will be recorded as UNSOLICITED AEs.

Example of events to be recorded in the medical history section of the CRF:

- Pre-existing conditions or signs and/or symptoms present in a subject prior to the start of the study (i.e. prior to the first study procedure) should be recorded in the medical history section of the subject's CRF.

6.1.2 Serious Adverse Event

During clinical investigations, adverse events may occur which, if suspected to be medicinal product-related (adverse drug reactions), might be significant enough to lead to important changes in the way the medicinal product is developed (e.g., change in dose, population, needed monitoring, consent forms). This is particularly true for reactions that, in their most severe forms, threaten life or function. Such reactions should be reported promptly to regulators.

Therefore, special medical or administrative criteria are needed to define reactions that, either due to their nature ("serious") or due to the significant, unexpected information they provide, justify expedited reporting. To ensure no confusion or misunderstanding of the difference between the terms "serious" and "severe," which are not synonymous, the following note of clarification is provided:

The term "severe" is often used to describe the intensity (severity) of a specific event (as in mild, moderate, or severe myocardial infarction); the event itself, however, may be of relatively minor medical significance (such as severe headache). This is not the same as "serious," which is based on patient/event outcome or action criteria usually associated with events that pose a threat to a patient's life or functioning. Seriousness (not severity) serves as a guide for defining regulatory reporting obligations

A serious adverse event (experience) or reaction is any untoward medical occurrence that at any dose:

* results in death,

* is life-threatening,

NOTE: The term "life-threatening" in the definition of "serious" refers to an event in which the patient was at risk of death at the time of the event; it does not refer to an event which hypothetically might have caused death if it were more severe.

* requires inpatient hospitalisation or prolongation of existing hospitalisation,

* results in persistent or significant disability/incapacity, or

* is a congenital anomaly/birth defect.

Medical and scientific judgement should be exercised in deciding whether expedited reporting is appropriate in other situations, such as important medical events that may not be immediately life-threatening or result in death or hospitalisation but may jeopardise the patient or may require intervention to prevent one of the other outcomes listed in the definition above. *These should also usually be considered serious.*

6.1.3 Clinical laboratory parameters and other abnormal assessments qualifying as adverse events and serious adverse events

Abnormal laboratory findings (e.g., clinical chemistry, haematology, urinalysis) or other abnormal assessments that are judged by the investigator to be clinically significant will be recorded as AEs or SAEs if they meet the definition of an AE, as defined in Section 6.1.1 or SAE, as defined in Section 6.1.2. Clinically significant abnormal laboratory findings or other abnormal assessments that are detected during the study will be reported as AEs or SAEs.

The investigator will exercise his or her medical and scientific judgement in deciding whether an abnormal laboratory finding or other abnormal assessment is clinically significant.

6.2 Safety Monitoring Plan

6.2.1 Role of Local Safety Monitor

The Safety Monitor (SM) will be an experienced clinician qualified to evaluate safety data from clinical trials of new vaccines. He/she will not be directly involved in the clinical trial and will be based in the Radboud University Nijmegen Medical Centre. All SAEs will be reported to him/her. In exceptional circumstances, for example a death possibly related to vaccination, or under conditions as outlined in below in the safety monitoring plan, he/she will have the authority to suspend vaccination pending discussion with the sponsor and collaborators. He will hold the code-break information and under conditions as outlined below, he will have the authority to unblind individual subjects. All unblinding should be reported immediately to the sponsor and GSK Biologicals, if the adjuvant concerned is AS02A (to the attention of the safety physician).

The SM's role will include:

ongoing review of serious adverse events

unblinding a subject if deemed necessary to allow for adequate treatment.

6.2.2 Holding rules

Vaccination will be performed in a staggered fashion in 2 parts. All the primary immunisation doses of the 10µg antigen in the 3 adjuvant groups (i.e. AS02A, Aluminium hydroxide or Montanide 720) will be delivered prior to starting with vaccination with 50µg antigen in either AS02A, Aluminium hydroxide or Montanide 720. After each vaccination, safety data will be provided in tables by groups A, B or C by the data manager. The safety tables will be reviewed by the investigators, the Safety Monitor, and the Sponsor.

If there are no safety issues the subsequent dosage can be given.

Vaccination of subsequent dosage can be put on hold by the Sponsor on advice of the Safety Monitor if the following criteria are met:

- If, based on the review of the safety data tables provided by the data manager, 5 on 10 subjects in a particular group (A, B or C) are found to have developed a Grade 3 adverse event, related to vaccination and persisting at Grade 3 for > 48 hours during the 14 follow-up days after vaccination, a decision of un-blinding will be made by the Safety Monitor. Subsequent vaccination of that group will be put on hold pending discussion with the investigator, the sponsor and also GSK Biologicals if it applies to subjects vaccinated with the adjuvant AS02A.
- SAE related to vaccination - the SM will unblind the individual and subsequent vaccination of that adjuvant group may be put on hold pending discussion with the investigator, the sponsor and also GSK Biological if it applies to subjects vaccinated with the adjuvant AS02A.

Activation of the Holding Rules requires a thorough review by the Safety Monitor of blinded reactogenicity and safety data and discussion with the investigator, the sponsor and also GSK Biologicals if it applies to subjects vaccinated with the adjuvant AS02A.

6.2.3 Review of Safety Data by the Safety Monitor

Vaccinations are scheduled every month. Reactogenicity and safety evaluations will be collected over a period of 14 days (day of vaccination and subsequent 13 days after each vaccination) after each vaccination. The following events must occur within the specified timeframe to ensure proceeding to the next vaccination as per schedule.

The data manager must generate a summary of all reactogenicity reported by subjects during the 14 days follow-up period after vaccination and also any safety laboratory values outside the normal ranges. This report will be sent to the Safety Monitor and EMVI monitor by Day 19.

The Safety Monitor and EMVI monitor must review the safety data for each subject within 2 business days and if warranted instruct the site and the sponsor to withdraw individual subjects and/or suspend further vaccination, at least 4 business day prior to the next vaccination.

6.2.4 Process for restarting vaccination

Although the vaccination of a group may be put on hold, further vaccination of that group will restart only if all parties (the medical monitor, the Sponsor, the Principal Investigator and GSK Biologicals) agree to a resumption of vaccination.

Within 5 working days of the SM placing vaccination of a group on hold, the Sponsor will organise a meeting (via teleconference, videoconference, or face-to-face) to review and discuss the safety data and the events leading to the hold order. At least two working days prior to this meeting, the Sponsor will disseminate copies of all relevant safety data to all meeting participants.

6.2.5 Process for stopping vaccination of a group or of the trial

In the event that a vaccination of a particular group is stopped, the Sponsor will inform the IRB through the investigator. A report will be written detailing the rationale used for reaching this decision.

6.3 Safety Data Collection and Management Procedures

6.3.1 Expected Adverse Vaccine Reactions:

As for any adjuvanted vaccine when administered intra-muscularly, local reactions are expected. Systemic reactions are less often observed, however a standardized data collection of adverse reactions will be organized (see section 7.1).

The clinical experience with Montanide, Alhydrogel and AS02A has shown that the most frequent local reactions are pain, erythema and swelling at the site of injection. Montanide, Alhydrogel are know also to be responsible of induration at the site of injection,

Data on expected systemic reactions is unclear. Therefore, general signs, i.e. fever, headache, fatigue, and drowsiness, will especially be noted.

In case of a severe local skin reaction, either uni-lateral or contra-lateral, a skin biopsy may be part of the procedure as part of the clinical evaluation.

6.3.2 Safety Data Collection

All the adverse events/reactions, whatever observed by the investigator or by the subject, will be carefully and accurately documented in the case report form by the investigator. For each event/reaction the following details will be recorded : 1) description of the event(s)/reaction(s), 2) date and time of occurrence, 3) duration, 4) intensity, 5) relationship with the vaccine, 6) action taken including treatment, 7) outcome. Safety assessments will be obtained, and recorded by the investigator Dr. M. Roestenberg. She will not know which vaccine formulation the subject has received. Systemic adverse reactions will especially be monitored during 30 minutes after each injection; any solicited and unsolicited reactions/events will be recorded the following 14 days.

This includes all local (e.g., redness, swelling, etc.) and systemic (e.g., fever, headache, vomiting, etc.) reactions for both immediate and full duration of follow-up reactions. The timing of the assessment relative to the dosing schedule will be noted .

Intensity scales: See appendix 3. Symptoms will be ranked as (1) mild, (2) moderate, or (3) severe. Mild is an awareness of symptoms that are easily tolerated and do not interfere with usual daily activity. Moderate is discomfort that interferes with or limits usual daily activity. Severe is disabling, inability to perform usual daily activity, resulting in absenteeism or required bed rest. The intensity of symptoms will be noted, if possible on a continuous scale in the source record. Only when analysing the data will they be categorised into mild/moderate/severe.

Local symptoms will be ranked as mild, moderate or severe by the size of the reaction (see section 7.1.2)

6.3.3 Time period, frequency, and method of detecting adverse events and serious adverse events

All AEs occurring within one month following administration of each dose of vaccine must be recorded on the Adverse Event form in the subject's CRF, irrespective of severity or whether or not they are considered vaccination-related.

The standard time period for collecting and recording SAEs will begin at randomization or the first receipt of vaccine and will end one month following administration of the last dose of study vaccine for each subject. See Section 6.3.4 for instructions for reporting and recording SAEs.

Additionally, in order to fulfil international reporting obligations, SAEs that are related to study participation (e.g. procedures, invasive tests, a change from existing therapy) or are related to a concurrent medication will be collected and recorded from the time the subject consents to participate in the study until she/he is discharged.

The investigator will inquire about the occurrence of AEs/SAEs at every visit/contact during the study and throughout the follow-up phase as appropriate.

All AEs either observed by the investigator or one of his clinical collaborators or reported by the subject spontaneously or in response to a direct question will be evaluated by the investigator. AEs not previously documented in the study will be recorded in the Adverse Event form within the subject's CRF. The nature of each event, date and time (where appropriate) of onset, outcome, intensity and relationship to vaccination should be established. Details of any corrective treatment should be recorded on the appropriate page of the CRF.

As a consistent method of soliciting AEs, the subject should be asked a non-leading question such as:

"Have you felt different in any way since receiving the vaccine or since the previous visit?"

AEs already documented in the CRF, i.e. at a previous assessment, and designated as “not recovered/not resolved” or “recovering/resolving” should be reviewed at subsequent visits, as necessary. If these have resolved, the documentation in the CRF should be completed.

N.B. If an AE changes in frequency or intensity during the specified reporting period, a new record of the event will be entered.

When an AE/SAE occurs, it is the responsibility of the investigator to review all documentation (e.g., hospital progress notes, laboratory, and diagnostics reports) relative to the event. The investigator will then record all relevant information regarding an AE/SAE on the CRF or SAE Report Form respectively. It is not acceptable for the investigator to send photocopies of the subject’s medical records to EMVI and GSK Biologicals in lieu of the appropriate completed AE/SAE pages. However, there may be instances when copies of medical records for certain cases are requested by EMVI or GSK Biologicals. In this instance, all subject identifiers will be blinded on the copies of the medical records prior to submission to EMVI and GSK Biologicals.

The investigator will attempt to establish a diagnosis of the event based on signs, symptoms, and/or other clinical information. In such cases, the diagnosis should be documented as the AE/SAE and not the individual signs/symptoms.

6.3.4 Assessment of causality

The investigator is obligated to assess the relationship between investigational product and the occurrence of each AE/SAE. The investigator will use clinical judgement to determine the relationship. Alternative causes, such as natural history of the underlying diseases, concomitant therapy, other risk factors and the temporal relationship of the event to the investigational product will be considered and investigated. The investigator will also consult the Investigator Brochure and/or Product Information, for marketed products, in the determination of his/her assessment.

There may be situations when a SAE has occurred and the investigator has minimal information to include in the initial report to EMVI and GSK Biologicals. However, it is very important that the investigator always makes an assessment of causality for every event prior to transmission of the SAE Report Form to EMVI and GSK Biologicals. The investigator may change his/her opinion of causality in light of follow-up information, amending the SAE Report Form accordingly. The causality assessment is one of the criteria used when determining regulatory reporting requirements.

All solicited local (injection site) reactions will be considered causally related to vaccination. Causality of all other AEs should be assessed by the investigator using the following question:

Is there a reasonable possibility that the AE may have been caused by the investigational product ?

NO	The AE is not causally related to administration of the study vaccine(s). There are other, more likely causes and administration of the study vaccine(s) is not suspected to have contributed to the AE.
YES	There is a reasonable possibility that the vaccine(s) contributed to th AE.

Non-serious and serious AEs will be evaluated as two distinct events. If an event meets the criteria to be determined “serious” (see Section 6.1.2 for definition of serious adverse event), it will be examined by the investigator to the extent to be able to determine ALL contributing factors applicable to each serious adverse event.

Other possible contributors include:

- Medical history
- Other medication
- Protocol required procedure
- Other procedure not required by the protocol
- Lack of efficacy of the vaccine(s), if applicable
- Erroneous administration
- Other cause (specify)

6.3.5 Medically attended visits

For each solicited and unsolicited symptom the subject experiences, the subject will be asked if they received medical attention defined as hospitalization, an emergency room visit or a visit to or from medical personnel (medical doctor) for any reason and this information will be recorded in the CRF.

6.4 Follow-up of adverse events and serious adverse events and assessment of outcome

After the initial AE/SAE report, the investigator is required to proactively follow-up on each subject and to provide further information to EMVI and GSK Biologicals on the subject's condition.

All AEs and SAEs documented at a previous visit/contact and designated as not recovered/not resolved or recovering/resolving will be reviewed at subsequent visits/contacts.

Investigators will follow-up subjects:

- with SAEs or subjects withdrawn from the study as a result of an AE, until the event has resolved, subsided, stabilized, disappeared, the event is otherwise explained, or the subject is loss to follow-up;
- or, in the case of other non-serious AEs, until they complete the study or they are loss to follow-up.

Clinically significant laboratory abnormalities will be followed up until they have returned to normal, or a satisfactory explanation, not related to the trial, has been provided. Additional information (including but not limited to laboratory results) relative to the subsequent course of such an abnormality noted for any subject must be made available to the Study Monitor.

EMVI -for all SAEs- and GSK Biologicals –only when AS02 is concerned- may request that the investigator perform or arrange for the conduct of supplemental measurements and/or evaluations to elucidate as fully as possible the nature and/or causality of the AE or SAE. The investigator is obliged to assist. If a subject dies during participation in the study or during a recognized follow-up period, EMVI -for all SAEs- and GSK Biologicals –only when AS02 is concerned- will be provided with a copy of any available post-mortem findings, including histopathology.

New or updated information will be recorded on the originally completed SAE Report Form, with all changes signed and dated by the investigator. The updated SAE report form should be resent to EMVI -for all SAEs- and GSK Biologicals –only when AS02 is concerned- within 24 hours of receipt of the follow-up information as outlined in Section6.4.2.

Outcome of any non-serious AE occurring within 30 days post-vaccination (i.e. unsolicited AE) or any SAE reported during the entire study will be assessed as:

- Recovered/resolved
- Not recovered/not resolved
- Recovering/resolving
- Recovered with sequelae/resolved with sequelae
- Fatal (SAEs only)

6.5 Reporting of Serious Adverse Events

6.5.1 Time frames for reporting of SAEs

SAEs will be reported promptly once the investigator determines that the event meets the protocol definition of an SAE. The investigator or designate will report to EMVI monitor -for all SAEs- and GSK Biologicals –only when AS02 is concerned- WITHIN 24 HOURS OF HIS/HER BECOMING AWARE OF THESE EVENTS. Additional or follow-up information relating to the initial SAE report is also to be

reported to the EMVI monitor -for all SAEs- and GSK Biologicals Study Contact for Serious Adverse Event Reporting –only when AS02 is concerned- within 24 hours of receipt of such information.

If information on the adjuvant used is necessary for the treatment of the subject, the code will be broken by the investigator.

The investigator will always inform the safety monitor, who will, in case the code was not broken, break the code for sending the information on AS02 to GSK.

Notification should be made

1. to EMVI for all the SAEs

by phone; then the investigator should immediately send the **completed SAE report** to EMVI

or by email or fax by sending the **completed SAE report,** with an acknowledgment of receipt.

2. to GSK for SAEs occurring in the groups receiving the AS02A formulations

by fax by sending the **completed SAE report**

3. to the IRB

6.5.2 Completion and transmission of serious adverse event reports

Once an investigator becomes aware that a SAE has occurred in a study subject, she/he will report the information to the EMVI monitor and in case of AS02 to GSK Biologicals Study Contact for reporting SAEs within 24 hours. The SAE Report Form will always be completed as thoroughly as possible with all available details of the event, signed by the investigator (or designee). If the investigator does not have all information regarding an SAE, he/she will not wait to receive additional information before notifying Study Contact of the event and completing the form. The form will be updated when additional information is received and forwarded to EMVI and eventually to GSK WITHIN 24 HOURS.

The investigator will always provide an assessment of causality at the time of the initial report as described in Section 6.3.4.

Facsimile (Fax) transmission of the SAE Report Form is the preferred method to transmit this information to the Study Contact for Reporting SAEs. In rare circumstances and in the absence of facsimile equipment, notification by telephone is acceptable, with a copy of the SAE Report Form sent by overnight mail. Initial notification via the telephone does not replace the need for the investigator to complete and sign the SAE Report Form within 24 hours.

In the event of a death determined by the investigator to be related to vaccination, sending of the fax must be accompanied by telephone call to EMVI monitor and in case of AS02 involvment to the GSK Biologicals Study Contact for Reporting SAEs.

Study Contact for Reporting SAEs to EMVI Dr Hildur Blythman

To be completed

Back-up Study Contact for Reporting SAEs to EMVI
Dr Odile Leroy
Tel: 33+1 47 95 17 81
Fax: 33+1 47 95 17 81
Outside office hours
Tel: 33+6 86 78 31 49
Email : odile.Leroy@wanadoo.fr

GSK Biologicals contacts: GSK Biologicals
Protocol tracking number: 104288 (Malaria-043)
Clinical Development Manager **Opokua Ofori-Anyinam, PhD**
Tel : +32 2 656 9863
Fax : +32 2 656 80 44
Email : opokua.ofori-anyinam@gskbiologicals.com

Clinical Study Coordinator and Study Contact for Reporting SAEs to GSK
Isabelle Ramboer, PhD
Tel : +32 2 656 6820
Fax : +32 2 656 80 44
Email : isabelle.ramboer@gskbiologicals.com
Contact for Code Break

GSK Biologicals Clinical Safety Physician and Back-up Study Contact for Reporting SAEs
Tel : +32 2 656 87 98
Fax : +32 2 656 80 09
Mobile phone for 7/7 day availability: +32 477 404 713

6.5.3 Regulatory reporting requirements for serious adverse events

The investigator will promptly report all SAEs to EMVI and in case of AS02 to GSK in accordance with the procedures detailed in Section 6.5.2. EMVI and GSK Biologicals have a legal responsibility to promptly notify, as appropriate, both the local regulatory authority and other regulatory agencies about the safety of a product under clinical investigation. Prompt notification of SAEs by the investigator to the Study Contacts for Reporting SAEs is essential so that legal obligations and ethical responsibilities towards the safety of other subjects are met.

The investigator, or responsible person according to local requirements, will comply with the applicable local regulatory requirements related to the reporting of SAEs to regulatory authorities and the IRB/IEC.

Expedited Investigator Safety Reports (EISR) are prepared according to EMVI and GSK Biologicals policy and are forwarded to investigators as necessary. An EISR is required for:

- development compounds (i.e. compounds not marketed), if the event is serious, unexpected and has a suspected relationship to study drug treatment. Expected adverse events for development compounds will be described in the Development Core Safety Information (DCSI) in the Investigator Brochure (IB).
- The purpose of the EISR is to fulfil specific regulatory and Good Clinical Practice (GCP) requirements, regarding the product under investigation.
- An investigator who receives an EISR describing a SAE or other specific safety information from EMVI or GSK Biologicals will file it with the Investigator Brochure and will notify the IRB/IEC, if appropriate according to local requirements.

6.5.4 Post study adverse events and serious adverse events

A post-study AE/SAE is defined as any event that occurs outside of the AE/SAE detection period defined in Section 6.3.3. Investigators are not obligated to actively seek AEs or SAEs in former study participants.

However, if the investigator learns of any SAE, including a death, at any time after a subject has been discharged from the study, and he/she considers the event reasonably related to the investigational product, the investigator will promptly notify the Study Contact for Reporting SAEs.

6.6 Pregnancy

Subjects who become pregnant during the study (one month after the first dose or three months after the second dose) must not receive additional doses of study vaccine but may continue other study procedures at the discretion of the investigator.

The investigator, or his/her designee, will collect pregnancy information on any subject who becomes pregnant while participating in this study. The investigator, or his/her designee, will record pregnancy information on the Pregnancy Report Form and submit it to EMVI and GSK within 24 hours of learning of a subject's pregnancy, regardless of the adjuvant used. The investigator will inform the safety monitor as soon as possible. The safety monitor will break the code for the pregnant subject and inform GSK and EMVI about the adjuvant used. The investigator will remain to be blinded.

The subject will be followed to determine the outcome of the pregnancy. At the end of the pregnancy, whether that be full-term or prematurely, information on the status of the mother and child will be forwarded to EMVI and GSK. Generally, follow-up will be no longer than six to eight weeks following the estimated delivery date.

While pregnancy itself is not considered an AE or SAE, any pregnancy complication or elective termination of a pregnancy for medical reasons will be recorded as an AE or a SAE, as described in Section 6.3 and 6.4, and will be followed as described in Section 6.5.

A spontaneous abortion is always considered to be a SAE and will be reported as described in Section 6.5. Furthermore, any SAE occurring as a result of a post-study pregnancy AND considered reasonably related in time to receipt of the investigational product by the investigator, will be reported to EMVI and GSK Biologicals as described in Section 6.5.4. While the investigator is not obligated to actively seek this information from former study participants, he/she may learn of a pregnancy through spontaneous reporting.

Information on pregnancies identified during the screening phase/prior to vaccine administration does not need to be collected; this information need not be communicated to safety.

6.7 Treatment of adverse events

Treatment of any adverse event is at the sole discretion of the investigator and according to current good medical practice. Any medication administered for the treatment of an AE should be recorded in the subject's CRF.

6.8 Loss to follow-up Procedures

If a subject fails to appear for a follow-up examination, extensive effort (i.e., documented phone calls and certified mail) should be undertaken to locate or recall him/her or at least to determine his/her health status. These efforts should be documented in the subject's CRF and source documents.

7 EVALUATION CRITERIA

7.1 Primary Evaluation Criterion : Safety

7.1.1 Definition of the Criterion

The safety profile will be assessed on the following criteria

- Immediate reactogenicity; defined as any systemic adverse reactions occurring within 30 minutes after each injection,
- Local and systemic reactogenicity measured from day 0 to day 14 after each dose
- Any unsolicited adverse event resulting in a visit to a physician between each injection and one month after the third dose.
- Any Serious Adverse Event (SAE) occurring from the inclusion through out the study. The relationship of the adverse event to the study vaccine will be established by the investigator, using the following definitions : related or not related.
- Biological safety, one month after each vaccination, in reference with the baseline before the first dose, by measuring the following :
  - RBC, hemoglobin, hematocrit, MCV, MCH, MCHC, platelets, WBC with differential counts
  - Potassium, sodium, ASAT, ALAT, total bilirubin, alkaline phosphatase, γGT, creatinin

7.1.2 Parameters to be measured

The rate, duration and severity of signs and symptoms during the 14 days after the immunization will be measured.

1. Local reactions

The following local reactions will be recorded during the 14 days following the vaccination (D0 and D14 follow-up) :

1. Any local

2. **Pain :** Intensity and duration
3. **Redness :** Size (mm) and duration
4. **Swelling:** Size (mm) and duration
5. **Other Local :** Intensity or size and duration

2. Systemic reactions

The following systemic reactions will be recorded immediately after the injection (30 minutes) and during the 14 days following the vaccination (D0 and D14 follow-up):

1. **Any systemic**
2. **Fatigue :** Duration and intensity
3. **Fever : I**ntensity and duration
4. **Headache** Intensity and duration
5. **Malaise:** Intensity and duration
6. **Myalgia:** Intensity and duration
7. **Joint pain:** Intensity and duration
8. **Gastro-intestinal symptoms:**
    - **Nausea:** Intensity and Duration
    - **Diarrhoea :** Intensity and Duration
    - **Vomiting :** Intensity and Duration
    - **Abdominal pain:** Intensity and Duration
9. **Contro-lateral local reaction** : Intensity and Duration
10. **Other systemic :** Intensity and Duration

7.1.3 Method and Timing of Measurement

1. Local reactions

The duration of the **pain** will be measured in minutes, hours or days, and the severity scale as 1) mild: easily tolerated, does not interfere with daily life 2) moderate: discomfort that interferes with or limits usual daily activity 3) severe: disabling, inability to perform usual daily activity, including a functional limitation of the movement.

The duration of redness, swelling, and other local reaction will be measured in hours or in days.

The size of redness, swelling, will be measured in cm with a rule, the maximum length will be reported on the CRF, in mm. **Redness, or swelling**. of less than 20 mm is mild, of 20-50 mm is moderate, and of greater than 50 mm is severe

Solicited local reactions will be recorded at visits V2; V3, V4; V5, V6, V, V8, V9, V10, V11, V13, V14, V15, V16 and V17. The subjects will report daily the local reactions in their diary from day 1 to day 14 after the vaccination.

2. Systemic reactions

The duration in hours or in days of all systemic reaction will be monitored by the investigator, as well as the time interval of occurrence after the last vaccination.

Oral temperature will be measured with a thermometer and the intensity of **fever** will be defined as 1) mild when comprised between 37°,5°C and38°C, 2) moderate when comprised between 38°C and 39,0°C and 3) severe when > 39,0°C.

The systemic reactions will be monitored at visits V2; V3, V4; V5, V6, V7, V8, V9, V10, V11 and V12. The subjects will report daily the systemic reactions in their diary from day 0 to day 14 after the vaccination.

7.2 Secondary Evaluation Criteria : Immunogenicity

The immunogenicity of the different formulation of the vaccine will be assessed on the level and the quality of circulating antibodies as well as the stimulation of the T-cell immune response.

7.2.1 Humoral Immune response

1. Definition

The humoral response to the vaccine antigens will be assessed by measuring the level of IgG, before each vaccination, one month after each vaccination and one year after the first vaccination.

An IFA for at least two parasite strains will be employed to verify that the antibodies elicited by the vaccine recognise the native protein on merozoites.

-

2. Parameters to be Measured, Method and Timing of Measurement

IgG titres will be measured by ELISA. In the event IgG titres are undetectable, additional IgM ELISA's will be performed.

The level of IgG will be expressed in concentration (µg/mL of AMA-1-specific IgG antibody, calculated from a calibrated reference human pool serum provided by Dr Remarque).

The ELISA for IgG titres will be performed at samples obtained at D0, D28, D56, D112, D140 and D365.

An IFA, with at least 2 parasite strains will be performed at D0 and D140. The antibody to be detected in the IFA will be IgG.

These tests will be performed by the Radboud University Nijmegen Medical Center.

7.2.2 Cellular Immune response:

1. Definition:

The cellular immune response to the vaccine antigen will be assessed by measuring T cell proliferation and cytokine production by ELISPOT following in vitro stimulation.

2. Parameters to be Measured, Method and Timing of Measurement

Results of the T-cell proliferation assay will be expressed as stimulation index (S.I.) calculated as the ratio of the geometric mean of the [3H]-thymidine incorporation (cpm) in the presence (antigen concentration yielding the highest uptake) and in the absence of antigen.

T-cell proliferation will be performed with a standard proliferation assay employing tritium thymidin. Cytokine production will be assayed by ELISPOT for the cytokines IL-5 and IFN-γ (10-11).

These measurements will be performed one month before and one month after the second and the third vaccinations (at D0, D56 and D140).

These tests will be performed by the Radboud University Nijmegen Medical Center.

7.3 Exploratory Evaluation Criteria:

1. Definition of the Criteria

The **quality of the humoral immune response** will be assessed by measuring :

- IgG1, IgG2, IgG3, IgG4 subclasses by ELISA
- IgG responses to AMA-1 FVO domains by ELISA
- IgG responses to allelic variants of AMA-1 by ELISA
- Ability to block parasite growth in vitro by a Plasmodium Inhibition Assay against two parasite strains
- Ability to recognise the moab 4G2 binding site by a competition ELISA
- Ability to recognise 4G2 mimotope peptides by ELISA
- IgG avidity by Biacore analysis

Qualitative characteristics of the antibodies, such as avidity should be considered in evaluating the efficacy of the vaccine. Antibody avidity describes the strength with which an antibody binds to a complex antigen. Antibodies elicited by immunization tend to be heterogeneous with regard to avidity (Pauling 1944). As antibody response matures, average affinity of antibodies will increase (Inouye et al. 1984). Depending on the vaccine formulation avidity of AMA1 antibodies may vary, reflecting immune response maturation.

- These endpoints are purely exploratory and of research interest. They are not part of the safety assessment. These experiments will not be run in GLP manner.

2. Parameters to be Measured, Method and Timing of Measurements

- IgG1, IgG2, IgG3, IgG4 subclasses by ELISA on samples obtained at D0, D56 and D140
- The domain specificity will be measured using a standard ELISA with AMA-1 domains as coating antigen on samples obtained at D0, D140, D56 and D365
- The allele specificity will be measured using a standard ELISA with several allelic variants of AMA-1 as coating antigen on samples obtained at D0, D28, D56, D112, D140 and D365
- The Plasmodium Inhibition Assay will be performed against three parasite strains on samples obtained at days 0, 56, 140 and 365
- The ability to recognise the moab 4G2 binding site by a competition ELISA will be performed on samples obtained at days 0, 56, 140 and 365
- The ability to recognise 4G2 mimotope peptides by ELISA on samples obtained at days 0, 56, 140 and 365
- IgG avidity by Biacore analysis on samples obtained at days 0, 56 and 140

A biophysical method that is gaining acceptance for characterizing (high-) affinity interactions is the Biacore. With this method, one reactant is immobilized onto a biosensor chip and the other reactant is flowed across the surface, while the binding of the two reactants is followed in real time by surface plasmon resonance [R. Karlsson and A. Fält 1997, Myszka 1997]. For most high-affinity interactions the association rate constant k_a and dissociation rate constant k_d are determined directly, and the K_D is calculated by the quotient of the rate constants, k_d/k_a.

The above-described tests will be performed at the BPRC, with the exeption of IgG avidity with Biacore analysis, which will be performed by the Radboud University Nijmegen Medical Center.

7.4 Statistical Methods and Data Analysis

7.4.1 Principal Objective

To evaluate the safety of 3 doses given at D0, D28 and D112 of 2 different dosages of AMA-1 (10 µg or 50 µg) adjuvanted either with Alhydrogel or AS02A or Montanide ISA 720 in healthy adults not previously exposed to the parasite *Plasmodium falciparum*

7.4.2 Determination of the Sample Size

The total number of subject will be 60. A total of 6 groups of 10 subjects per group will be recruited. It is not expected to have loss to follow-up.

7.4.3 Data Set to be analysed

1. Definition of Population

Given the above mentioned considerations it is planned to enrol 60 subjects in the trial, to be randomised over the 6 treatment arms.

The statistical analysis shall be performed on two types of population, depending the subject compliance to the protocol:

1. **per protocol** : the vaccination, blood sampling and visits number as well as the time-interval between vaccination and follow-up visits are respected, as they are defined in the section 3.2.2 and in the flow chart. All subjects who have not been compliant with the protocol will be excluded from this analysis but will be described in the final report. The protocol compliance criteria are the following :

- subjects complying with the inclusion and non-inclusion criteria
- subjects who have respected the randomisation
- subjects who have respected the number of vaccination and the time-intervals between the vaccination:
  - V2 : 1st vaccination: DO, interval V1+4 weeks (± 7days)
  - V7 : 2nd vaccination D28 , interval V2+4 weeks (± 2 days)
  - V13: 3rd vaccination D112 , interval V5+12 weeks (± 7days)
- Subjects having respected the number of blood samples for biological safety evaluation, as well as the intervals between blood samples :
  - V1, V2, V5, V7, V10, V11, V12, V16, V18 and V19 samples at D-28, D0, D28, D56, and D140, interval 4 weeks (± 2 days) with the preceding sample
  - V9, sample at D112, interval 12 weeks (± 7days) with the preceding sample.

on randomised subjects: All subjects who have received at least one vaccine dose, will be included in the analysis. If a subject is not vaccinated for reasons unrelated to the primary outcome measure (missing at random [MAR] or missing completely at random [MCAR]), that subject will be excluded from further analyses. In the event a subject withdraws from the study due to side effects, the last observation will be carried forward in all subsequent analyses (LOCF) of the primary outcome measure.

2. *Population used in analyses*

The primary analysis will be conducted on all subjects who have received at least one vaccine dose.

7.4.4 Statistical Methods

The analysis shall be descriptive; the sample size does not allow any comparison between groups. For categorical variables, frequency distributions, by vaccination group, will be presented. For continuous variables, box-whisker plots, medians, inter-quartile ranges and ranges will be presented by vaccination group.

The analysis plan will be available before the lock of the data base for the interim analysis after the second injection of vaccine.

7.4.5 Data Management

Throughout regular data collection and monitoring, clinical data reported on CRFs and/or relevant serological/biological samples analysis results scheduled in the protocol will be integrated into the internal clinical data management system.

For each batch of data, double entry, quality control and triggers to computerised logic and/or consistency checks will be systematically applied in order to detect errors or omissions. After integration of all corrections in the complete set of data, the database will be locked and saved before being released for statistical analysis. Each step of this process will be monitored through the implementation of individual passwords and/or regular backups in order to maintain appropriate database access and to guarantee database integrity.

8 ETHICAL CONSIDERATION

8.1 Informed Consent

The subject or the subject's legally acceptable representative should give written informed consent before being included in the trial, after having been informed of the nature of the trial, the potential risks and their obligations (Appendix 2).

Informed consent forms will be provided in triplicate (original kept by the investigator, one copy kept by the subject or the subject's legally acceptable representative and one copy kept by EMVI in a sealed envelope).

8.2 Subject Benefit and Risks

As for any phase I clinical trial, the subject benefits are minimal in absence of any data on efficacy it is not possible to claim for any protection from malaria infection after vaccination.

Risks will follow according to protocol and RIVM evaluation following BIF regulations.

8.3 Ethical Review

Protocol review

Before the inclusion of the first subject in the centre, the protocol must be approved by all the Ethics Committee(s) and Regulatory Authorities (if applicable). Then it must be signed by the investigator(s) and sponsor's representatives.

The financial agreement must be approved prior to the start of the trial.

Ethical Considerations

This trial will be conducted in accordance with the latest South Africa revision of the Declaration of Helsinki (Appendix 3), ICH Good Clinical Practice, and local regulatory requirements.

The investigator shall be responsible for obtaining Ethics Committee(s) approval of the protocol and any subsequent amendments in compliance with local law before the start of the trial. Copies of these approvals must be forwarded by the investigator to EMVI with the composition (names and qualification of the members) of the Ethics Committee.

9 Quality Control and Quality Assurance

9.1 Direct Access to Source Data/Documents

The principal investigator will provide direct access to the source data documents to the Ethics Committee, to the regulatory agency, and to the sponsor, permitting trial-related monitoring, audits.

9.2 Personnel Involved in the Trial

Volunteers will be included and followed by Meta Roestenberg. Dr. A. van der Ven will be responsible for the clinical supervision. Facilities of the Clinical Research Center Nijmegen (CRCN) will be used for hosting volunteers. Blood will be drawn by a research nurse from the out patient clinic of the University Medical Center Nijmegen. The research nurse will be in charge of transferring the blood samples to the hospital laboratory facilities: the CKCL and the Parasitology Laboratory, for parameter evaluation. R. Hermsen will be supervisor of the biological evaluation.

9.3 Modification of the Protocol

No amendments to this protocol will be made without consultation with, and agreement of, the sponsor and GSK Biologicals. Any amendment to the trial that appear necessary during the course of the trial must be concurrently discussed in detail with the investigator, and the sponsor and with the GSK Biologicals' Clinical Development Manager/Medical Monitor when AS02 group is involved. All parties will have a time period of 5 working days to review the amendment. Thereafter, within 10 working days, the sponsor will arrange a meeting in order to reach agreement on the amendment. Any amendment to the protocol will apply to all subjects. If agreement is reached concerning the need for an amendment, it will be produced and filed as an amendment to this protocol in writing by the sponsor and/or the investigator and will be made a formal part of the protocol. Written IRB/IEC approval of protocol

amendments is required prior to implementation; All amendments must also be transmitted to Regulatory Authorities, if applicable.

An administrative change to the protocol is one that modifies administrative and logistical aspects of a protocol but does not affect the subjects' safety, the objectives of the trial and its progress. An administrative change does not require IRB. Administrative changes are submitted to IRBs/IECs for information only.

The investigator is responsible for insuring that changes to an approved trial, during the period for which IRB approval has already been given, is not initiated without IRBs review and approval except to eliminate apparent immediate hazards to the subject.

9.4 Investigator Procedures

See Appendix 9.

9.5 Monitoring

9.5.1 Set-up Visit

A set-up visit will be performed before the inclusion of the first subject in the centre. The Monitor will verify and document that the material to be used during the trial has been received and that the investigational team has been properly informed about the trial, regulatory requirements, and the EMVI SOPs.

If the medical file is in electronic form, the Monitor will request that the investigator provide a hard copy that will be dated and signed for each follow-up visit.

9.5.2 Follow-up Visits

The Monitor will carry out regular follow-up visits. The investigator commits himself to be available for these visits and to allow the monitoring staff direct access to subject medical file and CRFs. The Monitor are committed to professional secrecy.

During the visits, the Monitor will:

carry out a quality control of trial progress: respect of protocol and operating guidelines, data collection, signature of consent forms, completion of document and appearance of SAE,
sample and product management, cold chain monitoring,
collect the CRFs and correspondent correction sheets,
assess the inclusions in order to evaluate the number of complete or on-going observations.

The Monitor will discuss any problem with the investigator and define with him the actions to be taken.

Once the CRFs corresponding to the last visit have been returned duly completed and signed, the investigator must be available to complete the correction sheets transmitted by the Monitor, if necessary, until the database is locked.

9.5.3 Close-out Visit

A close-out visit will be performed at the end of the trial. Its goals are to make sure that:

- the centre has all the documents necessary for archiving,

- all samples have been shipped,
- all unused material has been recovered,
- all products have been returned to the sponsor.

9.6 Audits and Inspections

If necessary, a quality assurance audit will be carried out by independent auditors to make sure that the trial has been conducted according to the protocol and the applicable regulations.

An inspection may be conducted by Regulatory Authorities.

The investigator must allow direct access to trial documents.

9.7 Archiving

The investigator must keep all trial documents provided by EMVI for at least 15 years after the completion or discontinuation, whatever the nature of the investigational centre (private practice, hospital, institution).

The investigator will inform EMVI of any address change.

10 Confidentiality and Publication Policy

10.1 Confidentiality

10.1.1 Confidentiality of Data

Prior to initiation of the trial, the investigators will sign a fully executed confidentiality agreement with EMVI.

The confidentiality of GSK information is defined in the Mutual Transfer Agreement signed by EMVI and the Principal Investigator.

10.1.2 Confidentiality of Patients Records

Both Parties agree to adhere to the principles of medical confidentiality in relation to Clinical Trial Subjects involved in the Clinical Trial. Personal data shall not be disclosed to EMVI or to GSK Biologicals by the Principal Investigator save where this is required directly or indirectly to satisfy the requirements of the Protocol or for the purpose of monitoring or adverse event reporting. Neither Party shall disclose the identity of Clinical Trial Subjects to third parties without prior written consent of the Clinical Trial Subject

10.2 Publications

10.2.1 Publication Policy

The final report will be prepared by the Investigator in collaboration with the Clinical Director of EMVI. It will be signed by the coordinating (or principal) investigator. The protocol and data derived from the trial are the exclusive property of EMVI.

All information provided by GSK and all data and information generated by the site as part of the study, concerning AS02A, (other than a subject's medical records) are the sole property of GSK.

All rights, title, and interests in any inventions, know-how or other intellectual or industrial property rights which are conceived or reduced to practice by site staff during the course of or as a result of the study, concerning AS02A are the sole property of GSK, and are hereby assigned to GSK.

If a written contract for the conduct of the study which includes ownership provisions inconsistent with this statement is executed between GSK and the study site, that contract's ownership provisions shall apply rather than this statement.

Any publication or presentation related to the trial must be approved by EMVI before submission of the manuscript. After publication of the results of the trial, any participating centre may publish or otherwise use its own data provided that any publication of data from the trial gives recognition to the trial group and associates EMVI, provided that EMVI will be entitled to refuse the association.

EMVI must have the opportunity to review all proposed abstracts, manuscripts or presentations regarding this trial at least 30 days prior to submission for publication/ presentation. Any information identified by EMVI as confidential must be deleted prior to submission, it being understood that the results of this trial are not to be considered confidential.

EMVI review can be expedited to meet publication guidelines.

Prior to submitting for publication, presentation, use for instructional purposes, or otherwise disclosing the study results generated by the site (collectively, a "Publication"), concerning AS02A, the investigator shall provide GSK with a copy of the proposed Publication and allow GSK a period of at least thirty (30) days [or, for abstracts, at least five (5) working days] to review the proposed Publication. Proposed Publications shall not include either GSK confidential information other than the study results or personal data on any subject, such as name or initials.

At GSK's request, the submission or other disclosure of a proposed Publication will be delayed a sufficient time to allow GSK to seek patent or similar protection of any inventions, know-how or other intellectual or industrial property rights disclosed in the proposed Publication.

10.2.2 List of Publication and Authorship

The authorized persons as an author of the publication(s) are those who have contributed to the protocol and/or to the analysis of the data, and whose names are listed on the flyleaf. According to the main topic of the publication, the first author will be the greatest contributing investigator (or biological evaluator).

11 FINANCING AND INSURANCE

11.1 Financial contract

A financial contract (reference number: 3 /2004 CLIN) has been signed between EMVI and the UMC St Rabdoud.

11.2 Adverse Events Compensation, Stipends for participation

Each volunteer will receive 550 Euro to compensate their time and travel required for participation in the trial. The volunteer will receive 275 Euro on day 140 and 275 Euro at day 365, the last day of the trial.

11.3 Insurance

EMVI Insurance Policy is covering the risks related to the investigational vaccine.

12 BIBLIOGRAPHICAL REFERENCES

WHO fact sheet No.94, revised October 1998, World Health Organization, Geneva.

Sachs J. & Malaney P. The economic and social burden of malaria, Nature. 2002 (415), 7: 680-685

Saul A, Lawrence G, Smillie A, Rzepczyk CM, Reed C, Taylor D, Anderson K, Stowers A, Kemp R, Allworth A, Anders RF, Brown GV, Pye D, Schoofs P, Irving DO, Dyer SL, Woodrow GC, Briggs WR, Reber R, Sturchler D. Human phase I vaccine trials of 3 recombinant asexual stage malaria antigens with Montanide ISA720 adjuvant. Vaccine. 1999 Aug 6;17(23-24):3145-59.

Lawrence G, Cheng QQ, Reed C, Taylor D, Stowers A, Cloonan N, Rzepczyk C, Smillie A, Anderson K, Pombo D, Allworth A, Eisen D, Anders R, Saul A. Effect of vaccination with 3 recombinant asexual-stage malaria antigens on initial growth rates of Plasmodium falciparum in non-immune volunteers. Vaccine. 2000 Mar 17;18(18):1925-31.

Genton B, Al-Yaman F, Anders R, Saul A, Brown G, Pye D, Irving DO, Briggs, WR, Mai A, Ginny M, Adiguma T, Rare L, Giddy A, Reber-Liske R, Stuerchler D, Alpers MP. Safety and immunogenicity of a three-component blood-stage malaria vaccine in adults living in an endemic area of Papua New Guinea. Vaccine. 2000 May 22;18(23):2504-11.

Edwards IR, Biriell C. Harmonisation in pharmacovigilance. Drug Saf. 1994 Feb;10(2):93-102.

Alonso PL, Sacarlal J, Aponte JJ, Leach A, Macete E et al. Efficacy of the RTS,S/AS02A vaccine against *Plasmodium falciparum* infection and disease in young African children: randomised controlled trial. *The Lancet.* 2004;364:1411-1420.

Bojang KA, Milligan PJM, Pinder M, Vigneron L, Alloueche A, Kester KE et al. Efficacy of RTS,S/AS02 Malaria Vaccine against *Plasmodium falciparum* Infection in Semi-Immune Adult Men in the Gambia: A Randomized Trial. *The Lancet.* 2001;358: 1927-34.

Kester KE, McKinney DA, Tornieporth N, Ockenhouse CF, Heppner DG, et al. Efficacy of recombinant circumsporozoite protein vaccine regimens against experimental *Plasmodium falciparum* malaria. *Journal of Infectious Diseases.* 2001; 15:183(4):640-7.

10. Walsh DS, Pichyangkul S, Gettayacamin M, et al. Safety and immunogenicity of rts,s+trap malaria vaccine, formulated in the as02a adjuvant system, in infant rhesus monkeys. Am.J.Trop.Med.Hyg. 2004;70(5):499-509.

11. Pichyangkul S, Gettayacamin M, Miller RS, et al. Pre-clinical evaluation of the malaria vaccine candidate P. falciparum MSP1(42) formulated with novel adjuvants or with alum. Vaccine 2004;22(29-30):3831-40.

13 Signatures/Investigator's Agreement to the Protocol

I have read and agree to conduct this trial according to the procedures outlined in this protocol and in accordance with local regulations and Good Clinical Practice.

Function	Name	Date	Signature
Principal Investigator	**Prof. Robert Sauerwein, MD**	8/3/05	[signature]
Investigator	**Meta Roestenberg, MD**	8/3/05	[signature]
Investigator	**André van der Ven, MD**	25/4/05	[signature]
Clinical and Regulatory Affairs Director EMVI	**Odile Leroy ,MD**	15/01/2005	[signature]

14 Appendixes

Appendix 1 : Information Sheet and Informed Consent Form

Subject Information

Malaria candidate vaccine AMA-1

Background

Malaria is caused by the *Plasmodium falciparum* parasite. It is transferred to humans by mosquitoes. In the human the parasite multiplies in red blood cells.
On a global scale, it remains the most important parasitic infection in humans and belongs to the poverty-related diseases of the world. According to WHO data more than a million deaths are caused by malaria every ear, of which 90% in Africa. Especially (young) children and pregnant women are suffering from malaria. Malaria is the cause of one fifth of the child mortality. Also the economical effects of malaria are gigantic: in Africa it costs about 12 billion US$ GNP every year, and thus limits economical development.

Controlling malaria is problematic because the range of medicine for treating malaria is very limited. Moreover, recovery is seriously hampered by the parasite's increasing immunity to the medicines used. This is why researchers all over the world are trying to develop a vaccine against malaria. The purpose is to trigger an immune reaction that could protect people, just like vaccines against, for example, the measles. Although there is no malaria vaccine on the market right now, several potential vaccine candidates are being developed. However, before they will be approved for general use, they will have to be tested on a smaller scale in a safe and secure environment.

Purpose

The most important purpose of this study is to evaluate if human beings can safely use the vaccine. For this purpose, the symptoms caused by the injections, the complaints of volunteers and the laboratory results will be evaluated. The vaccine will be tested in two doses.
Secondly, it is important to see how the human immune system reacts to the vaccine, in order to estimate its effectiveness.
To most vaccines we know, adjuvants are added, to make the vaccine more effective. As it is not yet clear which adjuvant is most effective in vaccinations against malaria and the AMA-1 vaccine, three different adjuvants will be compared in this study.
To test both the two different doses and the three different adjuvants, volunteers will be allocated to 6 different groups of 10 people at random. Every group will receive its own combination of dose and adjuvant.

DESIGN

In total 60 volunteers will be participating in this study. They will be divided into 6 groups as prescribed. Every group will receive a different dosage and adjuvant. The study will take 12 months according to the following scheme:

Visit	1*	2	3	4	5	6	7	8	9	10	11	12	13	14	15	16	17	18	19
Day	-28	0	1	3	7	14	28	29	31	35	42	56	112	113	115	119	126	140	365
Vacc.		X					X						X						
Blood	X	X			X		X			X		X	X			X		X	X

* medical screening

The trial subjects will visit the hospital 19 times in the period of 18 months.
The first visit will be a medical screening and blood sampling. This will take about two hours.
The time for the other visits will vary from 15 (blood sampling only) to 45 minutes. There will be three vaccinations in the upper arm, on days 0, 28 and 112. During 10 visits, blood samples will be taken, varying from 13 mL to 40mL per visit. Over the entire period of 12 months, a maximum of 400 mL blood will be taken. It may of course happen that an extra amount of blood needs to be taken on account of a subject’s complaints or that an additional visit will be required.

AMA-1 VACCINE

AMA-1 is a protein, part of the parasite wall. Malaria parasites use AMA-1 to enter the red blood cell. Previous research in animals showed that an immune response to AMA-1 prevents the parasite from entering the red blood cell, so that it is not be able to multiply.
Aluminum hydroxide is one of the adjuvants that will be tested in this study. It is the adjuvant that is most common in vaccines.
Montanide 720 is another adjuvant, but it has only in the last years been introduced to the market, and is now an international approved adjuvant. It has also been enrolled in several malaria vaccine trials before, which have shown reasonable results.
AS02A, at last, is an experimental adjuvant that has recently booked good results in another malaria vaccine trial in Africa.
Both Montanide 720 and AS02A have been approved for use in humans.

SAFETY

Many animal experiments have been performed with the candidate malaria vaccine AMA-1. However, it has never been tested with humans. Based on this information, the Committee of Humane Research of the region Nijmegen-Arnhem, has approved of this study.
Previous studies in different animals did not show serious side effects on vaccination with AMA-1. In combination with the adjuvants Aluminum hydroxide and Montanide 720 there may be mild side effects. Typical side effects that may occur with these adjuvants are local swelling, redness of the skin and swelling. In some very exceptional cases of experimental vaccinations, serious allergic

reactions with anaphylactic shock may occur. To evaluate any side effects a strict protocol will be followed, with availability of medical assistance 24 hours per day, 7 days a week. Study doctors will be available at telnr: 06 - 10939582
You can also contact an independent specialist if you have any questions about this study:
Dr. B.J. Kullberg telnr: 024 – 366 80 15
We also find it essential that you can be reached by phone during the study period, so that, for your own safety, we may be able to reach you if neccesary.
Also, we will inform your general practitioner of your participation in this study. If you do not agree to this, you can unfortunately not participate in this study.

CONDITIONS

To participate in this study you have to be completely healthy. Based on the questionnaire, the physical examination, the blood- and urine tests and possibly information from your family doctor it will be decided whether or not it is safe for you to participate.

HIV, HBV and HCV:
Prior to the study the candidate participants will be tested for HIV, hepatitis B and hepatitis C. We are legally obliged to inform the candidate participants about the results of the tests. People who do not want to be informed about the results of the above-mentioned tests can therefore not participate in the study.

Pregnancy:
Participants should not become pregnant during the 12-month study period. Participants are responsible for adequate contraception. The initial physical examination and medical examinations prior to each vaccination will include a pregnancy test.

Blood donation:
Participants are not allowed to donate blood during the 12-month study period.

Vaccinations:
In the 3 months preceding the start of the study and during the first 10 months of the study (13 months in total), participants should not undergo any other vaccinations. This may have consequences for those who are planning to travel abroad.

Foreign travelers:
During the first 13 months of the study, the trial subjects should not travel to countries where malaria is known to occur.
Be aware! The AMA-1 vaccine is experimental: protection from malaria has not been proven. Therefore, when travelling to malaria-endemic areas after this study the usual malaria-prophylaxis should always be used.

Other biomedical studies:
During the study period, volunteers are not allowed to participate in any other biomedical study. Volunteers that have participated in another malaria vaccine trial are excluded from this study.

RIGHTS AND DUTIES

There are no strings attached to participation in this study. You participation will be completely voluntarily and you may decide to terminate your participation at any time, without giving reasons.

Insurance:
All participants in medical studies within the UMCN are insured for negative consequences of these studies, during and after the study period. For more information, see appendix 1.

Medical information:
During and after participation your privacy will be respected. Nobody outside the trial will be notified of your participation without your approval. Both written information and examination results will be filed under code in such a way that they cannot be directly linked to you personally. Only medical researchers of this study will have access to the code. Blood and other material that belongs to you, will only be used for the tests as described in the protocol and will not be kept for longer than 5 years. Only with your permission may this material in the future be used for other tests. Blood and other material that belongs to you, will only be used to answer research questions that are in direct relation with this study. In total it will not be kept for longer than 15 years.

Payment for participation:
Trial subjects will periodically receive an inconvenience allowance. This will be 550 euro in total.

Informed consent:
If you meet the study criteria, you will be requested to sign an informed consent, in which you give your permission to study participation.

QUESTIONS?

All the information above is derived from the research protocol AMA-1. If you like, you can receive a copy of the protocol.
If, after reading the above information, you still have questions about (participating in) the AMA-1 study, you can always contact:

Drs. M. Roestenberg
Medical investigator, Medical Microbiology
Universitair Medisch Centrum Nijmegen
Tel: 024 361 95 15
Email: malariavaccin@mmb.umcn.nl

INFORMED CONSENT

To participate in:

THE AMA-1 MALARIA VACCINATION STUDY

A phase I study

- I have been informed about the study to my satisfaction. I have read the written information carefully. I have been given the opportunity to ask questions about the study, and my questions have been answered satisfactorily. I have had sufficient time to think about whether or not I will participate in the study. I have the right to withdraw my consent at any time without giving reasons.
- I agree to participation in this study.

Name:

Date of birth:

Signature: Date:

The undersigned hereby declares that the above-mentioned person had been informed about the above-mentioned study, both in writing and orally. She also declares that premature termination of the participation by the above-mentioned person shall have no influence whatsoever on the care to which he/she is entitled. Moreover, she declares to ensure the privacy of information on the above-mentioned person.

Name:

Title:

Signature: Date:

TRIAL ADVERTISING

Pertaining malaria research, the departments of Medical Microbiology and General Internal Medicine of the UMC St. Radboud are looking for:

Healthy volunteers (> 18 y/o, m/f)
For a **malaria vaccine** study

Malaria is a serious disease, of which many people die. This is why we find it important to develop a vaccine to battle malaria. These vaccines, however, have first to be tested on healthy volunteers, before they can be taken to Africa for further research.

In April 2005, the Clinical Centre for Malaria Studies a study will start at the Clinical Centre for Malaria Studies on candidate malaria vaccine AMA-1. According to strict protocol, the safety and immune reaction of the vaccine will be tested. The vaccine will be injected three times into the upper arm, whereby possible side effects will be noted. Also blood samples will be taken several times, in order to examine at the immune reaction that occurs. Before the vaccination starts, you will be medically examined. In total you will have to visit the hospital 13 times in one year.

The inconvenience allowance amounts to 550 euro's, which will be paid in phases during the study.

For further information please contact:
Meta Roestenberg, investigator: Tel 024-3619515 or 024-361111 beeper 1659
Also check www.malariavaccin.nl or email to malariavaccin@mmb.umcn.nl

Appendix 2 : Declaration of Helsinki

Ethical Principles for Medical Research Involving Human Subjects

Adopted by the 18th WMA General Assembly
Helsinki, Finland, June 1964
and amended by the
29th WMA General Assembly, Tokyo, Japan, October 1975
35th WMA General Assembly, Venice, Italy, October 1983
41st WMA General Assembly, Hong Kong, September 1989
48th WMA General Assembly, Somerset West, Republic of South Africa, October 1996
and the
52nd WMA General Assembly, Edinburgh, Scotland, October 2000
Note of Clarification on Paragraph 29 added by the WMA General Assembly, Washington 2002

A. INTRODUCTION

1.
The World Medical Association has developed the Declaration of Helsinki as a statement of ethical principles to provide guidance to physicians and other participants in medical research involving human subjects. Medical research involving human subjects includes research on identifiable human material or identifiable data.
2.
It is the duty of the physician to promote and safeguard the health of the people. The physician's knowledge and conscience are dedicated to the fulfillment of this duty.
3.
The Declaration of Geneva of the World Medical Association binds the physician with the words, "The health of my patient will be my first consideration," and the International Code of Medical Ethics declares that, "A physician shall act only in the patient's interest when providing medical care which might have the effect of weakening the physical and mental condition of the patient."
4.
Medical progress is based on research which ultimately must rest in part on experimentation involving human subjects.
5.
In medical research on human subjects, considerations related to the well-being of the human subject should take precedence over the interests of science and society.
6.
The primary purpose of medical research involving human subjects is to improve prophylactic, diagnostic and therapeutic procedures and the understanding of the aetiology and pathogenesis of disease. Even the best proven prophylactic, diagnostic, and therapeutic methods must continuously be challenged through research for their effectiveness, efficiency, accessibility and quality.
7.
In current medical practice and in medical research, most prophylactic, diagnostic and therapeutic procedures involve risks and burdens.
8.
Medical research is subject to ethical standards that promote respect for all human beings and protect their health and rights. Some research populations are vulnerable and need special protection. The particular needs of the economically and medically disadvantaged must be recognized. Special attention is also required for those who cannot give or refuse consent for themselves, for those who may be subject to giving consent under duress, for those who will not benefit personally from the research and for those for whom the research is combined with care.
9.
Research Investigators should be aware of the ethical, legal and regulatory requirements for research on human subjects in their own countries as well as applicable international requirements. No national ethical, legal or

regulatory requirement should be allowed to reduce or eliminate any of the protections for human subjects set forth in this Declaration.

B. BASIC PRINCIPLES FOR ALL MEDICAL RESEARCH

10.
It is the duty of the physician in medical research to protect the life, health, privacy, and dignity of the human subject.
11.
Medical research involving human subjects must conform to generally accepted scientific principles, be based on a thorough knowledge of the scientific literature, other relevant sources of information, and on adequate laboratory and, where appropriate, animal experimentation.
12.
Appropriate caution must be exercised in the conduct of research which may affect the environment, and the welfare of animals used for research must be respected.
13.
The design and performance of each experimental procedure involving human subjects should be clearly formulated in an experimental protocol. This protocol should be submitted for consideration, comment, guidance, and where appropriate, approval to a specially appointed ethical review committee, which must be independent of the investigator, the sponsor or any other kind of undue influence. This independent committee should be in conformity with the laws and regulations of the country in which the research experiment is performed. The committee has the right to monitor ongoing trials. The researcher has the obligation to provide monitoring information to the committee, especially any serious adverse events. The researcher should also submit to the committee, for review, information regarding funding, sponsors, institutional affiliations, other potential conflicts of interest and incentives for subjects.
14.
The research protocol should always contain a statement of the ethical considerations involved and should indicate that there is compliance with the principles enunciated in this Declaration.
15.
Medical research involving human subjects should be conducted only by scientifically qualified persons and under the supervision of a clinically competent medical person. The responsibility for the human subject must always rest with a medically qualified person and never rest on the subject of the research, even though the subject has given consent.
16.
Every medical research project involving human subjects should be preceded by careful assessment of predictable risks and burdens in comparison with foreseeable benefits to the subject or to others. This does not preclude the participation of healthy volunteers in medical research. The design of all studies should be publicly available.
17.
Physicians should abstain from engaging in research projects involving human subjects unless they are confident that the risks involved have been adequately assessed and can be satisfactorily managed. Physicians should cease any investigation if the risks are found to outweigh the potential benefits or if there is conclusive proof of positive and beneficial results.
18.
Medical research involving human subjects should only be conducted if the importance of the objective outweighs the inherent risks and burdens to the subject. This is especially important when the human subjects are healthy volunteers.
19.
Medical research is only justified if there is a reasonable likelihood that the populations in which the research is carried out stand to benefit from the results of the research.
20.
The subjects must be volunteers and informed participants in the research project.
21.

The right of research subjects to safeguard their integrity must always be respected. Every precaution should be taken to respect the privacy of the subject, the confidentiality of the patient's information and to minimize the impact of the study on the subject's physical and mental integrity and on the personality of the subject.
22.
In any research on human beings, each potential subject must be adequately informed of the aims, methods, sources of funding, any possible conflicts of interest, institutional affiliations of the researcher, the anticipated benefits and potential risks of the study and the discomfort it may entail. The subject should be informed of the right to abstain from participation in the study or to withdraw consent to participate at any time without reprisal. After ensuring that the subject has understood the information, the physician should then obtain the subject's freely-given informed consent, preferably in writing. If the consent cannot be obtained in writing, the non-written consent must be formally documented and witnessed.
23.
When obtaining informed consent for the research project the physician should be particularly cautious if the subject is in a dependent relationship with the physician or may consent under duress. In that case the informed consent should be obtained by a well-informed physician who is not engaged in the investigation and who is completely independent of this relationship.
24.
For a research subject who is legally incompetent, physically or mentally incapable of giving consent or is a legally incompetent minor, the investigator must obtain informed consent from the legally authorized representative in accordance with applicable law. These groups should not be included in research unless the research is necessary to promote the health of the population represented and this research cannot instead be performed on legally competent persons.
25.
When a subject deemed legally incompetent, such as a minor child, is able to give assent to decisions about participation in research, the investigator must obtain that assent in addition to the consent of the legally authorized representative.
26.
Research on individuals from whom it is not possible to obtain consent, including proxy or advance consent, should be done only if the physical/mental condition that prevents obtaining informed consent is a necessary characteristic of the research population. The specific reasons for involving research subjects with a condition that renders them unable to give informed consent should be stated in the experimental protocol for consideration and approval of the review committee. The protocol should state that consent to remain in the research should be obtained as soon as possible from the individual or a legally authorized surrogate.
27.
Both authors and publishers have ethical obligations. In publication of the results of research, the investigators are obliged to preserve the accuracy of the results. Negative as well as positive results should be published or otherwise publicly available. Sources of funding, institutional affiliations and any possible conflicts of interest should be declared in the publication. Reports of experimentation not in accordance with the principles laid down in this Declaration should not be accepted for publication.

C. ADDITIONAL PRINCIPLES FOR MEDICAL RESEARCH COMBINED WITH MEDICAL CARE

28.
The physician may combine medical research with medical care, only to the extent that the research is justified by its potential prophylactic, diagnostic or therapeutic value. When medical research is combined with medical care, additional standards apply to protect the patients who are research subjects.
29.
The benefits, risks, burdens and effectiveness of a new method should be tested against those of the best current prophylactic, diagnostic, and therapeutic methods. This does not exclude the use of placebo, or no treatment, in studies where no proven prophylactic, diagnostic or therapeutic method exists. See footnote
30.
At the conclusion of the study, every patient entered into the study should be assured of access to the best proven prophylactic, diagnostic and therapeutic methods identified by the study.

31.
The physician should fully inform the patient which aspects of the care are related to the research. The refusal of a patient to participate in a study must never interfere with the patient-physician relationship.
32.
In the treatment of a patient, where proven prophylactic, diagnostic and therapeutic methods do not exist or have been ineffective, the physician, with informed consent from the patient, must be free to use unproven or new prophylactic, diagnostic and therapeutic measures, if in the physician's judgement it offers hope of saving life, re-establishing health or alleviating suffering. Where possible, these measures should be made the object of research, designed to evaluate their safety and efficacy. In all cases, new information should be recorded and, where appropriate, published. The other relevant guidelines of this Declaration should be followed.

FOOTNOTE: NOTE OF CLARIFICATION ON PARAGRAPH 29 of the WMA DECLARATION OF HELSINKI
The WMA hereby reaffirms its position that extreme care must be taken in making use of a placebo-controlled trial and that in general this methodology should only be used in the absence of existing proven therapy. However, a placebo-controlled trial may be ethically acceptable, even if proven therapy is available, under the following circumstances:

- Where for compelling and scientifically sound methodological reasons its use is necessary to determine the efficacy or safety of a prophylactic, diagnostic or therapeutic method; or

- Where a prophylactic, diagnostic or therapeutic method is being investigated for a minor condition and the patients who receive placebo will not be subject to any additional risk of serious or irreversible harm.

All other provisions of the Declaration of Helsinki must be adhered to, especially the need for appropriate ethical and scientific review.
Page back to paragraph 29.

The Declaration of Helsinki (Document 17.C) is an official policy document of the World Medical Association, the global representative body for physicians. It was first adopted in 1964 (Helsinki, Finland) and revised in 1975 (Tokyo, Japan), 1983 (Venice, Italy), 1989 (Hong Kong), 1996 (Somerset-West, South Africa) and 2000 (Edinburgh, Scotland). Note of clarification on Paragraph 29 added by the WMA General Assembly, Washington 2002.

Appendix 3 : Severity Scales

Adverse Event	Intensity grade	Parameter
Pain at injection site	0	Absent
	1	Painful on touch
	2	Painful when limb is moved
	3	Pain that prevents normal activity
Redness at injection site		Record greatest surface diameter in mm
Swelling at injection site		Record greatest surface diameter in mm
Fever*		Record temperature in °C / °F
Myalgia	0	Normal
	1	Myalgia that is easily tolerated
	2	Myalgia that interferes with normal activity
	3	Myalgia that prevents normal activity
Joint pain	0	Normal
	1	Joint pain that is easily tolerated
	2	Joint pain that interferes with normal activity
	3	Joint pain that prevents normal activity
Malaise	0	Normal
	1	Malaise that is easily tolerated
	2	Malaise that interferes with normal activity
	3	Malaise that prevents normal activity
Headache	0	Normal
	1	Headache that is easily tolerated
	2	Headache that interferes with normal activity
	3	Headache that prevents normal activity
Fatigue	0	Normal
	1	Fatigue that is easily tolerated
	2	Fatigue that interferes with normal activity
	3	Fatigue that prevents normal activity
Gastrointestinal symptoms (nausea, vomiting, diarrhoea and/or abdominal pain)	0	Gastrointestinal symptoms normal
	1	Gastrointestinal symptoms that are easily tolerated
	2	Gastrointestinal symptoms that interfere with normal activity
	3	Gastrointestinal symptoms that prevent normal activity

*Fever is defined as oral temperature ≥37.5°C (99.5°F)

Redness at injection site	0	0 mm
	1	> 0 - 20 mm
	2	> 20 - 50 mm
	3	> 50 mm
Swelling at injection site	0	0 mm
	1	> 0 - 20 mm
	2	> 20 - 50 mm
	3	> 50 mm
Fever	0	< 37.5°C
	1	37.5 - 38.0°C
	2	> 38.0 to 39.0°C
	3	> 39.0°C or 102.2°F

The investigator will make an assessment of intensity for all other AEs, i.e. unsolicited symptoms, including SAEs reported during the study. The assessment will be based on the investigator's clinical judgement. The intensity of each AE and SAE recorded in the CRF or SAE Report Form, as applicable, should be assigned to one of the following categories:

1 (mild) = An AE which is easily tolerated by the subject, causing minimal discomfort and not interfering with everyday activities.

2 (moderate) = An AE which is sufficiently discomforting to interfere with normal everyday activities.

3 (severe) = An AE which prevents normal, everyday activities. (In a young child, such an AE would, for example, prevent attendance at school/ kindergarten/ a day-care centre and would cause the parents/ guardians to seek medical advice. In adults/ adolescents, such an AE would, for example, prevent attendance at work/ school and would necessitate the administration of corrective therapy.)

An AE that is assessed as Grade 3 (severe) should not be confused with a SAE. Grade 3 is a category utilized for rating the intensity of an event; and both AEs and SAEs can be assessed as Grade 3. An event is defined as 'serious' when it meets one of the pre-defined outcomes as described in Section 6.1.2

Appendix 4 : Investigator's Responsibilities

GUIDELINE FOR GOOD CLINICAL PRACTICE : CPMP/ICH/135/95/Step5, Explanatory Note and Comments to the above, issued as CPMP/768/97

4. INVESTIGATOR

4.1 Investigator's Qualifications and Agreements

4.1.1 The investigator(s) should be qualified by education, training, and experience to assume responsibility for the proper conduct of the trial, should meet all the qualifications specified by the applicable regulatory requirement(s), and should provide evidence of such qualifications through up-to-date curriculum vitae and/or other relevant documentation requested by the sponsor, the IRB/IEC, and/or the regulatory authority(ies).

4.1.2 The investigator should be thoroughly familiar with the appropriate use of the investigational product(s), as described in the protocol, in the current Investigator's Brochure, in the product information and in other information sources provided by the sponsor.

4.1.3 The investigator should be aware of, and should comply with, GCP and the applicable regulatory requirements.

4.1.4 The investigator/institution should permit monitoring and auditing by the sponsor, and inspection by the appropriate regulatory authority(ies).

4.1.5 The investigator should maintain a list of appropriately qualified persons to whom the investigator has delegated significant trial-related duties.

4.2 Adequate Resources

4.2.1 The investigator should be able to demonstrate (e.g., based on retrospective data) a potential for recruiting the required number of suitable subjects within the agreed recruitment period.

4.2.2 The investigator should have sufficient time to properly conduct and complete the trial within the agreed trial period.

4.2.3 The investigator should have available an adequate number of qualified staff and adequate facilities for the foreseen duration of the trial to conduct the trial properly and safely.

4.2.4 The investigator should ensure that all persons assisting with the trial are adequately informed about the protocol, the investigational product(s), and their trial-related duties and functions.

4.3 Medical Care of Trial Subjects

4.3.1 A qualified physician (or dentist, when appropriate), who is an investigator or a sub-investigator for the trial, should be responsible for all trial-related medical (or dental) decisions.

4.3.2 During and following a subject's participation in a trial, the investigator/institution should ensure that adequate medical care is provided to a subject for any adverse events, including clinically significant laboratory values, related to the trial. The investigator/institution should inform a subject when medical care is needed for intercurrent illness(es) of which the investigator becomes aware.

4.3.3 It is recommended that the investigator inform the subject's primary physician about the subject's participation in the trial if the subject has a primary physician and if the subject agrees to the primary physician being informed.

4.3.4 Although a subject is not obliged to give his/her reason(s) for withdrawing prematurely from a trial, the investigator should make a reasonable effort to ascertain the reason(s), while fully respecting the subject's rights.

4.4 Communication with IRB/IEC

4.4.1 Before initiating a trial, the investigator/institution should have written and dated approval/favourable opinion from the IRB/IEC for the trial protocol, written informed consent form, consent form updates, subject recruitment procedures (e.g., advertisements), and any other written information to be provided to subjects.

4.4.2 As part of the investigator's/institution’s written application to the IRB/IEC, the investigator/institution should provide the IRB/IEC with a current copy of the Investigator's Brochure. If the Investigator's Brochure is updated during the trial, the investigator/institution should supply a copy of the updated Investigator’s Brochure to the IRB/IEC.

4.4.3 During the trial the investigator/institution should provide to the IRB/IEC all documents subject to review.

4.5 Compliance with Protocol

4.5.1 The investigator/institution should conduct the trial in compliance with the protocol agreed to by the sponsor and, if required, by the regulatory authority(ies) and which was given approval/favourable opinion by the IRB/IEC. The investigator/institution and the sponsor should sign the protocol, or an alternative contract, to confirm agreement.

4.5.2 The investigator should not implement any deviation from, or changes of the protocol without agreement by the sponsor and prior review and documented approval/favourable opinion from the IRB/IEC of an amendment, except where necessary to eliminate an immediate hazard(s) to trial subjects, or when the change(s) involves only logistical or administrative aspects of the trial (e.g., change in monitor(s), change of telephone number(s)).

4.5.3 The investigator, or person designated by the investigator, should document and explain any deviation from the approved protocol.

4.5.4 The investigator may implement a deviation from, or a change of, the protocol to eliminate an immediate hazard(s) to trial subjects without prior IRB/IEC approval/favourable opinion. As soon as possible, the implemented deviation or change, the reasons for it, and, if appropriate, the proposed protocol amendment(s) should be submitted:
(a) to the IRB/IEC for review and approval/favourable opinion,
(b) to the sponsor for agreement and, if required,
(c) to the regulatory authority(ies).

4.6 Investigational Product(s)

4.6.1 Responsibility for investigational product(s) accountability at the trial site(s) rests with the investigator/institution.

4.6.2 Where allowed/required, the investigator/institution may/should assign some or all of the investigator's/institution's duties for investigational product(s) accountability at the trial site(s) to an appropriate pharmacist or another appropriate individual who is under the supervision of the investigator/institution..

4.6.3 The investigator/institution and/or a pharmacist or other appropriate individual, who is designated by the investigator/institution, should maintain records of the product's delivery to the trial site, the inventory at the site, the use by each subject, and the return to the sponsor or alternative disposition of unused product(s). These records should include dates, quantities, batch/serial numbers, expiration dates (if applicable), and the unique code numbers assigned to the investigational product(s) and trial subjects. Investigators should maintain records that document adequately that the subjects were provided the doses specified by the protocol and reconcile all investigational product(s) received from the sponsor.

4.6.4 The investigational product(s) should be stored as specified by the sponsor (see 5.13.2 and 5.14.3) and in accordance with applicable regulatory requirement(s).4.6.5 The investigator should ensure that the investigational product(s) are used only in accordance with the approved protocol.

4.6.6 The investigator, or a person designated by the investigator/institution, should explain the correct use of the investigational product(s) to each subject and should check, at intervals appropriate for the trial, that each subject is following the instructions properly.

4.7 Randomization Procedures and Unblinding
The investigator should follow the trial's randomization procedures, if any, and should ensure that the code is broken only in accordance with the protocol. If the trial is blinded, the investigator should promptly document and explain to the sponsor any premature unblinding (e.g., accidental unblinding, unblinding due to a serious adverse event) of the investigational product(s).

4.8 Informed Consent of Trial Subjects

4.8.1 In obtaining and documenting informed consent, the investigator should comply with the applicable regulatory requirement(s), and should adhere to GCP and to the ethical principles that have their origin in the Declaration of Helsinki. Prior to the beginning of the trial, the investigator should have the IRB/IEC's written approval/favourable opinion of the written informed consent form and any other written information to be provided to subjects.

4.8.2 The written informed consent form and any other written information to be provided to subjects should be revised whenever important new information becomes available that may be relevant to the subject's consent. Any revised written informed consent form, and written information should receive the IRB/IEC's approval/favourable opinion in advance of use. The subject or the subject's legally acceptable representative should be informed in a timely manner if new information becomes available that may be relevant to the subject's willingness to continue participation in the trial. The communication of this information should be documented.

4.8.3 Neither the investigator, nor the trial staff, should coerce or unduly influence a subject to participate or to continue to participate in a trial.

4.8.4 None of the oral and written information concerning the trial, including the written informed consent form, should contain any language that causes the subject or the subject's legally acceptable representative to waive or to appear to waive any legal rights, or that releases or appears to release the investigator, the institution, the sponsor, or their agents from liability for negligence.

4.8.5 The investigator, or a person designated by the investigator, should fully inform the subject or, if the subject is unable to provide informed consent, the subject's legally acceptable representative, of all pertinent aspects of the trial including the written information and the approval/ favourable opinion by the IRB/IEC.

4.8.6 The language used in the oral and written information about the trial, including the written informed consent form, should be as non-technical as practical and should be understandable to the subject or the subject's legally acceptable representative and the impartial witness, where applicable.

4.8.7 Before informed consent may be obtained, the investigator, or a person designated by the investigator, should provide the subject or the subject's legally acceptable representative ample time and opportunity to inquire about details of the trial and to decide whether or not to participate in the trial. All questions about the trial should be answered to the satisfaction of the subject or the subject's legally acceptable representative.

4.8.8 Prior to a subject’s participation in the trial, the written informed consent form should be signed and personally dated by the subject or by the subject's legally acceptable representative, and by the person who conducted the informed consent discussion.

4.8.9 If a subject is unable to read or if a legally acceptable representative is unable to read, an impartial witness should be present during the entire informed consent discussion. After the written informed consent form and any other written information to be provided to subjects, is read and explained to the subject or the subject’s legally acceptable representative, and after the subject or the subject’s legally acceptable representative has orally consented to the subject’s participation in the trial and, if capable of doing so, has signed and personally dated the informed consent form, the witness should sign and personally date the consent form. By signing the consent form, the witness attests that the information in the consent form and any other written information was accurately explained to, and apparently understood by, the subject or the subject's legally acceptable representative, and that informed consent was freely given by the subject or the subject’s legally acceptable representative.

4.8.10 Both the informed consent discussion and the written informed consent form and any other written information to be provided to subjects should include explanations of the following:
(a) That the trial involves research.
(b) The purpose of the trial.
(c) The trial treatment(s) and the probability for random assignment to each treatment.
(d) The trial procedures to be followed, including all invasive procedures.
(e) The subject's responsibilities.
(f) Those aspects of the trial that are experimental.
(g) The reasonably foreseeable risks or inconveniences to the subject and, when applicable, to an embryo, fetus, or nursing infant.
(h) The reasonably expected benefits. When there is no intended clinical benefit to the subject, the subject should be made aware of this.
(i) The alternative procedure(s) or course(s) of treatment that may be available to the subject, and their important potential benefits and risks.
(j) The compensation and/or treatment available to the subject in the event of trial-related injury.
(k) The anticipated prorated payment, if any, to the subject for participating in the trial.
(l) The anticipated expenses, if any, to the subject for participating in the trial.
(m) That the subject's participation in the trial is voluntary and that the subject may refuse to participate or withdraw from the trial, at any time, without penalty or loss of benefits to which the subject is otherwise entitled.
(n) That the monitor(s), the auditor(s), the IRB/IEC, and the regulatory authority(ies) will be granted direct access to the subject's original medical records for verification of clinical trial procedures and/or data,

without violating the confidentiality of the subject, to the extent permitted by the applicable laws and regulations and that, by signing a written informed consent form, the subject or the subject's legally acceptable representative is authorizing such access.
(o) That records identifying the subject will be kept confidential and, to the extent permitted by the applicable laws and/or regulations, will not be made publicly available. If the results of the trial are published, the subject’s identity will remain confidential.
(p) That the subject or the subject's legally acceptable representative will be informed in a timely manner if information becomes available that may be relevant to the subject's willingness to continue participation in the trial.
(q) The person(s) to contact for further information regarding the trial and the rights of trial subjects, and whom to contact in the event of trial-related injury.
(r) The foreseeable circumstances and/or reasons under which the subject's participation in the trial may be terminated.
(s) The expected duration of the subject's participation in the trial.
(t) The approximate number of subjects involved in the trial.

4.8.11 Prior to participation in the trial, the subject or the subject's legally acceptable representative should receive a copy of the signed and dated written informed consent form and any other written information provided to the subjects. During a subject’s participation in the trial, the subject or the subject’s legally acceptable representative should receive a copy of the signed and dated consent form updates and a copy of any amendments to the written information provided to subjects.

4.8.12 When a clinical trial (therapeutic or non-therapeutic) includes subjects who can only be enrolled in the trial with the consent of the subject’s legally acceptable representative (e.g., minors, or patients with severe dementia), the subject should be informed about the trial to the extent compatible with the subject’s understanding and, if capable, the subject should sign and personally date the written informed consent.

4.8.13 Except as described in 4.8.14, a non-therapeutic trial (i.e. a trial in which there is no anticipated direct clinical benefit to the subject), should be conducted in subjects who personally give consent and who sign and date the written informed consent form.

4.8.14 Non-therapeutic trials may be conducted in subjects with consent of a legally acceptable representative provided the following conditions are fulfilled:
(a) The objectives of the trial can not be met by means of a trial in subjects who can give informed consent personally.
(b) The foreseeable risks to the subjects are low.
(c) The negative impact on the subject’s well-being is minimized and low.
(d) The trial is not prohibited by law.
(e) The approval/favourable opinion of the IRB/IEC is expressly sought on the inclusion of such subjects, and the written approval/ favourable opinion covers this aspect.

Such trials, unless an exception is justified, should be conducted in patients having a disease or condition for which the investigational product is intended.
Subjects in these trials should be particularly closely monitored and should be withdrawn if they appear to be unduly distressed.

4.8.15 In emergency situations, when prior consent of the subject is not possible, the consent of the subject's legally acceptable representative, if present, should be requested. When prior consent of the subject is not possible, and the subject’s legally acceptable representative is not available, enrolment of the subject should require measures described in the protocol and/or elsewhere, with documented approval/favourable opinion by the IRB/IEC, to protect the rights, safety and well-being of the subject and to ensure compliance with applicable regulatory requirements. The subject or the subject's legally

acceptable representative should be informed about the trial as soon as possible and consent to continue and other consent as appropriate (see 4.8.10) should be requested.

4.9 Records and Reports

4.9.1 The investigator should ensure the accuracy, completeness, legibility, and timeliness of the data reported to the sponsor in the CRFs and in all required reports.

4.9.2 Data reported on the CRF, that are derived from source documents, should be consistent with the source documents or the discrepancies should be explained.

4.9.3 Any change or correction to a CRF should be dated, initialed, and explained (if necessary) and should not obscure the original entry (i.e. an audit trail should be maintained); this applies to both written and electronic changes or corrections (see 5.18.4 (n)). Sponsors should provide guidance to investigators and/or the investigators' designated representatives on making such corrections. Sponsors should have written procedures to assure that changes or corrections in CRFs made by sponsor's designated representatives are documented, are necessary, and are endorsed by the investigator. The investigator should retain records of the changes and corrections.

4.9.4 The investigator/institution should maintain the trial documents as specified in Essential Documents for the Conduct of a Clinical Trial (see 8.) and as required by the applicable regulatory requirement(s). The investigator/institution should take measures to prevent accidental or premature destruction of these documents.

4.9.5 Essential documents should be retained until at least 2 years after the last approval of a marketing application in an ICH region and until there are no pending or contemplated marketing applications in an ICH region or at least 2 years have elapsed since the formal discontinuation of clinical development of the investigational product. These documents should be retained for a longer period however if required by the applicable regulatory requirements or by an agreement with the sponsor. It is the responsibility of the sponsor to inform the investigator/institution as to when these documents no longer need to be retained (see 5.5.12).

4.9.6 The financial aspects of the trial should be documented in an agreement between the sponsor and the investigator/institution.

4.9.7 Upon request of the monitor, auditor, IRB/IEC, or regulatory authority, the investigator/institution should make available for direct access all requested trial-related records.

4.10 Progress Reports

4.10.1 The investigator should submit written summaries of the trial status to the IRB/IEC annually, or more frequently, if requested by the IRB/IEC.

4.10.2 The investigator should promptly provide written reports to the sponsor, the IRB/IEC (see 3.3.8) and, where applicable, the institution on any changes significantly affecting the conduct of the trial, and/or increasing the risk to subjects.

4.11 Safety Reporting

4.11.1 All serious adverse events (SAEs) should be reported immediately to the sponsor except for those SAEs that the protocol or other document (e.g., Investigator's Brochure) identifies as not needing immediate reporting. The immediate reports should be followed promptly by detailed, written reports.

The immediate and follow-up reports should identify subjects by unique code numbers assigned to the trial subjects rather than by the subjects' names, personal identification numbers, and/or addresses. The investigator should also comply with the applicable regulatory requirement(s) related to the reporting of unexpected serious adverse drug reactions to the regulatory authority(ies) and the IRB/IEC.

4.11.2 Adverse events and/or laboratory abnormalities identified in the protocol as critical to safety evaluations should be reported to the sponsor according to the reporting requirements and within the time periods specified by the sponsor in the protocol.

4.11.3 For reported deaths, the investigator should supply the sponsor and the IRB/IEC with any additional requested information (e.g., autopsy reports and terminal medical reports).

4.12 Premature Termination or Suspension of a Trial
If the trial is prematurely terminated or suspended for any reason, the investigator/institution should promptly inform the trial subjects, should assure appropriate therapy and follow-up for the subjects, and, where required by the applicable regulatory requirement(s), should inform the regulatory authority(ies). In addition:

4.12.1 If the investigator terminates or suspends a trial without prior agreement of the sponsor, the investigator should inform the institution where applicable, and the investigator/institution should promptly inform the sponsor and the IRB/IEC, and should provide the sponsor and the IRB/IEC a detailed written explanation of the termination or suspension.

4.12.2 If the sponsor terminates or suspends a trial (see 5.21), the investigator should promptly inform the institution where applicable and the investigator/institution should promptly inform the IRB/IEC and provide the IRB/IEC a detailed written explanation of the termination or suspension.

4.12.3 If the IRB/IEC terminates or suspends its approval/favourable opinion of a trial (see 3.1.2 and 3.3.9), the investigator should inform the institution where applicable and the investigator/institution should promptly notify the sponsor and provide the sponsor with a detailed written explanation of the termination or suspension.

4.13 Final Report(s) by Investigator

Upon completion of the trial, the investigator, where applicable, should inform the institution; the investigator/institution should provide the IRB/IEC with a summary of the trial’s outcome, and the regulatory authority(ies) with any reports required.

Appendix 5 : Text of Regulatory approval

Appendix 6 : SAE Report Form

Appendix 7 : Case Report Form

Appendix 8 : Investigator SOPs

SOP VOLUNTEERS

Recruitment of volunteers:
Recruitment of volunteers for vaccination is via advertisements in local paper, via posters on notice boards and via the information page on the website of the unit. Please refer to appendix 1 for advertisement and subject information.

Phone callers are asked a list of selected questions (see below) and when seemingly suitable and interested they are sent an enrolment form for the study, study information for volunteers (appendix 1) including information on the insurance which the hospital takes out for every volunteer (appendix 1).
Enrolled applicants are invited for a medical screening, and their names and allocated subject numbers are recorded in a registration file.

Volunteers who seem unsuitable for this study but who may be eligible for studies other than AMA-1 are asked for their approval of recording their personal information in a registration file.

To volunteers who seem unsuitable for any studies, due to verbal abuse or mental illness is communicated that they unfortunately are not fit to partake in a study. Their personal information will be taken out of the registration file and destroyed.

Visit 1, medical screening:
At the medical screening the trial procedures will be explained to the volunteers. They will have the opportunity to ask questions about the trial. Points that are still unclear will be discussed.
They will also be explained about the informed consent form (appendix 1), of which they will receive a copy. Following, the investigator and volunteer will go over the questions of the enrolment form and a general physical examination will be performed. The volunteer will be asked for permission to withdraw blood samples and perform blood tests, including HIV, HBV and HCV tests. A research nurse will perform blood sampling. It will then be decided whether or not the volunteer meets the inclusion criteria and is therefore suitable for the trial.

After medical screening those who are suitable for the study are invited for an appointment to sign their informed consent and receive their first vaccination. This appointment will be approximately 28 days, but no less than 14 days after the medical examination.

Those who are unsuitable but who may be eligible for studies other than AMA-1 are asked for their approval of recording their personal information in a registration file. All information about their medical condition, physical examination and laboratory tests will be destroyed.

Visit 2:
At this appointment subjects will again be given the opportunity to ask questions about the trial. The volunteer and investigator will discuss a checklist of topics that are of greatest importance for the volunteer's understanding of the trial (see below). Subjects will then be asked to sign the informed consent form in the presence of the investigator.
Afterwards, the first vaccination will be given.

Medical care:
If any medical care might be needed, subjects will receive the appropriate medical care according to hospital procedures.

Non-compliance:

If a volunteer fails to appear for follow-up, extensive effort will be undertaken to at least determine the health status of the subject. A minimum of two emails and three attempts will be made to reach the volunteer by phone. As last resort the investigator will pay a visit to the volunteer's home address.

Checklist for phone callers:

- ☐ 1. Between 18 and 45 years old
- ☐ 2. Available for screening
- ☐ 3. Available for study
- ☐ 4. Within 30 min traveling to hospital, reachable by phone
- ☐ 5. Not pregnant, not lactating
- ☐ 6. Prepared to take pregnancy test
- ☐ 7. Prepared to undergo HIV test
- ☐ 8. Prepared to visit hospital 13 times
- ☐ 9. No chronic illness, good general health
- ☐ 10. No medication or drug use
- ☐ 11. Has not been in malaria-endemic area for past 6 months
- ☐ 12. No history of malaria infection or malaria vaccine study
- ☐ 13. Not enrolled in other trial

Checklist day 0:

- ☐ 1. Malaria background
- ☐ 2. 3 vaccinations
- ☐ 3. Possible side-effects
- ☐ 4. Pregnancy tests
- ☐ 5. 13 visits
- ☐ 6. 7 blood samples
- ☐ 7. Physical examinations
- ☐ 8. Insurance
- ☐ 9. Privacy
- ☐ 10. Payment for participation
- ☐ 11. Blood donor / pregnancy / travels abroad
