# Supplementary material for: Safety and Immunogenicity of a Recombinant Plasmodium falciparum AMA1 Malaria Vaccine Adjuvanted with Alhydrogel™, Montanide ISA 720 or AS02
Source: PLoS One. 2008 Dec 18;3(12):e3960. doi: 10.1371/journal.pone.0003960 (PMC2602972; doi:10.1371/journal.pone.0003960)
Supplement: Amendment S1 — (0.26 MB PDF) [file pone.0003960.s003.pdf]

1. Signatures

I have read the amendment and agree the trial will be conducted according to the procedures described.

Function	Name	Date	Signature
Principal Investigator	Prof Robert Sauerwein	8/3/05	
Investigator	Dr Meta Roestenberg	8/3/05	
E.M.V.I. Project manager	Dr Hildur Blythman	28/4/05	
E.M.V.I. Director of Clinical and Regulatory Affairs	Dr Odile Leroy	03/03/05	

This information is the property of EMVI and all rights are reserved by EMVI

2. Justification of the amendment

After the Ethical review, the Information to the participants has been amended by the Principal Investigator, to correct mistyping and to describe more in detail the procedure.

The paragraphs modified by this amendment are described below.
Other paragraphs remain unchanged.

This information is the property of EMVI and all rights are reserved by EMVI

PAGE 45 AND 46

Appendix 1 : Information Sheet and Informed Consent Form

Previous version :

SAFETY

Many successful animal experiments have been performed with the candidate malaria vaccine AMA-1. Based on this information, the Central Committee of Humane Research of the region Nijmegen-Arnhem, has approved of this study.
Previous studies in different animals did not show serious side effects on vaccination with AMA-1. In combination with the adjuvants Aluminum hydroxide and Montanide 720 there may be mild side effects. Typical side effects that may occur after vaccination are local swelling, redness of the skin, slight fever. In some very exceptional cases, serious allergic reactions with anaphylactic shock may occur. To evaluate any side effects a strict protocol will be followed, with availability of medical assistance 24 hours per day, 7 days a week. Study doctors will be available at telnr: 06 -
You can also contact an independent specialist if you have any questions about this study:
Dr. B.J. Kullberg telnr: 024 – 366 80 15

New version :

SAFETY

Many animal experiments have been performed with the candidate malaria vaccine AMA-1. However, it has never been tested with humans. Based on this information, the Committee of Humane Research of the region Nijmegen-Arnhem, has approved of this study.
Previous studies in different animals did not show serious side effects on vaccination with AMA-1. In combination with the adjuvants Aluminum hydroxide and Montanide 720 there may be mild side effects. Typical side effects that may occur with these adjuvants are local swelling, redness of the skin and swelling. In some very exceptional cases of experimental vaccinations, serious allergic reactions with anaphylactic shock may occur. To evaluate any side effects a strict protocol will be followed, with availability of medical assistance 24 hours per day, 7 days a week. Study doctors will be available at telnr: 06 - 10939582
You can also contact an independent specialist if you have any questions about this study:
Dr. B.J. Kullberg telnr: 024 – 366 80 15
We also find it essential that you can be reached by phone during the study period, so that, for your own safety, we may be able to reach you if neccesary.
Also, we will inform your general practitioner of your participation in this study. If you do not agree to this, you can unfortunately not participate in this study.

This information is the property of EMVI and all rights are reserved by EMVI

Previous version :

CONDITIONS

Foreign travelers:

During the first 13 months of the study, the trial subjects should not travel to countries where malaria is known to occur.

New version:

CONDITIONS

Foreign travelers:

During the first 13 months of the study, the trial subjects should not travel to countries where malaria is known to occur.
Be aware! The AMA-1 vaccine is experimental: protection from malaria has not been proven. Therefore, when travelling to malaria-endemic areas after this study the usual malaria-prophylaxis should always be used.

Previous version:

RIGHTS AND DUTIES

Medical information:

During and after participation your privacy will be respected. Nobody outside the trial will be notified of your participation without your approval. Both written information and examination results will be filed under code in such a way that they cannot be directly linked to you personally. Only medical researchers of this study will have access to the code. Blood and other material that belongs to you, will only be used for the tests as described in the protocol and will not be kept for longer than 5 years. Only with your permission may this material in the future be used for other tests.

New version:

RIGHTS AND DUTIES

Medical information:

During and after participation your privacy will be respected. Nobody outside the trial will be notified of your participation without your approval. Both written information and examination results will be filed under code in such a way that they cannot be directly linked to you personally. Only medical researchers of this study will have access to the code. Blood and other material that belongs to you, will only be used for the tests as described in the protocol and will not be kept for longer than 5 years. Only with your permission may this material in the future be used for other tests. Blood and other material that belongs to you, will only be used to answer research questions that are in direct relation with this study. In total it will not be kept for longer than 15 years.

This information is the property of EMVI and all rights are reserved by EMVI

3. Appendixes

1. Amendment List

Amendment number	Date	Protocol file name	Version	EC submission	
				Yes/ No	date
1	03/03/05	PAMA1_050303	Final_1	No	

2. Amended protocol

This information is the property of EMVI and all rights are reserved by EMVI
