# Supplementary material for: Safety and Immunogenicity of a Recombinant Plasmodium falciparum AMA1 Malaria Vaccine Adjuvanted with Alhydrogel™, Montanide ISA 720 or AS02
Source: PLoS One. 2008 Dec 18;3(12):e3960. doi: 10.1371/journal.pone.0003960 (PMC2602972; doi:10.1371/journal.pone.0003960)
Supplement: Amendment S2 — (0.42 MB PDF) [file pone.0003960.s004.pdf]

E.M.V.I.	Protocol Amendment	Trial code:	AMA-I_1_03
European Malaria Vaccine Initiative	***Title: Assessment of the Safety and Immunogenicity of three Formulations of the Recombinant Pichia pastoris Apical Membrane Antigen 1 (PfAMA-1-FVO[25-545]), Blood-stage Malaria Vaccine in Healthy Dutch Adult Volunteers : a Phase 1, Single-Blind, Randomised, Dose-escalating, Unicentre trial.***	Version No.:	**1**
Good Clinical Practices		Effective Date:	16/05/05

1. Signatures

I have read the amendment and agree that the trial will be conducted according to the procedures described.

Function	Name	Date	Signature
Principal Investigator	Prof Robert Sauerwein	24/6/05	[signature]
Investigator	Dr Meta Roestenberg	24/06/05	[signature]
E.M.V.I. Proiect manager	Dr Hildur E. Blythman	24.06.05	[signature]
E.M.V.I. Director of Clinical and Regulatory Affairs	Dr Odile Leroy	22/06/05	[signature]

This information is the property of EMVI and all rights are reserved by EMVI

2. Justification of the amendment

The paragraphs modified by this amendment are described below. Other paragraphs remain unchanged

Page 35 and 36

7.2.2. Cellular Immune response:

2. Parameters to be measured, Method and Timing of Measurement

Cytokine production will be assayed by ELISPOT for the cytokines IL-4 and IFNγ

Change to:

Cytokine production will be assayed by ELISPOT for the cytokines IL-5 and IFNγ

Justification for the change:

- The IL-4 production of Polymorphic Blood Mononuclear Cells (PBMC's) measured in the ELISPOT is low as compared to the IL-5 production. Therefore, due to the low numbers of spots, the read-out will be less reproducible and the intra-assay variability will be higher as compared to IL-5.
- ELISPOT results from this study can easily be compared with the IFNγ/IL-5 data published by researchers of GSK[1], [2], one of the co-investigators in this study.

[1] Walsh DS, Pichyangkul S, Gettayacamin M, et al. Safety and immunogenicity of rts,s+trap malaria vaccine, formulated in the as02a adjuvant system, in infant rhesus monkeys. Am.J.Trop.Med.Hyg. 2004;70(5):499-509.

[2] Pichyangkul S, Gettayacamin M, Miller RS, et al. Pre-clinical evaluation of the malaria vaccine candidate P. falciparum MSP1(42) formulated with novel adjuvants or with alum. Vaccine 2004;22(29-30):3831-40.

This information is the property of EMVI and all rights are reserved by EMVI

3. Appendixes

1. Amendment List

Amendment number	Date	Protocol file name	Version	EC submission	
				Yes/ No	date
1	03/03/05	PAMA1 050303	Final_1	No	
2	16/05/05	PAMA1 050516	Final_2		

2. Amended protocol

This information is the property of EMVI and all rights are reserved by EMVI
