# Supplementary material for: Safety and Immunogenicity of a Recombinant Plasmodium falciparum AMA1 Malaria Vaccine Adjuvanted with Alhydrogel™, Montanide ISA 720 or AS02
Source: PLoS One. 2008 Dec 18;3(12):e3960. doi: 10.1371/journal.pone.0003960 (PMC2602972; doi:10.1371/journal.pone.0003960)
Supplement: Amendment S3 — (0.30 MB PDF) [file pone.0003960.s005.pdf]

Signatures

I have read the amendments and agree that the trial will be conducted according to the procedures described.

Function	Name	Date	Signature
Principal Investigator	Prof Robert Sauerwein		
Investigator	Dr Meta Roestenberg		
E.M.V.I. Project Manager	Dr Hildur E. Blythman	24/07/05	[signature]
E.M.V.I. Director of Clinical and Regulatory Affairs	Dr Odile Leroy	22/06/05	[signature]

This information is the property of EMVI and all rights are reserved by EMVI

Amendment N° 3

The paragraphs modified by this amendment are described below. Other paragraphs remain unchanged.

11.3 Insurance

EMVI Insurance Policy is covering the risks related to the investigational vaccine.

Change to:

11.3 Insurance

EMVI Insurance Policy is covering the risks related to the investigational vaccine. To this effect, the EMVI has subscribed an Insurance with GERLING Allgemeine Versicherung AG (policy number 67 – 410118 – 16)

Insurance:
All participants in medical studies within the UMCN are insured for negative consequences of these studies, during and after the study period. For more information, see appendix 1.

Change to:

Insurance:
The sponsor, EMVI, has subscribed an Insurance to cover the "negative "consequences"of this trial, if it can be proven that these consequences are related to the trial procedure and/or test product. For more information, see Insurance text provided.

Justification for the change: For studies sponsored by external organisations, the participants are not covered by the UMCN insurance; therefore, the EMVI has to cover the medical "negative "consequences", if they can be proven to be related to the trial procedure and/or test product.

This information is the property of EMVI and all rights are reserved by EMVI

Amendment N° 4

The paragraphs modified by this amendment are described below. Other paragraphs remain unchanged

---

3.2.2 Vaccination, Clinical Follow-up and Serology schedule

The vaccination and serology schedule is summarized in the modified flow chart. Each subject will attend at 13 standardised visits at the trial centre (Inclusion visit D-28; D0; D1; D14; D28; D29; D42; D56; D112; D113; D126; D140; D365). Two doses of vaccine at 4 week-interval will be given by intra-muscular route at D0 and D28. A booster dose will be given 12 weeks after the primary series at D112.
Blood samples will be taken at ten time points at D-28; D0; D7, D28; D35, D56; D112; D119, D140, and D365. Before each vaccination and 7 and 28 days after each vaccination a venapuncture will be performed. Biological parameters are assessed at inclusion, 7 and 28 days after each vaccination and after a year. Blood for immunologic assays will be collected at inclusion, before each vaccination, 28 days after each vaccination and a year after the first immunisation. A venapuncture will be performed ten times during the course of the study. A maximum total of 40 mL blood will be taken at each blood sampling. For each subject a maximum of 400 mL blood will be taken during the whole study.
Twenty four hours, 3 days, 7 days and 14 days after each vaccination a visit is planned to monitor adverse events at D1, D3, D7, ,D14, D29, D31, D35, D42, D113, D114, D119 and D126.

Change to:

3.2.2 Vaccination, Clinical Follow-up and Serology schedule

The vaccination and serology schedule is summarized in the flow chart. Each subject will attend at 19 standardised visits at the trial centre (Inclusion visit D-28; D0; D1; D3; D7; D14; D28; D29; D31; D35; D42; D56; D57; D59; D63; D70; D84; D140; D365). Three doses of vaccine at 4 week-interval will be given by intra-muscular route at D0, D28 and D56.
Blood samples will be taken at ten time points at D-28; D0; D7, D28; D35, D56; D63; D84, D140, and D365. Before each vaccination and 7 and 28 days after each vaccination a venapuncture will be performed. Biological parameters are assessed at inclusion, 7 and 28 days after each vaccination and 2 months post-3rd vaccination (D140) and 1 year after 1st. vaccination (D365). Blood for immunologic assays will be collected at inclusion, before each vaccination, 28 days after each vaccination, 2 months post-3rd vaccination and a year after the first immunisation. A maximum total of 40 mL blood will be taken at each blood sampling. For each subject a maximum of 400 mL blood will be taken during the whole study.
Twenty four hours, 3 days, 7 days and 14 days after each vaccination a visit is planned to monitor adverse events at D1, D3, D7, D14, D29, D31, D35, D42, D57, D59, D63 and D70.

<u>Justification for the change</u>:

This information is the property of EMVI and all rights are reserved by EMVI

1. for internal logistics reasons, the trial will start as planned, but only with the groups receiving the vaccine containing either Alum or Montanide ISA720; the group of subjects that will receive the vaccine containing AS02A (cf Chapter 3, page 12) will be enrolled later.

2. the vaccination schedule will be modified as follows: the 3 vaccination doses will be administered at a 4-week interval, to conform to the schedule recommended by the EPI (Expanded Programme on Immunisation); this has been decided, since the present vaccine's ultimate target populations are found in the malaria endemic areas worldwide.

7.4.3 Data Set to be analysed

- subjects who have respected the number of vaccination and the time-intervals between the vaccination:
  - V2 : 1st vaccination: DO, interval V1+4 weeks (± 7days)
  - V7 : 2nd vaccination D28 , interval V2+4 weeks (± 2 days)
  - **V13: 3rd vaccination D112 , interval V5+12 weeks (± 7days)**
- Subjects having respected the number of blood samples for biological safety evaluation, as well as the intervals between blood samples :
  - V1, V2, V5, V7, V10, V11, V12, V16, V18 and V19 samples at D-28, D0, D28, D56, and D140, interval 4 weeks (± 2 days) with the preceding sample
  - **V9, sample at D112, interval 12 weeks (± 7days) with the preceding sample**.

Change to:

7.4.3 Data Set to be analysed

- subjects who have respected the number of vaccination and the time-intervals between the vaccination:
  - V2 : 1st vaccination: DO, interval V1+4 weeks (± 7days)
  - V7 : 2nd vaccination D28 , interval V2+4 weeks (± 2 days)
  - V13: 3rd vaccination D56 , interval V7+4 weeks (± 2days)
- Subjects having respected the number of blood samples for biological safety evaluation, as well as the intervals between blood samples :
  - V1, V2, V5, V7, V10, V12, V15, V17, V18 and V19 samples and intervals specified in the flowchart.

Justification for the change: See above.

Appendix 1 : Information Sheet and Informed Consent Form

DESIGN

This information is the property of EMVI and all rights are reserved by EMVI

In total 60 volunteers will be participating in this study. They will be divided into 6 groups as prescribed. Every group will receive a different dosage and adjuvant. The study will take 12 months according to the following scheme:

Visit	1*	2	3	4	5	6	7	8	9	10	11	12	13	14	15	16	17	18	19
Day	-28	0	1	3	7	14	28	29	31	35	42	56	112	113	115	119	126	140	365
Vacc.		X					X						X						
Blood	X	X			X		X			X		X	X			X		X	X

* medical screening

The trial subjects will visit the hospital 19 times in the period of 18 months.
The first visit will be a medical screening and blood sampling. This will take about two hours.
The time for the other visits will vary from 15 (blood sampling only) to 45 minutes. There will be three vaccinations in the upper arm, on days 0, 28 and 112. During 10 visits, blood samples will be taken, varying from 13 mL to 40mL per visit. Over the entire period of 12 months, a maximum of 400 mL blood will be taken. It may of course happen that an extra amount of blood needs to be taken on account of a subject's complaints or that an additional visit will be required.

Change to :

Appendix 2 : Information Sheet and Informed Consent Form

DESIGN

In total 60 volunteers will be participating in this study. They will be divided into 6 groups as prescribed. Every group will receive a different dosage and adjuvant. The study will take 12 months according to the following scheme:

Visit	1*	2	3	4	5	6	7	8	9	10	11	12	13	14	15	16	17	18	19
Day	-28	0	1	3	7	14	28	29	31	35	42	56	57	59	63	70	84	140	365
Vacc.		X					X					X							
Blood	X	X			X		X			X		X			X		X	X	X

* medical screening

The trial subjects will visit the hospital 19 times in the period of 12 months.
The first visit will be a medical screening and blood sampling. This will take about two hours.
The time for the other visits will vary from 15 (blood sampling only) to 45 minutes. There will be three vaccinations in the upper arm, on days 0, 28 and 56. During 10 visits, blood samples will be taken, varying

This information is the property of EMVI and all rights are reserved by EMVI

from 13 mL to 40mL per visit. Over the entire period of 12 months, a maximum of 400 mL blood will be taken. It may of course happen that an extra amount of blood needs to be taken on account of a subject's complaints or that an additional visit will be required.

Justification for the change: See above.

This information is the property of EMVI and all rights are reserved by EMVI

E.M.V.I.	Protocol Amendment	Trial code:	AMA-1_1_03
European Malaria Vaccine Initiative	*Title: Assessment of the Safety and Immunogenicity of three Formu1ations of the Recombinant Picbia pastoris Apical Membrane Antigen 1 (PfAMA-l-FVO[25-45J), Blood-stage Malaria Vaccine in Healthy Dutch Adult Volunteers : a Phase 1, Single-Blind, Randomised, Dose-escalating, Unicentre triaI.*	Version No.:	1
Good Clinical Practices		Effective Date:	21/06/05

Prime & Boost phase, 19 Visits, 3 Vaccinations, Randomised, Dose Escalating, 365 Days duration/subject

Visit Number	V1	V2	V3	V4	V5	V6	V7	V8	V9	V10	V11	V12	V13	V14	V15	V16	V17	V18	V19
Trial Timelines (Days,)	D-28	D0	D1	D3	D7	D14	D28	D29	D31	D35	D42	D56	D57	D59	D63	D70	D84	D140	D365
Time Windows (Days or Hours)		± 7 D	± 6 hrs	± 24 hrs	± 24 hrs	± 2 D	± 2 D	± 6 hrs	± 24hrs	± 24 hrs	± 2 D	± 2 D	±6 hs	± 24 hrs	± 24hrs	± 2 D	± 2 D	± 7D	± 14 D
Visit Intervals		V1 +4 wks	V2 +24 hrs	V2 + 3D	V2 + 7D	V2 +2 wks	V2 + 4 wks	V7 + 24hrs	V7 + 3D	V7 + 7D	V7 +2 wks	V7 + 4 wks	V12 + 24hrs	V12 + 3D	V12 + 7D	V12 + 2 wks	V12 +4 wks	V17 +8 wks	V1 +1 year
Vaccination Doses		Vac1					Vac2					Vac3							
Eligibility Criteria	X																		
HIV, HCV, HBV tests	X																		
In- & Non-Inclusion Criteria		X																	
Informed Consent	X	X																	
Medical History	X																		
Physical Examination	X	X	X	X	X	X	X	X	X	X	X	X	X	X	X	X	X	X	X
Contra- Indications Review		X					X					X							
Pregnancy Test	X	X					X					X							
Immediate surveillance (30 min)		X					X					X							
Solicited Events			X	X	X	X		X	X	X	X		X	X	X	X	X		
Unsolicited Events		X	X	X	X	X		X	X	X	X		X	X	X	X	X	X	X
Serious adverse events	To be reported at any time during the trial																		
Prior and concomitant therapy		X					X					X							
Blood Sampling	BS1	BS2			BS3		BS4			BS5		BS6			B27		BS8	BS9	BS10
AMA-1 IgG, IgG isotypes, IgM		X					X					X					X	X	X
GIA; t-cell proliferation		X										X					X		
IFNγ, ELISPOT		X										X					X		
IFA		X															X		
Termination record / interim												X						X	

This information is the property of EMVI and all rights are reserved by EMVI

**Visit Number**	**V1**	**V2**	**V3**	**V4**	**V5**	**V6**	**V7**	**V8**	**V9**	**V10**	**V11**	**V12**	**V13**	**V14**	**V15**	**V16**	**V17**	**V18**	**V19**
**Trial Timelines (Days,)**	**D-28**	**D0**	**D1**	**D3**	**D7**	**D14**	**D28**	**D29**	**D31**	**D35**	**D42**	**D56**	**D57**	**D59**	**D63**	**D70**	**D84**	**D140**	**D365**
**Time Windows (Days or Hours)**		± 7 D	± 6 hrs	± 24 hrs	± 24 hrs	± 2 D	± 2 D	± 6 hrs	± 24hrs	± 24 hrs	± 2 D	± 2 D	±6 hs	± 24 hrs	± 24hrs	± 2 D	± 2 D	± 7D	± 14 D
**Visit Intervals**		V1 +4 wks	V2 +24 hrs	V2 + 3D	V2 + 7D	V2 +2 wks	V2 + 4 wks	V7 + 24hrs	V7 + 3D	V7 + 7D	V7 +2 wks	V7 + 4 wks	V12 + 24hrs	V12 + 3D	V12 + 7D	V12 + 2 wks	V12 +4 wks	V17 +8 wks	V1 +1 year
**Vaccination Doses**		**Vac1**					**Vac2**					**Vac3**							
**Termination record / Final**																			X
**Diary card provided**		DC1					DC2					DC3							
**Diary card collected**						DC1					DC2					DC3			
**Case Report Form Sections**	CRFA						CRFB					CRFC					CRFD		

This information is the property of EMVI and all rights are reserved by EMVI

Amendment N° 5

The paragraphs modified by this amendment are described below. Other paragraphs remain unchanged.

Text to be added to the Informed Consent:

Information on Test Subjects and Declaration Of Consent Regarding the VIP Check System.

The Nijmegen Clinical Research Centre (CRCN) is linked to the VIP Check network. VIP Check is an abbreviation of "Volunteer Inclusion Period" Check. This means that test subjects are checked for availability for subsequent participation in research. The VIP Check system is used during the CRCN research to which you have subscribed.

VIP Check is an international database that is based in Freiburg (Germany) and was established in 1986 by the "Ethik-Kommission International" (International Ethics Commission). VIP Check is a computerised system that consists of a central database with links to local databases in several research institutions. The main purpose of VIP Check is to maintain the safety of test subjects.

When you participate as a test subject in drug research at one of the research institutions linked to the VIP Check network, certain information on you is entered into the VIP Check system. This information includes the following:

- the first four letters of your surname
- your date of birth
- your place of birth
- the date (in the period of the study) on which the test subject was last given medicine.

This information is entered by an appropriately authorised employee of the research institution, and the system then converts it into a unique code. Each research institution always uses the same personal data and therefore the unique code of the relevant test subject will always be the same. The unique code is used to register participation in drug research in Freiburg. Through the use of this code, the privacy of test subjects is maintained. Due to the importance that all participating research institutions use the same identification data to generate the unique code, the required information is taken from your identification documents (preferably a Passport or extract from the Municipal Register, if you do not possess this: your ID card, if you do not possess this: your Driver's Licence, if you do not possess any of the documents mentioned above: your Residency Permit). For this reason, you must take your identification documents with you when you make your first visit to the CRCN. A copy of this document will be kept for 15 years in a secure location at the CRCN.

This information is the property of EMVI and all rights are reserved by EMVI

When you register again at a later date for further participation in drug research at the CRCN or at any other research institution that uses the VIP Check system, the following criteria can be checked:

- Test subjects are permitted to participate in a maximum of four drug research studies per year
- A period of 90 days must have passed between studies since the last day medication was administered (unless otherwise mentioned in the protocol).

INFORMED CONSENT FOR USING THE VIP CHECK SYSTEM

Surname and initials of the test subject:...

Date of birth [ddmmjj] : ....

I hereby declare and understand that the first four letters of my surname, date of birth, place of birth and the most recent date of taking a drug under research will be entered, on an anonymous basis, into an international database and checked. This is done in order to ensure that I have not participated in more than four drug research studies this year and that I have not participated in drug research studies in the past three months. For this purpose my identification document may be copied and kept at the CRCN.

Signature of the test subject:

Date:

Place:

I hereby declare that I, in my capacity of research doctor, have verbally explained the purpose and work method of the VIP Check system.

Signature of the research doctor

Date:

Place:

Justification for the change: The CRCN (Clinical Research Center Nijmegen) follows the recommendations of the International Ethics Committee, to ensure that the volunteers participating in the trial are not (or were not) enrolled in another trial.

This information is the property of EMVI and all rights are reserved by EMVI

Appendixes

1. Amendment List

Amendment number	Date	Protocol file name	Version	EC submission	
				Yes/ No	date
1	03/03/05	PAMA1_050303	Final_1	No	
2	16/05/05	PAMAl_050516	Final_2		
3	21/06/05	PAMAl_050516	Final_2		
4	21/06/05	PAMAl_050516	Final_2		
5	21/06/05	PAMAl_050516	Final_2		

2. Amended protocol

Protocol version Final_2

This information is the property of EMVI and all rights are reserved by EMVI
