# Supplementary material for: Safety and Immunogenicity of a Recombinant Plasmodium falciparum AMA1 Malaria Vaccine Adjuvanted with Alhydrogel™, Montanide ISA 720 or AS02
Source: PLoS One. 2008 Dec 18;3(12):e3960. doi: 10.1371/journal.pone.0003960 (PMC2602972; doi:10.1371/journal.pone.0003960)
Supplement: Amendment S4 — (0.25 MB PDF) [file pone.0003960.s006.pdf]

Signatures

I have read the amendments and agree that the trial will be conducted according to the procedures described.

Function	Name	Date	Signature
Principal Investigator	Prof Robert W. Sauerwein		
Investigator	Dr Meta Roestenberg		
E.M.V.I. Project Manager	Dr Hildur E. Blythman	16/09/05	[signature]
E.M.V.I. Executive Director	Dr Sören Jepsen	16 Sept. 2005	[signature]

This information is the property of EMVI and all rights are reserved by EMVI

Amendment N° 6

The paragraphs modified by this amendment are described below. Other paragraphs remain unchanged.

Sponsor:

European Malaria Vaccine Initiative
CIH, Bergen University, Norway
Director of Clinical and Regulatory Affairs :
Odile Leroy, MD
13 rue des 4 Vents
92380 Garches, France
Tel: +33 1 47951781
Mob: +33 6 86783149
e-mail: odile.leroy@wanadoo.fr

Change to:

Sponsor:

Hildur Ella Blythman, MD
EMVI French Office
2 allée Alfred Sisley
78160 Marly le Roi
France
Tel: +33 1 39 58 43 65
Mobile: +33 6 08 12 52 68
hildur.blythman@wanadoo.fr
www.emvi.org

Justification for the change: Dr. Odile Leroy is no longer employed by the EMVI; all responsibilities related to clinical trial management are transferred, until further notice, to Dr. Hildur E. Blythman.

Page 21

5.4.2 Storage and shipment conditions:

The acknowledgement of receipt will be dated and signed by the person in charge of product management. One copy will be kept archived, the other copy will be returned to:

Dr Odile Leroy
Clinical and Regulatory Affairs Director
European Malaria Vaccine Initiative
13 rue des 4 Vents
92380 Garches
France

This information is the property of EMVI and all rights are reserved by EMVI

Change to:

The acknowledgement of receipt will be dated and signed by the person in charge of product management. One copy will be kept archived, the other copy will be returned to:

Dr. Hildur E. Blythman
European Malaria Vaccine Initiative
2 allée Alfred Sisley
78160 Marly le Roi
France

Justification for the change: see previous item.

Page 29

6.5.2. Completion and transmission of serious adverse events reports

Study Contact for Reporting SAEs to EMVI

Dr Hildur Blythman
To be completed

Back-up Study Contact for Reporting SAEs to EMVI

Dr Odile Leroy
Tel: 33+1 47 95 17 81
Fax: 33+1 47 95 17 81
Outside office hours
Tel: 33+6 86 78 31 49
Email : odile.Leroy@wanadoo.fr

Change to:

Study Contact for Reporting SAEs to EMVI

Dr. Hildur E. Blythman
2 allée Alfred Sisley
78160 Marly le Roi
Tel: +33 1 39 58 43 65
Or: +33 6 08 12 52 68
Email: hildur.blythman@wanadoo.fr

Justification for the change: see previous item.

This information is the property of EMVI and all rights are reserved by EMVI

Amendment N° 7

Page vii

**Inclusion Criteria**	1. Age > 18 and < 45 years healthy volunteers (males or females).<br>2. General good health based on history and clinical examination.<br>3. All volunteers have to sign the informed consent form.<br>4. Negative pregnancy test.<br>5. Use of adequate contraception for females up to three months after the third injection (D140).<br>6. Reachable by phone during the whole study period (18 months).

Change to:

**Inclusion Criteria**	1. Age > 18 and < 45 years healthy male volunteers.<br>2. General good health based on history and clinical examination.<br>3. All volunteers have to sign the informed consent form.<br>4. Reachable by phone during the whole study period (18 months).

Justification for the change: In accordance with the objection raised by the RIVM (Ref 335/2005 BMT/IH – KP05031) only male volunteers will be screened for this trail. The objection is based on the fact that there is no available data on the possible reproductive health toxicity, and as the target population in Africa will be infants and children, there is no justification for exposing females of child-bearing age to the vaccine.

Page 16

4.1 Inclusion Criteria

- Age > 18 and < 45 years healthy volunteers (males or females).
- Negative pregnancy test.
- Use of adequate contraception for females up to three months after the third injection (D140).

4.2 Non-Inclusion Criteria

- Pregnant or lactating women.

Change to:

The above-listed items are deleted from the Inclusion and Non-inclusion lists of criteria.

Justification for the change: see above.

Page 32

6.6 Pregnancy

This information is the property of EMVI and all rights are reserved by EMVI

Subjects who become pregnant during the study (one month after the first dose or three months after the second dose) must not receive additional doses of study vaccine but may continue other study procedures at the discretion of the investigator.

The investigator, or his/her designee, will collect pregnancy information on any subject who becomes pregnant while participating in this study. The investigator, or his/her designee, will record pregnancy information on the Pregnancy Report Form and submit it to EMVI and GSK within 24 hours of learning of a subject's pregnancy, regardless of the adjuvant used. The investigator will inform the safety monitor as soon as possible. The safety monitor will break the code for the pregnant subject and inform GSK and EMVI about the adjuvant used. The investigator will remain to be blinded.

The subject will be followed to determine the outcome of the pregnancy. At the end of the pregnancy, whether that be full-term or prematurely, information on the status of the mother and child will be forwarded to EMVI and GSK. Generally, follow-up will be no longer than six to eight weeks following the estimated delivery date.

While pregnancy itself is not considered an AE or SAE, any pregnancy complication or elective termination of a pregnancy for medical reasons will be recorded as an AE or a SAE, as described in Section **Erreur ! Source du renvoi introuvable.** and **Erreur ! Source du renvoi introuvable.**, and will be followed as described in Section **Erreur ! Source du renvoi introuvable.**.

A spontaneous abortion is always considered to be a SAE and will be reported as described in Section **Erreur ! Source du renvoi introuvable.**. Furthermore, any SAE occurring as a result of a post-study pregnancy AND considered reasonably related in time to receipt of the investigational product by the investigator, will be reported to EMVI and GSK Biologicals as described in Section **Erreur ! Source du renvoi introuvable.**. While the investigator is not obligated to actively seek this information from former study participants, he/she may learn of a pregnancy through spontaneous reporting.

Information on pregnancies identified during the screening phase/prior to vaccine administration does not need to be collected; this information need not be communicated to safety.

Change to:

The chapter is deleted, as no longer relevant.

Justification of the amendment: see above.

Information Sheet and Informed Consent Form

CONDITIONS

Pregnancy:

This information is the property of EMVI and all rights are reserved by EMVI

Participants should not become pregnant during the 12-month study period. Participants are responsible for adequate contraception. The initial physical examination and medical examinations prior to each vaccination will include a pregnancy test.

Change to :

The above paragraph is deleted from the Informed Consent.

Justification for the change: see above.

This information is the property of EMVI and all rights are reserved by EMVI

Appendixes

1. Amendment List

Amendment number	Date	Protocol file name	Version	EC submission	
				Yes/ No	date
1	03/03/05	PAMA1_050303	Final_1	Yes	27/07/05
2	16/05/05	PAMAl_050516	Final_2	Yes	27/07/05
3	21/06/05	PAMAl_050516	Final_2	Yes	27/07/05
4	21/06/05	PAMAl_050516	Final_2	Yes	27/07/05
5	21/06/05	PAMAl_050516	Final_2	Yes	27/07/05
6	13/09/05	PAMAl_050516	Final_2		
7	13/09/05	PAMAl_050516	Final_2		

This information is the property of EMVI and all rights are reserved by EMVI
