# Supplementary material for: Safety and Immunogenicity of a Recombinant Plasmodium falciparum AMA1 Malaria Vaccine Adjuvanted with Alhydrogel™, Montanide ISA 720 or AS02
Source: PLoS One. 2008 Dec 18;3(12):e3960. doi: 10.1371/journal.pone.0003960 (PMC2602972; doi:10.1371/journal.pone.0003960)
Supplement: Amendment S5 — (0.52 MB PDF) [file pone.0003960.s007.pdf]

Signatures

I have read the amendments and agree that the trial will be conducted according to the procedures described.

Function	Name	Date	Signature
Principal Investigator	Prof Robert W. Sauerwein		
Investigator	Dr Meta Roestenberg		
E.M.V.I. Project Manager	Dr Hildur E. Blythman	21/10/05	[signature]
E.M.V.I. Executive Director	Dr Sören Jepsen	24/10/05	[signature]

This information is the property of EMVI and all rights are reserved by EMVI

Amendment N° 8

The paragraphs modified by this amendment are described below. Other paragraphs remain unchanged.

Page 37

7.4.4 Statistical Methods

The analysis shall be descriptive; the sample size does not allow any comparison between groups. For categorical variables, frequency distributions, by vaccination group, will be presented. For continuous variables, box-whisker plots, medians, inter-quartile ranges and ranges will be presented by vaccination group.

The analysis plan will be available before the lock of the data base for the interim analysis after the second injection of vaccine.

Change to:

7.4.4 Statistical Methods

The analysis shall be descriptive; the sample size does not allow any comparison between groups. For categorical variables, frequency distributions, by vaccination group, will be presented. For continuous variables, box-whisker plots, medians, inter-quartile ranges and ranges will be presented by vaccination group.

The analysis plan will be available before the lock of the data base for the interim analysis after the **third** injection of vaccine. **The interim analysis will be performed with data collected up to Visit 16 (Day 70).**

Justification for the change:

Since the vaccination schedule was changed (cf. Amendment N° 4), the 3rd vaccination will be given 28 days after the 2nd vaccination, instead of after 84 days; therefore, it is preferable to perform the interim analysis soon after the 3rd vaccination. The purpose of this interim analysis is to allow the choice of the best vaccination regime for the follow-up Phase Ib trial to be performed in Mali.

This information is the property of EMVI and all rights are reserved by EMVI

Appendixes

1. Amendment List

Amendment number	Date	Protocol file name	Version	EC submission	
				Yes/ No	date
1	03/03/05	PAMA1_050303	Final_1	Yes	27/05/05
2	16/05/05	PAMAl_050516	Final_2	Yes	27/05/05
3	21/06/05	PAMAl_050516	Final_2	Yes	27/05/05
4	21/06/05	PAMAl_050516	Final_2	Yes	27/05/05
5	21/06/05	PAMAl_050516	Final_2	Yes	27/05/05
6	13/09/05	PAMAl_050516	Final_2	No	
7	13/09/05	PAMAl_050516	Final_2	No	
8	20/10/05	PAMA1_050516	Final_2	No	

This information is the property of EMVI and all rights are reserved by EMVI
