# Supplementary material for: Safety and Immunogenicity of a Recombinant Plasmodium falciparum AMA1 Malaria Vaccine Adjuvanted with Alhydrogel™, Montanide ISA 720 or AS02
Source: PLoS One. 2008 Dec 18;3(12):e3960. doi: 10.1371/journal.pone.0003960 (PMC2602972; doi:10.1371/journal.pone.0003960)
Supplement: Amendment S6 — (1.06 MB PDF) [file pone.0003960.s008.pdf]

Signat

I have read the amendments and agree that the trial will be conducted according to the procedures described.

Function	Name	Date	Signature
Principal Investigator	Prof Robert W. Sauerwein	21/04/06	[signature]
Investigator	Dr Meta Roestenberg	21/04/06	[signature]
E.M.V.I. Project Manager	Dr Hildur E. Blythman	17/04/2006	[signature]
E.M.V.I. Executive Director	Dr Sören Jepsen	18/04/2006	[signature]

This information is the property of EMVI and all rights are reserved by EMVI

Amendment N° 9

This amendment relates to previous Amendments:
N° 1 (dated 03/03/2005) concerning the **Informed Consent**
N° 4 (dated 21/06/2005) concerning the **change of vaccination schedules**
N° 8 (dated 20/10/2005) concerning the **timing of the Interim analysis**

Subject Information and Informed Consent

Purpose

Previous version :

The most important purpose of this study is to evaluate if human beings can safely use the vaccine. For this purpose, the symptoms caused by the injections, the complaints of volunteers and the laboratory results will be evaluated. The vaccine will be tested in two doses.
Secondly, it is important to see how the human immune system reacts to the vaccine, in order to estimate its effectiveness.
To most vaccines we know, adjuvants are added, to make the vaccine more effective. As it is not yet clear which adjuvant is most effective in vaccinations against malaria and the AMA-1 vaccine, three different adjuvants will be compared in this study.
To test both the two different doses and the three different adjuvants, volunteers will be allocated to 6 different groups of 10 people at random. Every group will receive its own combination of dose and adjuvant.

New version :

The most important purpose of this study is to evaluate if human beings can safely use the vaccine AMA-1. For this purpose, the symptoms caused by the injections, the complaints of volunteers and the laboratory results will be evaluated. The vaccine AMA-1 will be tested in two doses.
Secondly, it is important to see how the human immune system reacts to the vaccine, in order to estimate its effectiveness
Adjuvants are added to vaccines to make them more effective. In this study we aim to compare three different adjuvants. The testing of two adjuvants has been completed. AMA-1 will now be studied in 20 volunteers in combination with ASO2, a third adjuvant.

This information is the property of EMVI and all rights are reserved by EMVI

Design

Previous version:

In total 60 volunteers will be participating in this study. They will be divided into 6 groups as prescribed. Every group will receive a different dosage and adjuvant. The study will take 12 months according to the following scheme:

New version:

In total 20 volunteers will be participating in this study. They will be divided into 2 groups . Both groups will receive a different dosage AMA-1 in combination with ASO2. The study will take 12 months according to the following scheme:

AMA-1 vaccine

Previous version:

AMA-1 is a protein, part of the parasite wall. Malaria parasites use AMA-1 to enter the red blood cell. Previous research in animals showed that an immune response to AMA-1 prevents the parasite from entering the red blood cell, so that it is not be able to multiply.
Aluminum hydroxide is one of the adjuvants that will be tested in this study. It is the adjuvant that is most common in vaccines.
Montanide 720 is another adjuvant, but it has only in the last years been introduced to the market, and is now an international approved adjuvant. It has also been enrolled in several malaria vaccine trials before, which have shown reasonable results.
ASO2, at last, is an experimental adjuvant that has recently booked good results in another malaria vaccine trial in Africa.
Both Montanide 720 and ASO2 have been approved for use in humans.

New version:

AMA-1 is a protein, part of the parasite wall. Malaria parasites use AMA-1 to enter the red blood cell. Previous research in animals showed that an immune response to AMA-1 prevents the parasite from entering the red blood cell, so that it is not be able to multiply.

ASO2 is an experimental adjuvant that has been approved for use in humans. Recently, several hundred people have been immunised with good results as part of a malaria vaccine trial in Africa.

This information is the property of EMVI and all rights are reserved by EMVI

Safety

Previous version:

Many animal experiments have been performed with the candidate malaria vaccine AMA-1. However, it has never been tested with humans. Based on this information, the Committee of Humane Research of the region Nijmegen-Arnhem, has approved of this study.
Previous studies in different animals did not show serious side effects on vaccination with AMA-1. In combination with the adjuvants Aluminum hydroxide and Montanide 720 there may be mild side effects. Typical side effects that may occur with these adjuvants are local swelling, redness of the skin and swelling.

New version:

In animal experiments with AMA-1 no serious side effects have been observed. Based on this information, the Committee of Human Research of the region Nijmegen-Arnhem, has approved of this study. Recently, two trials have been published with AMA-1, in which no serious and/or unexpected side effects of AMA-1 were observed.
The AS02 adjuvant has not been tested with AMA-1 in humans. In trials with another malaria vaccine in combination with ASO2, the most frequently observed side effects were pain , myalgia and fatigue, usually resolving within a few days.
In the first part of this study the following side effects were observed: erythema, induration and sterile abcesses. The side effects didn't have consequences for usual daily activities of the volunteers.

Questions?

Previous version:

If, after reading the above information, you still have questions about (participating in) the AMA-1 study, you can always contact:

Dr. M. Roestenberg
Medical investigator, Medical Microbiology
Universitair Medisch Centrum Nijmegen
Tel: 024 361 95 15
Email: malariavaccin@mmb.umcn.nl

This information is the property of EMVI and all rights are reserved by EMVI

New version :

If, after reading the above information, you still have questions about (participating in) the AMA-1 study, you can always contact:

Dr. Erik de Jonge
Medical investigator, Medical Microbiology
Universitair Medisch Centrum Nijmegen
Tel: 024 361 95 15
Email: malariavaccin@mmb.umcn.nl

Justification for the changes :

For internal logistics reasons, the trial started as planned, but only with the groups receiving the vaccine containing either Alum or Montanide ISA720; the group of subjects that should receive the vaccine containing AS02A (cf Chapter 3, page 12) will be enrolled later.

The Informed Consent and Advertisement have been changed, to inform the volunteers that only one adjuvant (AS02A) will be tested.

An additional member of staff, Dr. Erik de Jonge, will participate in the screening procedures, as well as in the follow-up visits of the volunteers recruited in the AS02 arms of the trial.

Page 37

7.4.4 Statistical Methods

The analysis plan will be available before the lock of the data base for the interim analysis after the third injection of vaccine. The interim analysis will be performed with data collected up to Visit 16 (Day 70).

Change to:

7.4.4 Statistical Methods

The analysis plan will be available before the lock of the data base for the interim analysis after the third injection of vaccine. The interim analysis will be performed with data collected up to **Visit 17 (Day 84: one month after the 3rd vaccination).**

This information is the property of EMVI and all rights are reserved by EMVI

Justification for the change:

The purpose of this interim analysis is to allow the choice of the best vaccination regime for the follow-up Phase Ib trial to be performed in Mali. To make the best choice, the results of the Immune response to the three vaccine formulations (measured by Immunological tests) will be available only after V17, when blood samples are collected from the volunteers.

* *

Amendment N° 10:

Page 18:

Chapter 5.1.2: Formulation

Composition

- Active ingredient: (*Pf*AMA-1-FVO[25-545]): 120µg

AS02A formulation

AS02A is an adjuvant system containing 3-deacylated monophosphoryl lipid A (MPL®) and a purified saponin in an oil-water emulsion (QS21).

Two dosages will be prepared as follows:

- 50 µg Dose

Dissolve 120 µg AMA-1 in a volume of 1.2 mL AS02A. This gives a concentration of 100 µg/mL. Final volume will be 1.2 mL, which is sufficient for one 0.5 mL dose.

- 10 µg dose

Dissolve 120 µg AMA-1 in a volume of 6 mL AS02A. This gives a concentration of 20 µg/mL. Final volume will be 6 mL, which is sufficient for one 0.5 mL dose.

Change to:

Chapter 5.1.2: Formulation

Composition

- Active ingredient: (*Pf*AMA-1-FVO[25-545]): 62,5 µg

This information is the property of EMVI and all rights are reserved by EMVI

AS02A formulation

AS02A is an adjuvant system containing 3-deacylated monophosphoryl lipid A (MPL®) and a purified saponin in an oil-water emulsion (QS21).

Two dosages will be prepared as follows:

- 50 µg Dose

Dissolve 62,5 µg AMA-1 in a volume of 0,6 mL AS02A. This gives a concentration of 100 µg/mL. Final volume will be 0,6 mL, which is sufficient for one 0.5 mL dose.

- 10 µg dose

Dissolve 62,5 µg AMA-1 in a volume of 3 mL AS02A. This gives a concentration of 20 µg/mL. Final volume will be 3 mL, which is sufficient for 5 doses of 0.5 mL/dose.

Justification for the change:

An improved formulation has been developed, and tested for its use with the Adjuvant AS02A; each vial contains 62,5 µg of the active ingredient, instead of 120 µg.

Appendixes

1. List of Amendments

Amendment number	Date	Protocol file name	Version	EC submission	
				Yes/ No	Date of approval
1	03/03/05	PAMA1_050303	Final_1	Yes	31/10/05
2	16/05/05	PAMA1_050516	Final_2	Yes	31/10/05
3	21/06/05	PAMA1_050516	Final_2	Yes	31/10/05
4	21/06/05	PAMA1_050516	Final_2	Yes	31/10/05
5	21/06/05	PAMA1_050516	Final_2	Yes	31/10/05
6	13/09/05	PAMA1_050516	Final_2	Yes	31/10/05
7	13/09/05	PAMA1_050516	Final_2	Yes	31/10/05
8	20/10/05	PAMA1_050516	Final_2	Yes	17/11/05
9	17/04/05	PAMA1_050516	Final_2		
10	17/04/05	PAMA1_050516	Final_2		

This information is the property of EMVI and all rights are reserved by EMVI
